# Atomically accurate site-specific ligand tailoring of highly acid- and alkali-resistant Ti(iv)-based metallamacrocycle for enhanced CO_2_ photoreduction†

**DOI:** 10.1039/d3sc06046b

**Published:** 2023-11-30

**Authors:** Yi-Qi Tian, Lin-Fang Dai, Wen-Lei Mu, Wei-Dong Yu, Jun Yan, Chao Liu

**Affiliations:** a Hunan Provincial Key Laboratory of Chemical Power Sources, College of Chemistry and Chemical Engineering, Central South University Changsha 410083 Hunan P. R. China chaoliu@csu.edu.cn; b China College of Science, Hunan University of Technology and Business Changsha 410000 P. R. China

## Abstract

Skillfully engineering surface ligands at specific sites within robust clusters presents both a formidable challenge and a captivating opportunity. Herein we unveil an unprecedented titanium-oxo cluster: a calix[8]arene-stabilized metallamacrocycle (Ti_16_L_4_), uniquely crafted through the fusion of four “core–shell” {Ti_4_@(TBC[8])(L)} subunits with four oxalate moieties. Notably, this cluster showcases an exceptional level of chemical stability, retaining its crystalline integrity even when immersed in highly concentrated acid (1 M HNO_3_) and alkali (20 M NaOH). The macrocycle's surface unveils four specific, customizable μ_2_-bridging sites, primed to accommodate diverse carboxylate ligands. This adaptability is highlighted through deliberate modifications achieved by alternating crystal soaking in alkali and carboxylic acid solutions. Furthermore, Ti_16_L_4_ macrocycles autonomously self-assemble into one-dimensional nanotubes, which subsequently organize into three distinct solid phases, contingent upon the specific nature of the four μ_2_-bridging ligands. Notably, the Ti_16_L_4_ exhibit a remarkable capacity for photocatalytic activity in selectively reducing CO_2_ to CO. Exploiting the macrocycle's modifiable shell yields a significant boost in performance, achieving an exceptional maximum CO release rate of 4.047 ± 0.243 mmol g^−1^ h^−1^. This study serves as a striking testament to the latent potential of precision-guided surface ligand manipulation within robust clusters, while also underpinning a platform for producing microporous materials endowed with a myriad of surface functionalities.

## Abstract 2

An eye-catching titanium-oxo cluster, embellished with a calix[8]arene-stabilized metallamacrocycle structure, remains resilient in the face of harsh acid and alkali solutions.

## Graphical abstract

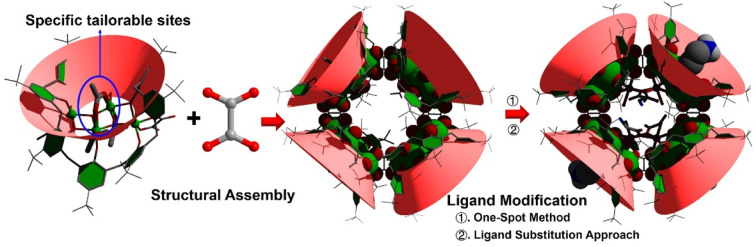

## Introduction

Amidst the evolving landscape of advanced materials, titania nanomaterials have garnered significant interest, particularly for their promising applications as semiconductor photocatalysts in solar energy conversion.^1–3^ The allure of structurally precise titanium-oxo clusters (TOCs) stems from their tunable geometry and intriguing photoelectrical properties. Emerging as a captivating model, these attributes not only unravel intricate structure–property correlations within bulk TiO_2_ phases at the atomic scale, but also offer valuable structural and spectroscopic insights into the surface properties of TiO_2_.^4–10^ Unlike conventional TiO_2_, TOCs exhibit a rich diversity of structural types, carrying with them a distinctive palette of traits such as stability, semiconductor-like attributes, light absorption capacity, and band structure. These features are expertly modulated by introducing various functionalized organic ligands to achieve tailored modifications.^11–21^ Yet, the pursuit of precision in ligand modifications on specific TOCs comes with its challenges, requiring the careful negotiation of unstable coordination sites while upholding the core's inorganic integrity. Regrettably, altering protective ligands often triggers partial or complete dissection, resection, or structural reconstruction of the clusters.^22,23^ To surmount this hurdle, the creation of exceptionally stable TOCs emerges as a pivotal prerequisite. Enter the realm of macrometallocycles (MMCs), renowned for their formidable stability rooted in robust ring structures. This inherent resilience safeguards the chemical essence of TOCs, even under rigorous conditions, paving the way for subsequent adaptations. Additionally, another approach involves enveloping the cluster core with bulky polydentate macrocyclic ligands, exemplified by calixarenes.^24–26^ These intricate macrocycles, composed of multiple phenol units, have a storied history in orchestrating diverse metallo-supramolecular architectures.^27–33^ By fusing TOCs with a macrocyclic scaffold shielded by calixarene ligands, an uncharted territory opens to explore the impact of ligand tailoring at precise sites on their physicochemical attributes. While cyclic clusters abound,^34–40^ the realm of Ti^IV^-based rings remains relatively untapped due to the hydrolysis susceptibility of Ti^4+^ ions. Notably, calixarene-stabilized Ti^IV^-MMCs have yet to grace the literature. The envisioned assembly of atomically precise MMCs of Ti^IV^, secured by calixarene guardians, holds immense promise for ushering rational design and optimizing performance across future applications.

**Scheme 1:**
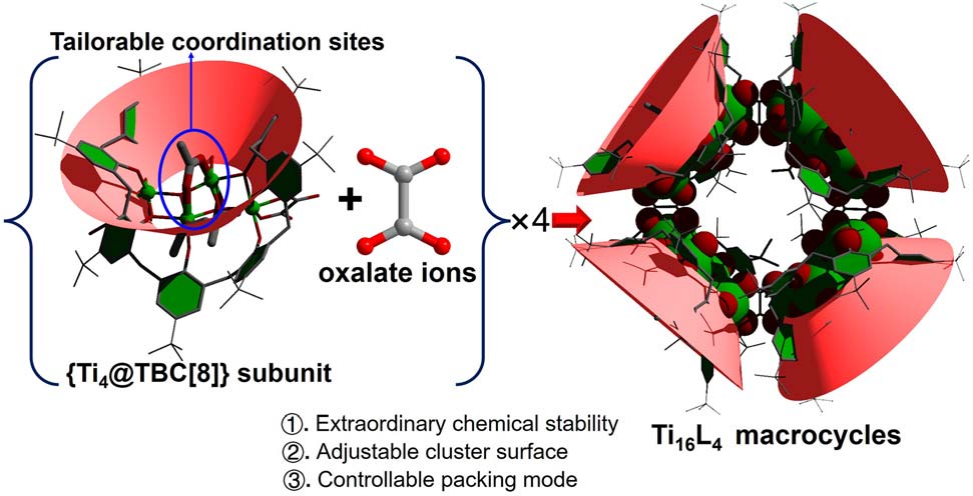
Illustration of the assembly of the Ti_16_L_4_ macrocycle with specific tailorable sites.

Herein, an exceptionally stable *p-tert*-butylcalix[8]arene-protected Ti^IV^-based metallamacrocycle (MMC), denoted as [H_4_Ti_16_O_8_(TBC[8])_4_(Oa)_4_(Ac)_4_(^i^PrO)_8_], was synthesized. This MMC showcases remarkable resilience, displaying resistance against an array of organic solvents, concentrated acid (1 M HNO_3_), and alkali (20 M NaOH), thus establishing itself as a paramount example of cluster stability. The gigantic cluster has a “donut” shape with an inner diameter of 12 Å, an outer diameter of 30 Å, and a height of 18 Å, making it the largest known TOC in the metal-calixarene system. A pivotal discovery lies in the identification of four modifiable coordination sites on the Ti_16_L_4_ surface, which has led to further exploration of its exchange activity and applications (Scheme 1). These sites, intriguingly, offer two distinct avenues for functionalization: (1) utilizing a one-pot synthesis method, the μ_2_-bridging sites can be occupied by acetate and Cl^−^. The ratio of these ligands can be precisely tailored from 4 : 0 to 2 : 2, and further to 0 : 4. Noteworthy is the emergence of a 3D network in Ti_16_L_4–_3, modified with four Cl^−^, yielding infinite cylindrical channels with diameters of approximately 2 nm. (2) An alternative post-modification strategy involves a sequential immersion of the cluster in concentrated alkali and carboxylic acid solutions, facilitating reversible exchange of the μ_2_-bridging sites by OH^−^ and diverse carboxylates. The molecular-level understanding of this exchange process is unveiled through crystal-to-crystal diffraction studies, shedding light on structural transformations and ligand dynamics. Significantly, modifying the Ti_16_L_4_ shell distinctly influences physicochemical attributes, encompassing photocurrent response, hydrophilicity and energy levels, with ultimate implications for the photocatalytic CO_2_ reduction potential of the clusters. The ability to organically modify the shell while preserving macrocycle integrity introduces a pioneering avenue for fine-tuning chemical properties, setting the stage for these promising Ti^IV^-based MMCs to be harnessed across diverse applications.

## Results and discussion

**Fig. 1:**
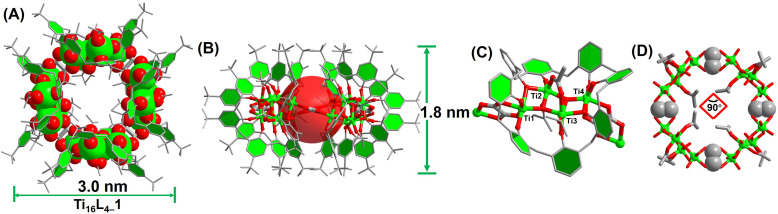
Structural depictions of Ti_16_L_4–_1. (A) and (B) Overhead and lateral perspectives; (C) configuration of the {Ti_4_@TBC[8]} subunit; (D) arrangement of the {Ti_16_} nanoring.

The synthesis of Ti_16_L_4–_1 entailed a one-pot solvothermal reaction involving TBC[8], oxalic acid (H_2_Oa), and Eu(Ac)_3_ in conjunction with Ti(O^i^Pr)_4_, conducted within acetonitrile (CH_3_CN) at 120 °C for 72 hours. This procedure yielded red crystals with a notable 65% yield and consistent reproducibility. The ensuing single-crystal X-ray diffraction (SCXRD) analysis unveiled Ti_16_L_4–_1's monoclinic system crystallization in space group *C*2/*c*.^41^ The composition was precisely identified as [H_4_Ti_16_O_8_(TBC[8])_4_(Oa)_4_(Ac)_4_(^i^PrO)_8_]. X-ray photoelectron spectroscopy (XPS) analysis further validated the presence of Ti^4+^. The Ti 2p spectrum exhibited distinct regions at Ti 2p_1/2_ (*ca.* 464.9 eV) and Ti 2p_3/2_ (*ca.* 459.0 eV) with a binding energy separation of 5.9 eV, confirming the existence of Ti^4+^ (Fig. S32†). A comprehensive structural analysis elucidated the intricate architecture of the cluster. It emerged as a “core–shell” assembly comprising four {Ti_4_@TBC[8]} subunits interconnected by four oxalate ligands (Fig. 1A and B). Within each {Ti_4_@TBC[8]} subunit, four Ti^4+^ ions formed a central {Ti_4_O_2_} core connected by μ_3_-O^2−^ ions, encased within the TBC[8] cavity (Fig. 1C). The TBC[8] molecule exhibits two distinct bonding modes for its eight phenoxide groups. Six of these groups are individually bonded to one Ti^4+^, while the remaining two groups are bridged by two Ti^4+^ in a μ_2_–κ^2^(O) fashion. The {Ti_4_O_2_} core showcased two distinct sets of Ti^4+^ sites. The Ti(2) and Ti(3) sites possess identical coordination environments, each coordinated by two phenol O, two μ_3_-O^2−^ ions, and one ^i^PrO^−^ group. An additional acetate linked these two Ti^4+^ in a μ_2_-(O,O′) bridging fashion. Additionally, Ti(1) and Ti(4) exhibited equivalent coordination environments immobilized within the TBC[8] cavity. TBC[8]'s flexibility allowed the attachment of an Oa^2−^ ligand to Ti(1) and Ti(4), while the oxalate ligand adopted a μ_2_-(O,O,O′,O′) mode, bridging two {Ti_4_@TBC[8]} subunits into a dimeric structure. This unique oxalate binding played a pivotal role in forming the closed-loop structure. Four oxalate ligands vertically bridged four {Ti_4_@TBC[8]} units, culminating in a quadrilateral macrocycle characterized by a 90° internal angle (Fig. 1D). Notably, this macrocycle exhibited an outer diameter of 30 Å and a height of 18 Å, with internal voids measuring an inner diameter of 12 Å and a window size of 7.5 Å, all determined by structural features.

### Ligand modification

#### One-spot method

**Fig. 2:**
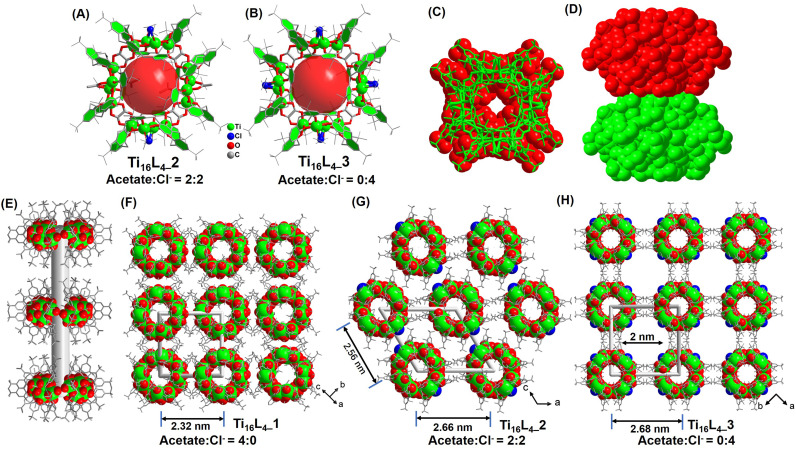
(A) and (B) Configuration of the Ti_16_L_4–_2/3 macrometallocycles (MMCs); (C) and (D) proximity arrangement of two Ti_16_L_4_ macrocycles; (E) formation of the nanotube within Ti_16_L_4_; (F)–(H) three distinct patterns of nanotube arrangement, including square and hexagonal arrays.

As demonstrated in prior studies, the unique structural motifs of the four {Ti_4_@TBC[8]} subunits encompass embellished Ti2 and Ti3 sites, each adorned with a single acetate and two weakly coordinated ^i^PrO^−^ groups. Intriguingly, through a deliberate alteration involving the substitution of Eu(Ac)_3_ with a judicious blend of Eu(Ac)_3_ and EuCl_3_, or by sequential addition of EuCl_3_ during the synthetic process, distinct crystalline entities, Ti_16_L_4–_2 and Ti_16_L_4–_3, are successfully synthesized.^42,43^ For Ti_16_L_4–_2, the architectural formulation reads [H_4_Ti_16_O_8_(TBC[8])_4_(Oa)_4_(Ac)_2_Cl_2_(^i^PrO)_8_], while Ti_16_L_4–_3 is denoted by the composition [H_4_Ti_16_O_8_(TBC[8])_4_(Oa)_4_Cl_4_(^i^PrO)_8_]. Remarkably, these two clusters mirror the macrocyclic framework of Ti_16_L_4–_1, while diverging in their surface ligands. A notable feature is the dynamic occupancy of the four μ_2_-bridging sites within the macrocycles, where acetate and Cl^−^ find a variable equilibrium. The precise modulation of this ligand interplay is evident, transitioning from a 4 : 0 ratio in Ti_16_L_4–_1, to a balanced 2 : 2 ratio in Ti_16_L_4–_2 (Fig. 2A), culminating in an exclusive 0 : 4 ratio in Ti_16_L_4–_3 (Fig. 2B).

Beyond the intricate geometries that clusters adopt, the interplay between surface ligands emerges as a pivotal determinant of their crystalline packing and consequential material attributes. Mastery over these multifaceted factors remains a formidable task. Our study, however, illuminates a path to precision control in this arena. Strikingly, while the shared macrocyclic scaffold remains constant, variations in surface ligands orchestrate disparate packing configurations within the crystal lattice of Ti_16_L_4–_1/2/3. Within all phases, the metallacycles ingeniously arrange face-to-face, thus sculpting 1D nanotubes that harmonize through van der Waals forces, culminating in their assemblage (Fig. 2C and D). The meticulous orchestration of this assembly begins by staggering neighboring nanotubes to alleviate steric hindrance stemming from *tert*-butyl moieties of adjacent macrocycles (Fig. 2E). This choreographed dance then evolves into a parallel stacking, fashioning an assorted array of 3D architectures. Remarkably, Ti_16_L_4–_1's nanotubes organize into a square matrix (Fig. 2F), whereas Ti_16_L_4–_2's nanotubes adopt a hexagonal pattern (Fig. 2G). This intriguing divergence traces its origins back to the precise occupancy of the four μ_2_-bridging sites. The substitution of acetate with Cl^−^ profoundly impacts C–H⋯C–H interactions among the clusters. Additionally, the presence of polar Ti–Cl bonds endows the surface of Ti_16_L_4–_2, now embellished with two Cl^−^, with a discernible negative charge. This newfound electrostatic repulsion between the nanotubes impels their separation within the lattice. Evidently, the centroid distance between adjacent nanotubes escalates from 2.32 nm (as observed in Ti_16_L_4–_1) to 2.56 and 2.66 nm in succession. This captivating phenomenon finds further exemplification in the assembly motif of Ti_16_L_4–_3, wherein all four μ_2_-bridging sites embrace Cl^−^, ensuing in yet another square arrangement of macrocycles. Notably, a subtle 45° rotation between neighboring nanotubes in Ti_16_L_4–_1 mitigates the dominant electrostatic repulsion, paving the way for the emergence of 1D channels along the *c*-axis. These channels, a noteworthy 2 × 2 nm in dimension, define Ti_16_L_4–_3's unique character (Fig. 2H). The resplendent novelty of these findings lies in the precise manipulation of ligand dynamics to orchestrate such diverse and intricate packing phenomena.

**Fig. 3:**
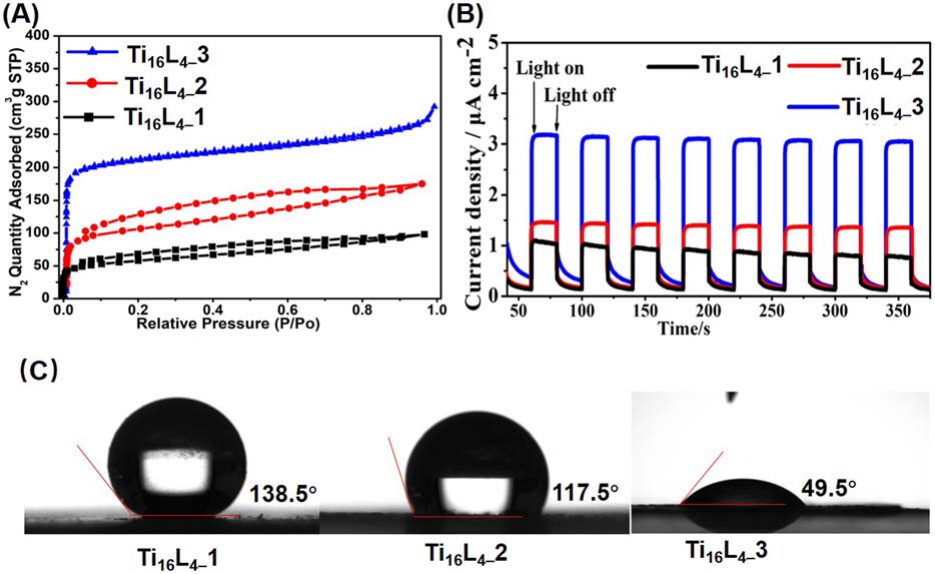
(A) N_2_ adsorption/desorption isotherm recorded at 77 K; (B) transient photocurrent responses of Ti_16_L_4_ captured during Xe lamp irradiation in a 0.2 M Na_2_SO_4_ electrolyte solution; (C) comparative assessment of the wettabilities across Ti_16_L_4–_1, Ti_16_L_4–_2 and Ti_16_L_4–_3.

The intricate interlocking of Ti_16_L_4_ macrocycles intricately assembles into meticulously ordered nanotubular frameworks across all three distinct phases. To validate the porosity of these architectures, we conducted N_2_ adsorption/desorption analyses that unveiled characteristic type I isotherms for each phase, unmistakably indicating their inherent porous nature. Concretely, our evaluation yielded Brunauer–Emmett–Teller (BET) surface areas of 209.24 m^2^ g^−1^ for Ti_16_L_4–_1, 389.92 m^2^ g^−1^ for Ti_16_L_4–_2, and a remarkable 813.46 m^2^ g^−1^ for Ti_16_L_4–_3 (Fig. 3A). The underlying absorbance potential was corroborated by diffuse reflectance spectra, revealing robust light absorption in the visible spectrum (Fig. S39†). Capitalizing on this light absorption attribute, we ventured into assessing the visible-light-driven photocurrent responses of Ti_16_L_4_. Through repeated irradiation cycles, steady photocurrent responses were uniformly observed across all clusters. Notably, the substitution of acetate with Cl^−^ yielded an enhancement in photocurrent density. Among the clusters, Ti_16_L_4–_3 emerged as the standout, demonstrating the most optimal photocurrent response. Its recorded current density surged to an impressive 3.2 μA cm^−2^, which was approximately threefold higher than that of Ti_16_L_4–_1 (1.0 μA cm^−2^) (Fig. 3B). Interestingly, the hydrophobicity of Ti_16_L_4_ crystals emerged as an outcome intricately linked to their specific stacking arrangements. Evidently, water droplets exhibited distinct behaviors on the surfaces of Ti_16_L_4–_1/2 powders, maintaining rounded shapes with contact angles measuring 138.5° and 117.5°, respectively (Fig. 3C). In a striking departure, the contact angle observed for Ti_16_L_4–_3 was notably reduced to a mere 49.5°, indicative of its pronounced hydrophilic character. This intriguing contrast owes its origins to the presence of polar Ti–Cl bonds and the substantial presence of expansive cylindrical channels within the structural matrix, collectively orchestrating this distinctive behavior.

**Fig. 4:**
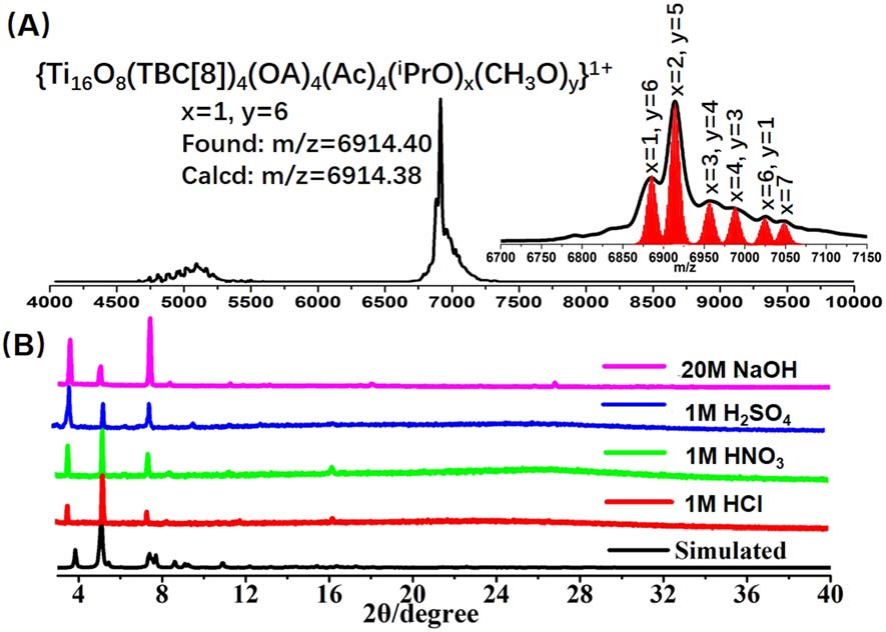
(A) Positive-mode MALDI-TOF-MS spectrum depicting Ti_16_L_4–_1 in CHCl_3_ solution; (B) PXRD patterns of Ti_16_L_4–_1 acquired during acid- and base-stability assessments. Remarkably, Ti_16_L_4–_1 exhibits robustness even after prolonged exposure to 1 M HCl, HNO_3_, H_2_SO_4_, and 20 M NaOH for 24 hours.

The stability of the nanocluster emerges as a pivotal facet, particularly in the context of post-synthetic modifications, warranting meticulous consideration. Significantly, the calixarene modification of the Ti_16_L_4_ confers a profound enhancement in chemical stability. Evidently, our PXRD analysis substantiated the robust stability of the Ti_16_L_4–_1 crystal across an array of solvents such as toluene, methanol, CH_3_CN, and DMF (Fig. S26†). To further probe its solution behavior, we harnessed matrix-assisted laser desorption/ionization time-of-flight mass spectrometry (MALDI-TOF-MS) on Ti_16_L_4–_1 dissolved within a mixed solvent of CHCl_3_ and MeOH (Fig. 4A). Encouragingly, the dominant peaks in the spectrum unequivocally corresponded to species like [H_4_Ti_16_O_8_(TBC[8])_4_(Oa)_4_(Ac)_4_(^i^PrO)_*x*_(MeO)_*y*_]^+^ (*x* + *y* = 7). This finding underscores that, while retaining the overall architecture of Ti_16_L_4_, the ^i^PrO^−^ on the cluster exhibit high exchangeability with MeO^−^ ions, while the chelating acetate sites remain resolutely stable. Dynamic light scattering measurements further confirmed a singular size distribution with an average diameter of 4.5 nm, a fitting match to the cluster size, concretely affirming the presence of well-defined Ti_16_L_4_ entities in solution (Fig. S37†). Another distinctive feature of Ti_16_L_4–_1 crystals comes to the fore in the realm of water stability—an attribute often fraught with challenges in many cluster crystals. In stark contrast, the exceptional pH stability of Ti_16_L_4–_1 crystals prevails over a broad pH values spanning from 1 to 14 (Fig. S27†). Subsequent testing reinforces this extraordinary stability, with the crystals remaining unaltered even in the face of concentrated acids (1 M HCl, 1 M H_2_SO_4_, and 1 M HNO_3_) and alkali (20 M NaOH) over a 24 hour period (Fig. 4B). This robust endurance finds its basis in the hydrophobic calixarene outer chamber enveloping Ti_16_L_4_, serving as an effective barrier against acid/alkali corrosion of the hydrophilic Ti^4+^. This ingeniously designed spatial protection imparts a remarkably high level of water stability to the cluster.

#### Post-synthetic modification

**Fig. 5:**
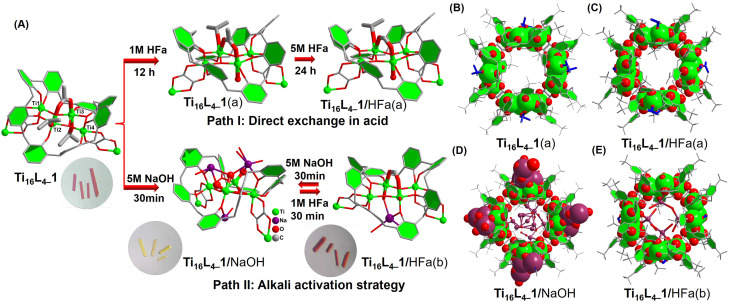
(A) Schematic representation of two different post-modification paths: (B–E) crystal structures of the derived clusters of Ti_16_L_4–_1.

Through an in-depth analysis of the Ti_16_L_4–_1 structure, we find that it does present 4 × 3 = 12 labile coordination sites, comprising four μ_2_-acetate sites and eight weakly coordinated ^i^PrO^−^ sites. Complementary mass spectrometry experiments have intriguingly demonstrated the dynamic nature of ^i^PrO^−^ in solution, allowing rapid replacement by MeO^−^. Eager to bolster the surface functionality of Ti_16_L_4_, our focus shifted towards investigating the substitution of the four μ_2_-acetate sites through post-synthetic modification. The exceptional stability of Ti_16_L_4_ crystals offered us an opportunity to pinpoint the exchange sites at the molecular level, employing the powerful SCXRD techniques that provided nuanced insights into the intricacies of the ligand exchange process (Fig. 5A). Embarking on a journey of formic acid (HFa) immersion, a carefully orchestrated sequence unveiled fascinating revelations. SCXRD scrutiny after immersing the Ti_16_L_4–_1 crystal in 1 M formic acid for 12 h highlighted the replacement of only the four innermost labile ^i^PrO^−^ sites within the ring, now replaced by H_2_O molecules (yielding Ti_16_L_4–_1(a),^44^Fig. 5B). Elevated HFa concentration (5 M) and prolonged soaking (24 h) triggered further substitution, this time of the four acetate sites by formate ligands (resulting in Ti_16_L_4–_1/HFa(a), Fig. 5C). Notably, the stability of the μ_2_-(O,O′) chelating form conferred a thermodynamically challenging nature to the exchange of these four acetate sites, mandating a high-concentration, extended duration immersion for successful replacement. In parallel, the terminal ^i^PrO^−^ sites, inherently labile, seamlessly surrendered to H_2_O molecules, while the external ^i^PrO^−^ sites, shrouded within the hydrophobic outer calixarene cavity, remained impervious to H_2_O access. An interesting color transformation emerged from the immersion of Ti_16_L_4–_1 crystals in 5 M NaOH, with a rapid shift from red to yellow within 30 minutes. SCXRD analysis of the resulting yellow crystal (now Ti_16_L_4–_1/NaOH,^45^Fig. 5D) illuminated a thorough overhaul—complete replacement of all four acetates and eight ^i^PrO^−^ ligands by OH^−^, bolstered by numerous Na^+^ that connected through OH^−^ bridging. Subsequent immersion in 1 M HFa for 30 minutes not only restored the red hue but also the crystalline phase (now Ti_16_L_4–_1/HFa(b)).^46^ SCXRD verification elucidated the reintroduction of the four μ_2_-sites *via* formate ions, reverting to the μ_2_-(O,O′) configuration (Fig. 5E). Encouragingly, this intricate exchange process proved fully reversible, evidenced by the cycle of color transformation—the red crystals of Ti_16_L_4–_1/HFa(b) reverting to yellow during re-immersion in 5 M NaOH, indicative of formate ions being replaced by OH^−^ ions. Impressively, even after undergoing five alternating cycles of immersion in 1 M HFa and 5 M NaOH, the crystal retained its structural stability (Fig. S28†). Remarkably, these color shifts in response to acid and alkali serve as tangible indicators of the comprehensiveness of the ligand exchange process—a visually compelling testament to the groundbreaking strides of our work.

**Fig. 6:**
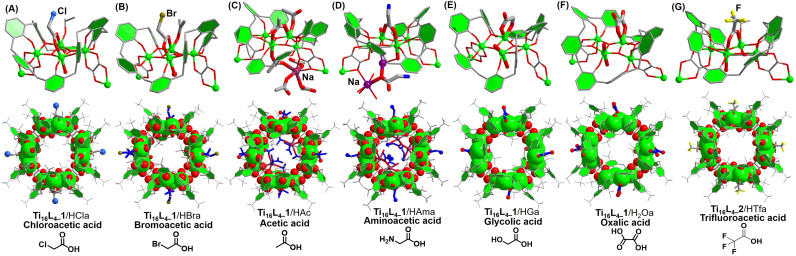
(A)–(G) Crystal structures of modified clusters derived from Ti_16_L_4_*via* post-modification pathways.

Drawing from the above insightful findings, the substitution of the four μ_2_-acetate sites within Ti_16_L_4_ emerges as a dynamic process, amenable to two distinct pathways. The first, path I, involves the direct immersion of crystals in a concentrated formic acid solution for a minimum of 24 hours. The second, path II, unfolds as a sequential immersion, commencing with a solution of NaOH, followed by a carboxylic acid. This latter path capitalizes on acid–base neutralization reactions, rendering the exchange process swift, completed within a mere hour. Evaluating the efficacy of these approaches, we embarked on a mission to extend the carboxylate modifications on the Ti_16_L_4_ surface. Encouragingly, the gamut encompassed successful modifications: chloroacetate (Cla^−^) and bromoacetate (Bra^−^) through path I, and acetate (Ac^−^),^47^ aminoacetate (Ama^−^), glycinate (Ga^−^),^48^ and oxalate (Oa^2−^)^49^*via* path II. A comprehensive SCXRD analysis discerned the occupation of all four sites by these carboxylates. Delving into specifics, the bridging modes of Ac^−^, Ama^−^, Cla^−^, and Bra^−^ ligands aligned uniformly, establishing μ_2_-(O,O′) bridges connecting two Ti sites (as depicted in Fig. 6A–D). In marked contrast, Oa^2−^ and Ga^−^ ligands exhibited an intriguingly different binding pattern (as showcased in Fig. 6E andF), with the ligands forming a chelating arrangement around the Ti3 site in a bidentate μ_1_-(O,O) mode, concomitantly coordinating the Ti2 site with two OH^−^ ions. Notably, the structural analysis of Ti_16_L_4–_1 modified with trifluoroacetate (Tfa^−^) encountered challenges due to poor crystal quality. This phenomenon can be attributed to the introduction of a significant number of F atoms to the cluster's surface, engendering repulsion among clusters within the tetragonal phase and leading to lattice displacement. However, this issue was effectively surmounted in the case of the Tfa^−^ modified Ti_16_L_4–_2 cluster, revealing a definitive structural determination within the hexagonal lattice of Ti_16_L_4–_2 (Fig. 6G), underscoring the adaptability of the Tfa^−^ to occupy these sites.

Interestingly, our explorations extended to the realm of inorganic oxyanions. When Ti_16_L_4–_1/NaOH crystals were immersed in 1 M H_3_PO_4_ for 30 minutes, red crystals of Ti_16_L_4–_1/H_3_PO_4_ were obtained, retaining crystallinity. SCXRD analysis brought forth an intriguing revelation—PO_4_^3−^ did not interact with the clusters. Instead, OH^−^ persisted as occupants within the 12 labile sites, with Na^+^ ions being entirely dislodged from the structure. Notably, the cumulative occupancy rate of the four μ_2_-OH^−^ sites reached 2.80 (Fig. S22†). This intriguing outcome underscores our capacity to effect partial ligand removal from these four μ_2_-sites through a meticulous interplay of two acid–base neutralization reactions. This multifaceted exploration, together with the systematic elucidation of intricate ligand exchange pathways, reflects the groundbreaking dimension of our research endeavor.

### CO_2_ photoreduction activities

**Fig. 7:**
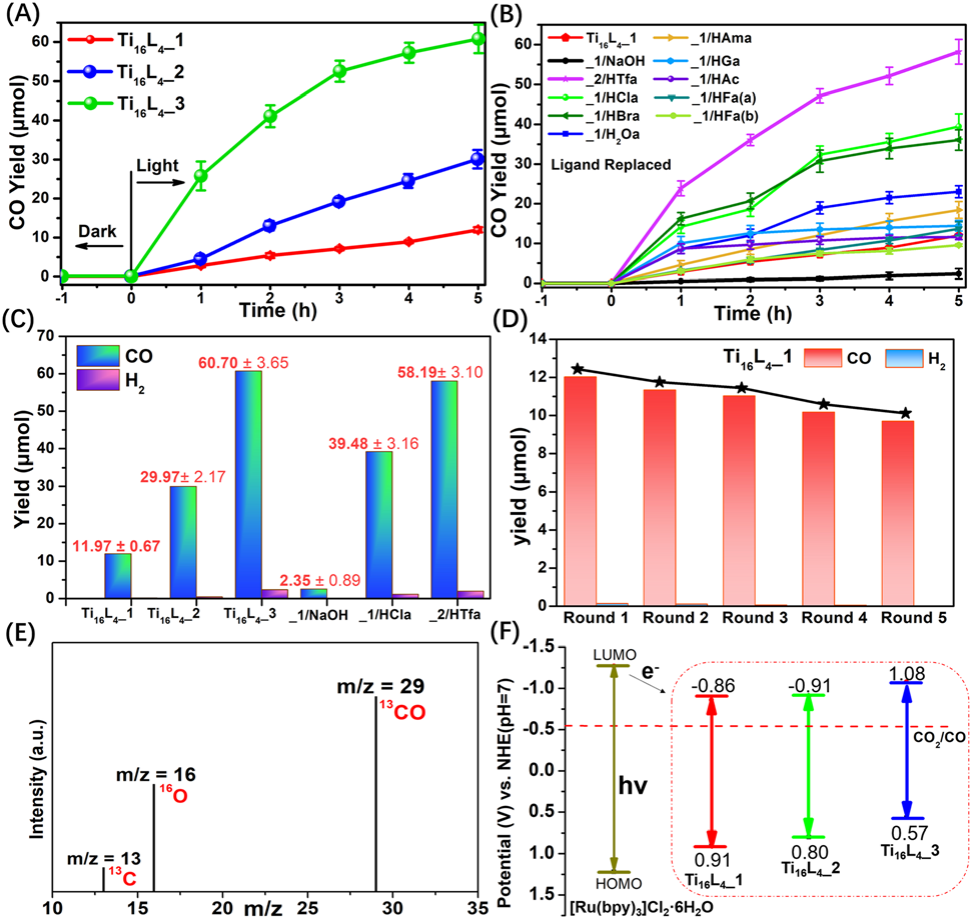
(A) Time-dependent CO generation profiles for Ti_16_L_4–_1/2/3; (B) time-dependent CO generation profiles for Ti_16_L_4_ with various ligand modifications; (C) CO production rates over Ti_16_L_4_; (D) durability assessments of Ti_16_L_4–_1. (E) ^13^CO recorded under ^13^CO_2_ atmosphere; (F) the energy band structure diagram for Ti_16_L_4_. Reaction conditions: 3 mg of Ti_16_L_4_, 10 mg [Ru(bpy)_3_]Cl_2_·6H_2_O, 5 mL triethanolamine (TEOA), acetonitrile (8 mL), H_2_O (2 mL), CO_2_ (1 atm), *λ* ≥ 420 nm, 25 °C, 5 h.

The promising avenue of photocatalytically reducing CO_2_ into reusable chemicals looms large as a critical step toward carbon neutrality.^50^ However, the reported TOCs for driving such reactions remain limited, evoking a pressing need for innovative solutions.^51–55^ Harnessing the unparalleled stability, controllable ligands, and adaptable packing modes inherent to Ti_16_L_4_, we ventured into their application as heterogeneous photocatalysts for CO_2_ reduction. Conceiving the intricate setup, CO_2_ photoreduction over Ti_16_L_4_ unfolded under visible light irradiation (*λ* ≥ 420 nm) in the presence of [Ru(bpy)_3_]Cl_2_·6H_2_O as a photosensitizer, triethanolamine (TEOA) as a sacrificial agent, and a CH_3_CN/H_2_O (4 : 1, v/v) solvent composition. A comprehensive analysis of the product, employing gas chromatography (GC, Fig. S47†), ion chromatography (IC, Fig. S51†) and ^1^H NMR (Fig. S52†), lucidly affirmed the exclusive generation of CO *via* CO_2_RR, with a marginal presence of H_2_. It's noteworthy that no products were observed in the absence of irradiation, CO_2_ or clusters, or the yields were exceptionally low (Table S4†). This unequivocally underscores the orchestrated interplay of each component within the photocatalytic system. Comparative assessment of the catalytic activity of Ti_16_L_4–_1/2/3 in CO_2_ photoreduction revealed a remarkable hierarchy in CO yields. Ti_16_L_4–_1 produced a modest CO yield of 11.97 ± 0.67 μmol within 5 hours, with Ti_16_L_4–_2 and Ti_16_L_4–_3 exhibiting substantially enhanced CO yields of 29.97 ± 2.17 μmol and 60.70 ± 3.65 μmol, respectively (Fig. 7A). Ti_16_L_4–_3 emerged as the frontrunner in catalytic prowess, displaying an impressive CO production rate of 4046.67 ± 243.33 μmol g^−1^ h^−1^, coupled with an exceptional selectivity of 96.28%. These activity and selectivity levels surpassed those of many cluster or MOF-based materials for CO_2_ to CO conversion.^56–61^ The catalytic proficiency of this macrocyclic series aligns with the potentially catalytically active Ti^IV^ sites boasting flexible coordination spaces (specifically the Ti2 and Ti3 sites), which stand poised for CO_2_ adsorption. In Ti_16_L_4–_3, Cl^−^ exhibit a greater tendency to vacate compared to acetates in Ti_16_L_4–_1. Consequently, the Ti sites in Ti_16_L_4–_3 can showcase higher catalytic activity. This assessment of catalytic activity, supported by Table S5,† revealed that each potential Ti^4+^ catalytic site (TON_Ti_) in Ti_16_L_4–_3 exhibited higher activity than those in Ti_16_L_4–_1/2. Additionally, the strategic advantage of the 2 nm macrochannel within Ti_16_L_4–_3 unveiled a larger surface area, fostering heightened CO_2_ adsorption and exposure to additional catalytic enclaves. Experimental findings in CO_2_ adsorption bolstered these revelations (Fig. S36†), where Ti_16_L_4–_3 showcased superior performance over Ti_16_L_4–_1/2, with CO_2_ uptake values of 57.38 and 48.79 cm^3^ g^−1^, respectively, at 273 K and 298 K under 1 bar pressure. Intriguingly, functionalization of ligands on Ti_16_L_4_ also had a notable influence on catalytic activity (Fig. 7B). In the case of Ti_16_L_4–_1/NaOH, all potential catalytic sites on the cluster's surface are occupied by OH^−^ ions. Due to the presence of strong Ti–OH bonds, CO_2_ is unable to access the catalytic Ti sites, resulting in a decrease in catalytic activity (2.35 ± 0.89 μmol for 5 hours). Noteworthy examples include Ti_16_L_4–_1/HBra and Ti_16_L_4–_1/HCla, unveiling CO yields of 36.25 ± 2.59 μmol and 39.48 ± 3.16 μmol, respectively, after 5 hours, approximately threefold higher than pristine Ti_16_L_4–_1 (Fig. 7C). Similarly, Ti_16_L_4–_2/HTfa exhibited double the CO production of its untouched counterpart (58.18 ± 3.10 μmol for 5 hours), Ti_16_L_4–_2. The higher photocatalytic performance of those derived clusters is mainly due to their modification with halogenated carboxylates that have the ability to quickly transfer charges. These enhancements find resonance in the modification-induced shifts within the cluster's bandgap structures, catalyzing accelerated electron transfers, and amplifying catalytic efficiency.

Collectively, these revelations converge to spotlight the photocatalytic acumen inherent within the Ti_16_L_4_ macrocycles, unfurling their supremacy in CO_2_ reduction and accentuating their proclivity for selectively generating CO. What's more, the strategic orchestration of the Ti_16_L_4_'s spatial configuration, coupled with tailored enhancements infused into its outer ligands, emerges as a potent avenue for catapulting its catalytic performance to new heights. A tangible testament to the robustness of these findings is the recyclability assessment, which resoundingly affirmed the enduring vigor of Ti_16_L_4–_1's photocatalytic activity even across five successive cycles (Fig. 7D). It's worth highlighting that Ti_16_L_4–_1 not only retained its remarkable photocatalytic stability but also withstood scrutiny through XPS, MALDI-TOF-MS and TEM morphology studies (Fig. S58–S60†). Subsequent SCXRD analysis of Ti_16_L_4–_1 post photocatalysis revealed subtle changes in the coordination environment of potential catalytic sites (Ti2 and Ti3) within the cluster, which further substantiates the catalytic activity of these flexible coordination sites (Fig. S57†). Following the culmination of five cycles, the catalyst could be efficiently reclaimed from the reaction milieu. A analysis of the resultant supernatant *via* Inductively-Coupled Plasma (ICP) revealed an astonishingly low Ti leakage rate from Ti_16_L_4–_1, accounting for a mere 0.01% of the total Ti content. This revelation underscores not only the catalyst's enduring stability but also its marked potential for pragmatic, real-world applications. To unravel the origin of the carbon in the generated CO, an isotopic experiment leveraging ^13^CO_2_ as the carbon source was conducted. The gas chromatography-mass spectrometry (GC-MS) identified the production of ^13^CO, confirming the unequivocal derivation of CO from CO_2_ (Fig. 7E).

The investigation into electron transfer in the catalytic process unveiled the flat band potentials of Ti_16_L_4–_1/2/3*via* Mott–Schottky plots, pinpointing their calculated conduction band minimum (CBM) values. These CBM values resided at −0.86, −0.91, and −1.08 V *vs.* the normal hydrogen electrode (NHE), respectively. Strikingly, all LUMO potentials exhibited a notably more negative profile in contrast to CO_2_/CO (−0.51 V *vs.* NHE), signifying their inherent suitability for CO_2_RR (Fig. 7F). Previous reports have indicated that the LUMO of [Ru(bpy)_3_]Cl_2_·6H_2_O is −1.27 V *vs.* NHE.^62^ Under irradiation, [Ru(bpy)_3_]Cl_2_·6H_2_O transits to an excited state, subsequently reductively quenched by TEOA, yielding a reducing photosensitizer. Given that the CBM values of Ti_16_L_4–_1/2/3 lie below the LUMO of [Ru(bpy)_3_]Cl_2_·6H_2_O, the excited electrons emanating from the reduced photosensitizer migrate to Ti_16_L_4_, setting off the activation of adsorbed CO_2_ on its surface. This orchestrated sequence culminates in the reduction of CO_2_ to CO, subsequently liberating the product. We further substantiated the photocatalytic mechanism through electron paramagnetic resonance (EPR) experiments (Fig. S53†). The experimental findings reveal that in the absence of light irradiation within an N_2_ atmosphere, the reaction system involving Ti_16_L_4–_1 and the sacrificial agent did not exhibit any ESR signals. Nevertheless, when subjected to visible light irradiation, distinct Ti^3+^ signals were observed, corresponding to *g* value of 1.945. This observation implies that the photoexcited electrons transfers to Ti^4+^, leading to their reduction to Ti^3+^. Concurrently, TEOA serves as the sacrificial agent to neutralize the photogenerated holes. The intensity of the Ti^3+^ signal gradually increased with extended irradiation time. Upon exposure of the reaction system to a CO_2_ atmosphere, the ESR signal of Ti^3+^ diminished, signifying the involvement of photogenerated Ti^3+^ in CO_2_RR. ESR results affirm that Ti^4+^ within Ti_16_L_4–_1 function as the active centers for photocatalytic CO_2_RR, providing further support for this mechanism.

**Fig. 8:**
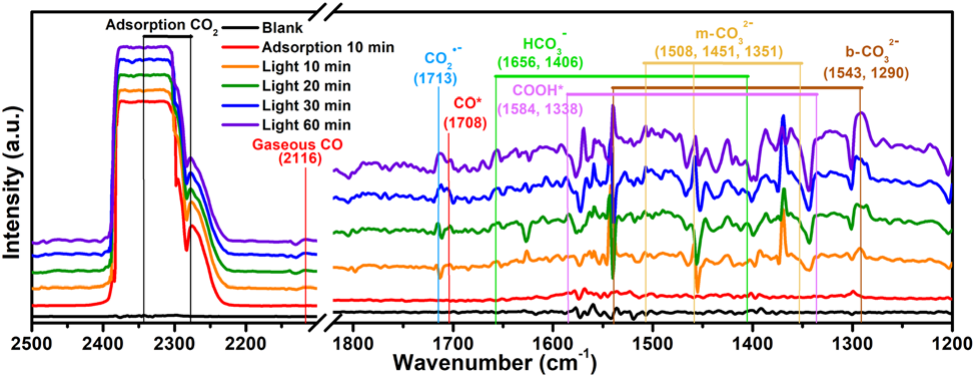
*In situ* FTIR spectra on Ti_16_L_4–_1.

To probe the CO_2_ radical and other reaction intermediates in the photocatalytic reaction, the Ti_16_L_4–_1 is investigated by *in situ* diffuse reflectance infrared Fourier transform spectroscopy (Fig. 8). Under dark conditions post CO_2_ pretreatment, Ti_16_L_4–_1 displayed prominent peaks around 2348 cm^−1^, which is associated with the asymmetric stretching of absorbed CO_2_.^59^ Exposure to a CO_2_ atmosphere under light for 10 minutes resulted in several new peaks: monodentate carbonate (m-CO_3_^2−^) at 1351, 1451, and 1508 cm^−1^; bidentate carbonate (b-CO_3_^2−^) at 1290 and 1543 cm^−1^; and bicarbonate (HCO_3_^−^) at 1406 and 1656 cm^−1^. These carbonates and bicarbonates likely formed from CO_2_ reacting with water vapor. Notably, the CO_2_˙^−^ signal at 1713 cm^−1^ intensified with prolonged irradiation, indicating the formation of the CO_2_ radical, a key intermediate in CO_2_ photoreduction to *COOH.^52,53^ Furthermore, *COOH groups, crucial in CO_2_ reduction to CO, were identified at 1338 and 1584 cm^−1^, with increasing peak intensities under light exposure, suggesting light-induced formation.^63^ Additionally, absorption peaks for *CO and gaseous CO at 1708 and 2116 cm^−1^ respectively, provided further evidence of the reaction pathway.^64^ Therefore, according to the above analysis, a rational CO_2_ photoreduction mechanism was proposed (Fig. S54†): CO_2_ was initially adsorbed on the Ti^3+^. Subsequently, the adsorbed *CO_2_ received electrons from Ti^3+^ and with protons to form the *COOH during irradiation. Thereafter, the deprotonation of the *COOH intermediate further generation of CO, and ultimately desorbs to obtain CO molecules.

## Conclusions

In summary, this research marks a pioneering exploration into the realm of Ti^IV^-based metallamacrocycles, unveiling a host of novel insights and underlining its significance in the broader landscape of scientific inquiry. Foremost, the unparalleled stability demonstrated by the Ti_16_L_4_ stands out as a groundbreaking revelation. Its exceptional resilience against a spectrum of challenges, encompassing organic solvents, concentrated acids, and alkali, attests to a robustness that guarantees structural integrity and endurance across diverse environmental contexts. This stability not only solidifies the cluster's foundation but also paves the way for its application across a multitude of conditions. Equally groundbreaking is the abundance of coordination anchors inherent on the Ti_16_L_4_'s surface, effectively serving as a fertile ground for subsequent ligand adaptations. The dual pathways for facile ligand exchange form the bedrock of a versatile platform, facilitating the creation of microporous materials endowed with a plethora of surface functionalities. These microporous materials with coordinating sites on their surfaces are promising as carriers for loading noble metal nanoparticles, constructing heterogeneous catalysts with highly efficient catalytic activity. Another revelation lies in the crystallization potential of the macrocycle, giving rise to three distinct phases contingent upon the employed surface ligands. This revelation casts a revelatory spotlight on the predominant forces orchestrating the formation of diverse cluster-packing modes within the crystal lattice. The capability to manipulate surface functionalities and packing arrangements not only broadens our fundamental understanding but also unlocks a trove of opportunities for finely honing chemical and physical attributes to align with specific applications.

## Data availability

The data that support the findings of this study are available in the main text and the ESI.†

## Author contributions

C. L. supervised the project and conceived the idea. Y. Q. T. and L. F. D carried out synthesis, characterization and photocatalytic experiment of clusters. C. L. and Y. Q. T. wrote the manuscript. All authors discussed the experimental results.

## Conflicts of interest

There are no conflicts of interest to declare.

## Supplementary Material

SC-014-D3SC06046B-s001

SC-014-D3SC06046B-s002

## References

[cit1] Dahl M., Liu Y. D., Yin Y. D. (2014). Chem. Rev..

[cit2] Ma Y., Wang X. L., Jia Y. S., Chen X. B., Han H. X., Li C. (2014). Chem. Rev..

[cit3] Chen X., Liu L., Yu P. Y., Mao S. S. (2011). Science.

[cit4] Coppens P., Chen Y., Trzop E. (2014). Chem. Rev..

[cit5] Rozes L., Sanchez C. (2011). Chem. Soc. Rev..

[cit6] Matthews P. D., King T. C., Wright D. S. (2014). Chem. Commun..

[cit7] Benedict J. B., Freindorf R., Trzop E., Cogswell J., Coppens P. (2010). J. Am. Chem. Soc..

[cit8] Fang W. H., Zhang L., Zhang J. (2018). Chem. Soc. Rev..

[cit9] Zhu Q. Y., Dai J. (2021). Coord. Chem. Rev..

[cit10] Yuan S., Qin J. S., Xu H. Q., Su J., Rossi D., Chen Y. P., Zhang L. L., Lollar C., Wang Q., Jiang H. L., Son D. H., Xu H. Y., Huang Z. H., Zou X. D., Zhou H. C. (2018). ACS Cent. Sci..

[cit11] Sokolow J. D., Trzop E., Chen Y., Tang J., Allen L. J., Crabtree R. H., Benedict J. B., Coppens P. (2012). J. Am. Chem. Soc..

[cit12] Zhang G., Liu C., Long D.-L., Cronin L., Tung C. H., Wang Y. (2016). J. Am. Chem. Soc..

[cit13] Guo Y., Hou J. L., Luo W., Li Z. Q., Zou D. H., Zhu Q. Y., Dai J. (2017). J. Mater. Chem. A.

[cit14] Zou D. H., Cui L. N., Liu P. Y., Yang S., Zhu Q. Y., Dai J. (2019). Inorg. Chem..

[cit15] Lv Y., Cheng J., Steiner A., Gan L., Wright D. S. (2014). Angew. Chem., Int. Ed..

[cit16] Zhang L., Fan X., Yi X. F., Lin X., Zhang J. (2022). Acc. Chem. Res..

[cit17] Geng L., Liu C. H., Wang S. T., Fang W.-H., Zhang J. (2021). Angew. Chem., Int. Ed..

[cit18] Gao M.-Y., Wang F., Gu Z. G., Zhang D. X., Zhang L., Zhang J. (2016). J. Am. Chem. Soc..

[cit19] Fan X., Wang J., Wu K., Zhang L., Zhang J. (2019). Angew. Chem., Int. Ed..

[cit20] Fang W. H., Zhang L., Zhang J. (2016). J. Am. Chem. Soc..

[cit21] Zhang G., Li W., Liu C., Jia J., Tung C. H., Wang Y. (2018). J. Am. Chem. Soc..

[cit22] Liu J. X., Gao M. Y., Fang W. H., Zhang L., Zhang J. (2016). Angew. Chem., Int. Ed..

[cit23] Geng L., Liu C. H., Wang S. T., Fang W. H., Zhang J. (2020). Angew. Chem., Int. Ed..

[cit24] Tian Y. Q., Cui Y. S., Yu W. D., Xu C. Q., Yi X. Y., Yan J., Li J., Liu C. (2022). Chem. Commun..

[cit25] Tian Y. Q., Cui Y. S., Zhu J. H., Xu C. Q., Yi X. Y., Li J., Liu C. (2022). Chem. Commun..

[cit26] Zhou S. Y., Li C. P., Fu H., Cao J., Zhang J., Zhang L. (2020). Chem.–Eur. J..

[cit27] Liu M., Liao W. P., Hu C., Du S. C., Zhang H. J. (2012). Angew. Chem., Int. Ed..

[cit28] Pei W. Y., Xu G., Yang J., Wu H., Chen B., Zhou W., Ma J. F. (2017). J. Am. Chem. Soc..

[cit29] Han H. T., Kan L., Li P., Zhang G. S., Li K. Y., Liao W. P., Liu Y. L., Chen W., Hu C. H. T. (2021). Sci. China: Chem..

[cit30] Hang X. X., Liu B., Zhu X. F., Wang S. T., Han H. T., Liao W. P., Liu Y. L., Hu C. H. (2016). J. Am. Chem. Soc..

[cit31] Wang S., Gao X., Hang X., Zhu X., Han H., Li X., Liao W., Chen W. (2018). J. Am. Chem. Soc..

[cit32] Wang Z., Su H. F., Gong Y. W., Qu Q. P., Bi Y. F., Tung C. H., Sun D., Zheng L. S. (2020). Nat. Commun..

[cit33] Hou B., Zheng H. Y., Zhang K. H., Wu Q., Qin C., Sun C. Y., Pan Q. H., Kang Z. H., Wang X. L., Su Z. M. (2023). Chem. Sci..

[cit34] Tasiopoulos A. J., Vinslava A., Wernsdorfer W., Abboud K. A., Christou G. (2004). Angew. Chem., Int. Ed..

[cit35] Kitagawa H., Ohtsu H., Kawano M. (2013). Angew. Chem., Int. Ed..

[cit36] Zhang Z. M., Li Y. G., Yao S., Wang E. B., Wang Y. H., Clerac R. (2009). Angew. Chem., Int. Ed..

[cit37] Scullion R. A., Surman A. J., Xu F., Mathieson J. S., Long D. L., Haso F., Liu T., Cronin L. (2014). Angew. Chem., Int. Ed..

[cit38] Zhao C., Han Y.-Z., Dai S., Chen X., Yan J., Zhang W., Su H., Lin S., Tang Z., Teo B. K., Zheng N. (2017). Angew. Chem., Int. Ed..

[cit39] Øien-Ødegaard S., Bazioti C., Redekop E. A., Prytz O., Lillerud K. P., Olsbye U. (2020). Angew. Chem., Int. Ed..

[cit40] Zheng X. Y., Jiang Y. H., Zhuang G. L., Liu D. P., Liao H. G., Kong X. J., Long L. S., Zheng L. S. (2017). J. Am. Chem. Soc..

[cit41] Tian Y. Q. , Dai L. F., Mu W. L., Yu W. D., Yan J. and Liu C., CCDC 2174429: Experimental Crystal Structure Determination, 10.5517/ccdc.csd.cc2bznww

[cit42] Tian Y. Q. , Dai L. F., Mu W. L., Yu W. D., Yan J. and Liu C., CCDC 2174430: Experimental Crystal Structure Determination, 10.5517/ccdc.csd.cc2bznxx

[cit43] Tian Y. Q. , Dai L. F., Mu W. L., Yu W. D., Yan J. and Liu C., CCDC 2174431: Experimental Crystal Structure Determination, 10.5517/ccdc.csd.cc2bznyy

[cit44] Tian Y. Q. , Dai L. F., Mu W. L., Yu W. D., Yan J. and Liu C., CCDC 2174432: Experimental Crystal Structure Determination, 10.5517/ccdc.csd.cc2bznzz

[cit45] Tian Y. Q. , Dai L. F., Mu W. L., Yu W. D., Yan J. and Liu C., CCDC 2174433: Experimental Crystal Structure Determination, 10.5517/ccdc.csd.cc2bzp01

[cit46] Tian Y. Q. , Dai L. F., Mu W. L., Yu W. D., Yan J. and Liu C., CCDC 2174435: Experimental Crystal Structure Determination, 10.5517/ccdc.csd.cc2bzp23

[cit47] Tian Y. Q. , Dai L. F., Mu W. L., Yu W. D., Yan J. and Liu C., CCDC 2174434: Experimental Crystal Structure Determination, 10.5517/ccdc.csd.cc2bzp12

[cit48] Tian Y. Q. , Dai L. F., Mu W. L., Yu W. D., Yan J. and Liu C., CCDC 2174437: Experimental Crystal Structure Determination, 10.5517/ccdc.csd.cc2bzp45

[cit49] Tian Y. Q. , Dai L. F., Mu W. L., Yu W. D., Yan J. and Liu C., CCDC 2174438: Experimental Crystal Structure Determination, 10.5517/ccdc.csd.cc2bzp56

[cit50] Zhou J., Li J., Kan L., Zhang L., Huang Q., Yan Y., Chen Y., Liu J., Li S. L., Lan Y. Q. (2022). Nat. Commun..

[cit51] Li N., Liu J.-J., Sun J.-W., Dong B.-X., Dong L.-Z., Yao S.-J., Xin Z. F., Li S.-L., Lan Y.-Q. (2020). Green Chem..

[cit52] Li N., Lin J.-M., Li R.-H., Shi J.-W., Dong L.-Z., Liu J., He J., Lan Y.-Q. (2023). J. Am. Chem. Soc..

[cit53] Liu J.-J., Li N., Sun J.-W., Liu J., Dong L.-Z., Yao S.-J., Zhang L., Xin Z.-F., Shi J.-W., Wang J.-X., Li S.-L., Lan Y.-Q. (2021). ACS Catal..

[cit54] Li N., Liu J., Liu J. J., Dong L. Z., Li S. L., Dong B. X., Kan Y. H., Lan Y. Q. (2019). Angew. Chem., Int. Ed..

[cit55] Fu Y., Sun D., Chen Y., Huang R., Ding Z., Fu X., Li Z. (2012). Angew. Chem., Int. Ed..

[cit56] Li R., Hu J. H., Deng M. S., Wang H. L., Wang X. J., Hu Y. L., Jiang H. L., Jiang J., Zhang Q., Xie Y., Xiong Y. J. (2014). Adv. Mater..

[cit57] Wang S. B., Yao W. S., Lin J. L., Ding Z. X., Wang X. C. (2014). Angew. Chem., Int. Ed..

[cit58] Zhao J., Wang Q., Sun C. Y., Zheng T. T., Yan L. K., Li M. T., Shao K. Z., Wang X. L., Su Z. M. (2017). J. Mater. Chem. A.

[cit59] Zhang H.-X., Hong Q.-L., Li J., Wang F., Huang X. S., Chen S. M., Tu W. G., Yu D. S., Xu R., Zhou T. H., Zhang J. (2019). Angew. Chem., Int. Ed..

[cit60] Li X. X., Zhang L., Yuan L., Wang T., Dong L. Z., Huang K., Liu J., Lan Y. Q. (2022). Chem. Eng. J..

[cit61] Du J., Ma Y., Xin X., Na H., Zhao Y., Tan H., Han Z., Li Y., Kang Z. (2020). Chem. Eng. J..

[cit62] Bag P. P., Wang X. S., Sahoo P., Xiong J. H., Cao R. (2017). Catal. Sci. Technol..

[cit63] Fan G., Li R. X., Yang S. Z., Zhang X. Y., Jian H. Y., Urban J. J., Sun W.-Y. (2023). Angew. Chem., Int. Ed..

[cit64] Dong J.-P., Xu Y., Zhang X.-G., Zhang H., Yao L., Wang R., Zang S.-Q. (2023). Angew. Chem., Int. Ed..

