# Supplementary material for: Atomically accurate site-specific ligand tailoring of highly acid- and alkali-resistant Ti(iv)-based metallamacrocycle for enhanced CO2 photoreduction
Source: Chem Sci. 2023 Nov 30;14(48):14280–9. doi: 10.1039/d3sc06046b (PMC10718071; doi:10.1039/d3sc06046b)
Supplement: SC-014-D3SC06046B-s001 [file SC-014-D3SC06046B-s001.pdf]

Electronic Supplementary Material (ESI) for Chemical Science.
This journal is © The Royal Society of Chemistry 2023

Supporting Information

Atomically Accurate Site-Specific Ligand Tailoring of Highly Acid- and Alkali-Resistant Ti(IV)-based Metallamacrocycle for Enhanced CO_2 Photoreduction

Yi-Qi Tian,[a‡] Lin-Fang Dai,[a‡] Wen-Lei Mu,[a] Wei-Dong Yu,[b] Jun Yan,[a*] Chao Liu[a*]

[a]Hunan Provincial Key Laboratory of Chemical Power Sources, College of Chemistry and Chemical Engineering, Central South University, Changsha 410083, Hunan, P. R. China. Correspondence and requests for materials should be addressed to C.L.

[b]China College of Science, Hunan University of Technology and Business, Changsha 410000, P. R. China.

Abstract: Skillfully engineering surface ligands at specific sites within robust clusters presents both a formidable challenge and a captivating opportunity. Herein we unveil an unprecedented titanium-oxo cluster: a calix[8]arene-stabilized metallamacrocycle ($Ti_{16}L_4$), uniquely crafted through the fusion of four "core–shell" {Ti_4@(TBC[8])(L)} subunits with four oxalate moieties. Notably, this cluster showcases an exceptional level of chemical stability, retaining its crystalline integrity even when immersed in highly concentrated acid (1M HNO_3) and alkali (20M NaOH). The macrocycle's surface unveils four specific, customizable μ_2-bridging sites, primed to accommodate diverse carboxylate ligands. This adaptability is highlighted through deliberate modifications achieved by alternating crystal soaking in alkali and carboxylic acid solutions. Furthermore, $Ti_{16}L_4$ macrocycles autonomously self-assemble into one-dimensional nanotubes, which subsequently organize into three distinct solid phases, contingent upon the specific nature of the four μ_2-bridging ligands. Notably, the $Ti_{16}L_4$ exhibit a remarkable capacity for photocatalytic activity in selectively reducing CO_2 to CO. Exploiting the macrocycle's modifiable shell yields a significant boost in performance, achieving an exceptional maximum CO release rate of 4.047 ± 0.243 mmol g^{-1} h^{-1}. This study serves as a striking testament to the latent potential of precision-guided surface ligand manipulation within robust clusters, while also underpinning a platform for producing microporous materials endowed with a myriad of surface functionalities.

Contents

1. Experimental section 1
2. Detailed Structure Information for $Ti_{16}L_4$ compounds. 7
3. Powder X-ray diffraction 16
4. XPS Measurement 19
5. TG-Measurement 20
6. IR spectra 21
7. Energy Dispersive X-ray (EDX) Spectroscopic Analysis 22
8. CO_2 adsorption 23
9. Dynamic Light Scattering Measurement 23
10. Wettability of $Ti_{16}L_4$ rings 24
11. Photocatalysis 24

1. Experimental section

Materials and Characterization. All reagents were purchased commercially and were not further purified when used. Powder X-ray diffraction (PXRD) analysis were performed on a Rigaku Mini Flex II diffractometer at a 2θ range of 5–50° (5° min^{-1}) with CuKα radiation (λ =1.54056 Å). The solid-state UV/Vis spectra data of the cluster samples were obtained on UV-4000 spectrophotometer. The diameters of cluster were measured by DLS using a Zetasizer Nano ZS90 (Malvern Instruments, UK). The measurements were conducted at a scattering angle of 90° at 25 °C. The measurements were repeated thrice for each sample.

Synthesis for $[H_4Ti_{16}O_8(TBC[8])_4(C_2O_4)_4(Ac)_4(O^iPr)_8]\cdot{}^iPrOH\cdot13CH_3CN$ (**$Ti_{16}L_{4}$–1**)**:** TBC[8] (28 mg, 0.021 mmol), Oxalic acid (27mg，0.3 mmol), $Eu(Ac)_3$ (33.4mg，0.1mmol), and CH_3CN (5 mL) were added into a 25 mL Teflon lined stainless steel autoclave, and then added with $Ti(O^iPr)_4$ (200 μL, 0.65 mmol). The solution was sonicated for 5 min, then transferred to an oven at 120 °C for 3 days. After it cool to room temperature, red stripe crystals of **$Ti_{16}L_{4}$–1** were obtained. yield: 40% based on $Ti(O^iPr)_4$. The crystals are rinsed with CH_3CN and preserved under a sealed and dry environment. Elemental analysis calcd. (%) for $C_{423}H_{529}N_{14}O_{73}Ti_{16}$: C 65.60, H 6.88; found: C 64.63, H 6.57.

Synthesis for $[H_4Ti_{16}O_8(TBC[8])_4(C_2O_4)_4(Ac)_2Cl_2(O^iPr)_8]\cdot56CH_3CN$ (**$Ti_{16}L_{4}$–2**)**:** TBC[8](28 mg, 0.021 mmol), Oxalic acid (27mg, 0.3 mmol), $EuCl_3$(25.8mg, 0.1mmol), $Eu(Ac)_3$(15mg, 0.045mmol) and CH_3CN (5 mL) were added into a 25 mL Teflon lined stainless steel autoclave, and then added with $Ti(O^iPr)_4$ (200 μL, 0.65 mmol). The solution was sonicated for 5 min, then transferred to an oven at 120 °C for 3 days. After it cool to room temperature, red stripe crystals of **$Ti_{16}L_{4}$–2** were obtained. yield: 32% based on $Ti(O^iPr)_4$. The crystals are rinsed with CH_3CN and preserved under a sealed and dry environment. Elemental analysis calcd. (%) for $C_{506}H_{648}Cl_2N_{59}O_{68}Ti_{16}$: C 64.09, H 6.89; found: C 63.29, H 6.51.

Synthesis for $[H_4Ti_{16}O_8(TBC[8])_4(C_2O_4)_4Cl_4(O^iPr)_8]\cdot24CH_3CN$ (**$Ti_{16}L_{4}$–3**)**:** TBC[8] (28 mg，0.021 mmol), Oxalic acid (27mg，0.3 mmol), $EuCl_3$(25.8mg，0.1mmol) and CH_3CN (5 mL) were added into a 25 mL Teflon lined stainless steel autoclave, and

then added with $Ti(O^iPr)_4$ (200 µL, 0.65 mmol). The solution was sonicated for 5 min, then transferred to an oven at 120 °C for 3 days. After it cool to room temperature, red stripe crystals of **$Ti_{16}L_4$–3** were obtained. yield: 12% based on $Ti(O^iPr)_4$. The crystals are rinsed with CH_3CN and preserved under a sealed and dry environment. Elemental analysis calcd. (%) for $C_{432}H_{536}Cl_4N_{24}O_{64}Ti_{16}$: C 64.88, H 6.76; found: C 64.13, H 6.24.

Synthesis for $[Ti_{16}O_8(TBC[8])_4(C_2O_4)_4(Ac)_4(O^iPr)_4(H_2O)_4]·23H_2O$ (**$Ti_{16}L_4$–1**(a)): 20 mg crystal of **$Ti_{16}L_4$–1** was immersed in 1M formic acid solution. After soaking for 24 hours, the red crystals of **$Ti_{16}L_4$–1**(a) were collected by filtration and washed with deionized water for three times.

Synthesis for $[Ti_{16}O_8(TBC[8])_4(C_2O_4)_4(Fa)_4(O^iPr)_4(H_2O)_4]·3CH_3CN·24H_2O$ (**$Ti_{16}L_4$–1**/HFa(a)): 20 mg crystal of **$Ti_{16}L_4$–1** was immersed in 5M formic acid solution. After soaking for 24 hours, the red crystals of **$Ti_{16}L_4$–1**/HFa(a) were collected by filtration and washed with deionized water for three times.

Synthesis for $[Na_8Ti_{16}O_8(TBC[8])_4(C_2O_4)_4(OH)_{16}(H_2O)_{12}(CH_3CN)]·2CH_3CN·55H_2O$ (**$Ti_{16}L_4$–1**/NaOH)**:** 20 mg crystal of **$Ti_{16}L_4$–1** was immersed in 5M NaOH. The color of the crystal gradually changes from red to yellow. After soaking for 30min, the yellow crystals of **$Ti_{16}L_4$–1**/NaOH were collected by filtration and washed with deionized water for three times.

Synthesis for $[Na_4Ti_{16}O_8(TBC[8])_4(C_2O_4)_4(Fa)_4(OH)_8(H_2O)_4]·34H_2O$ (**$Ti_{16}L_4$–1**/HFa(b))**:** 20mg crystal of **$Ti_{16}L_4$–1**/NaOH was immersed in 1M formic acid solution. The color of the crystal gradually changes from yellow to red. After soaking for 30min, the yellow crystals of **$Ti_{16}L_4$–1**/HFa(b) were collected by filtration and washed with deionized water for three times.

Synthesis for $[Ti_{16}O_8(TBC[8])_4(C_2O_4)_4(Cla)_4(O^iPr)_4(H_2O)_4]·38H_2O$ (**$Ti_{16}L_4$–1**/HCla)**:** 20mg crystal of **$Ti_{16}L_4$–1** was immersed in 5M chloroacetic acid solution. After soaking for 24 hours, the red crystals of **$Ti_{16}L_4$–1**/HCla were collected by filtration and washed with deionized water for three times.

Synthesis for $[Ti_{16}O_8(TBC[8])_4(C_2O_4)_4(Bra)_4(O^iPr)_4(H_2O)_4]·38H_2O$ (**$Ti_{16}L_4$–1**/HBra)**:** 20mg crystal of **$Ti_{16}L_4$–1** was immersed in 5M trifluoroacetic acid solution.

After soaking for 24 hours, the red crystals of **$Ti_{16}L_{4}$–1**/HBra were collected by filtration and washed with deionized water for three times.

Synthesis for $[Na_2Ti_{16}O_8(TBC[8])_4(C_2O_4)_4(Ac)_{10}(HAc)_6]\cdot 2HAc\cdot 49H_2O$ (**$Ti_{16}L_{4}$–1**/HAc)**:** 20mg crystal of **$Ti_{16}L_{4}$–1**/NaOH was immersed in 1M acetic acid solution, and the crystal color gradually changes from red to yellow. After soaking for 30min, the red crystals of **$Ti_{16}L_{4}$–1**/HAc were collected by filtration and washed with deionized water for three times.

Synthesis for $[Na_4Ti_{16}O_8(TBC[8])_4(C_2O_4)_4(Ama)_8(H_2O)_{12}]$ (**$Ti_{16}L_{4}$–1**/HAma)**:** 20mg crystal of **$Ti_{16}L_{4}$–1**/NaOH was immersed in 1M aminoacetic acid solution, and the crystal color gradually changed from yellow to red after 30 min. The red crystals of **$Ti_{16}L_{4}$–1**/HAma were collected by filtration and washed with deionized water for three times.

Synthesis for $[Ti_{16}O_8(TBC[8])_4(C_2O_4)_4(Oa)_4(OH)_4(H_2O)_4]\cdot 49H_2O$ (**$Ti_{16}L_{4}$–1**/H_2Oa)**:** 20mg crystal of **$Ti_{16}L_{4}$–1**/NaOH was immersed in 1M oxalic acid solution, and the crystal color gradually changed from yellow to red. After soaking for 30min, the red crystals of **$Ti_{16}L_{4}$–1**/H_2Oa were collected by filtration and washed with deionized water for three times.

Synthesis for $[Ti_{16}O_8(TBC[8])_4(C_2O_4)_4(Ga)_4(OH)_4(H_2O)_4]\cdot 2CH_3CN\cdot 45H_2O$ (**$Ti_{16}L_{4}$–1**/HGa)**:** 20mg crystal of **$Ti_{16}L_{4}$–1**/NaOH was immersed in 1M glycolic acid solution, and the crystal color changed from yellow to red. After soaking for 30min, the red crystals of **$Ti_{16}L_{4}$–1**/HGa were collected by filtration and washed with deionized water for three times.

Synthesis for $[Ti_{16}O_8(TBC[8])_4(C_2O_4)_4(Tfa)_4(O^iPr)_4(H_2O)_4]\cdot 38H_2O$ (**$Ti_{16}L_{4}$–12**/HTfa)**:** 20mg crystal of **$Ti_{16}L_{4}$–2** was immersed in 5M trifluoroacetic acid solution. After soaking for 24 hours, the red crystals of **$Ti_{16}L_{4}$–1**/HTfa were collected by filtration and washed with deionized water for three times.

Synthesis for $[Ti_{16}O_8(TBC[8])_4(C_2O_4)_4(OH)_8(H_2O)_4]\cdot 4H_2O$ (**$Ti_{16}L_{4}$–1**/H_3PO_4)**:** 20mg crystal of **$Ti_{16}L_{4}$–1**/NaOH was immersed in 1M phosphoric acid solution, and the crystal color changed from yellow to red. After soaking for 30min, the red crystals of

$Ti_{16}L_{4}$–1/OH^- were collected by filtration and washed with deionized water for three times.

X-ray Crystallographic Analyses. Crystallographic data of all the crystals were collected on Bruker D8 Venture diffractometer with liquid metal Ga-Kα radiation (λ = 1.3405 Å) at about 100 K. Absorption correction was applied using SADABS.[1] The structures were solved with the dual-direct methods using ShelxT and refined with the full-matrix least-squares technique based on F^2 using the SHELXL-2014.[2, 3] Non-hydrogen atoms were refined anisotropically. Due to the rotation disorder of tert-butyl groups, in all cases the ISOR, DELU and SIMU constraints were necessary to achieve convergence. Hydrogen atoms were added theoretically, riding on the concerned atoms and refined with fixed thermal factors. Hydrogen atoms bonded to oxygen (water and hydroxyl ions) were not located from difference maps or included in the refinement. Besides, some water molecule is severely disordered and the related hydrogen atoms were not included. For all the clusters, the highly disordered solvent molecules in the crystal structures were removed with the SQUEEZE program in PLATON. All absorption corrections were performed using the multiscan program. The obtained crystallographic data for these cluster are summarized in Table S1 to Table S2.

Reference：

1. Sheldrick, G. M. SADABS: Program for Area Detector Adsorption Correction; University of Göttingen: Göttingen, Germany, 1996.
2. Sheldrick, G. M. SHELXL-2014/7: A Program for Structure Refinement; University of Göttingen: Göttingen, Germany, 2014.
3. Dolomanov, O. V.; Bourhis, L. J.; Gildea, R. J. J.; Howard, A. K.; Puschmann, H. OLEX^2: a complete structure solution, refinement and analysis program. J. Appl. Crystallogr. 2009, 42, 339−341.

Preparation of active samples for gas adsorption Crystals were soaked in ethanol and heated at 80 ^{o}C for three days. During this time, the ethanol solution should be exchanged several times a day. Subsequently, the sample was degassed under a dynamic vacuum at 100 ^{o}C for 6 h to activate the sample. The gas adsorption isotherms of active samples were obtained on a Micromeritics ASAP 2020 volumetric adsorption instrument.

Photoelectrochemical measurements were performed on a CHI 660e electrochemical workstation in a standard three-electrode electrochemical cell with a working electrode,

a platinum plate as the counter electrode, and a saturated calomel electrode as the reference electrode. These three electrodes were immersed in the 0.2M Na_2SO_4 aqueous solution (pH = 6.6). A 300 W high-pressure xenon lamp with UV cut-off filter was used as a full-wavelength light source, located 20 cm away from the working electrode. The colloidal dispersion was obtained by ultrasonic treatment of 5 mg ground crystal sample in 1 ml ethanol for 30 min, and then the dispersion was dropped onto FTO glass (0.75 cm^2 area). After evaporation under an ambient atmosphere for 2 h, the coating film was obtained and used as the working electrode. Mott-Schottky plots were recorded at frequencies of 600, 800, and 1000 Hz, respectively.

Contact Angle Measurements. Contact angles were measured on powder samples using a contact angle meter with a rotatable substrate holder. To perform contact angle measurements, 20 mg of powder samples of the clusters were deposited on a glass substrate bed. Then, powders were pressed to make a flat surface by the glass slide. A 10 μL water droplet was released slowly on the flat surface of the powder samples. The droplet image was taken by a high-performance charge-coupled device (CCD) sensor. The contact angle of all powder samples was analyzed by five-point simulation analysis.

Photocatalytic experiments. The photocatalytic experiments were performed in a 100 mL optical reaction vessel with stirring at ambient temperature. A mixture of 3 mg photocatalyst, 10 mg $[Ru(bpy)_3]Cl_2{\cdot}6H_2O$ and 5 mL triethanolamine (TEOA) was put into optical reaction vessel, followed by the addition of 8 mL acetonitrile and 2 mL deionized water. After homogeneous mixing, the suspension was stirred and purged with carbon dioxide for 10 min to remove air. A 300 W Xe lamp equipped with a UV cut-off filter (λ ≥ 420 nm) was utilized as the light source. For each evaluation of gaseous products, 400 μL of the headspace was injected into the gas chromatography (Shimadzu GC2014, argon as a carrier gas) every hour.

In situ diffuse reflectance infrared Fourier transform spectroscopy (DRIFTS) measurements. In situ DRIFTS over **$Ti_{16}L_4$_1** was obtained by using a Bruker INVENIO S FT-IR spectrophotometer, equipped with an MCT detector cooled by liquid nitrogen and a commercial reaction chamber from Harrick Scientific. The sample

was degassed at 373 K in N_2 atmosphere for 30 min, and then the gas flow was switched to pure CO_2 through deionized water for adsorption at room temperature. The sample was irradiated by the Xe lamp through a quartz window. The gaseous mixture of CO2 and H_2O vapor were steadily produced before the measurement. The background spectrum was collected after 10 min of adsorption in pure CO_2. After then, the IR spectra were collected in situ through the MCT detector. Each spectrum was recorded by averaging 64 scans at a 4 cm^{-1} spectral resolution.

2. Detailed Structure Information for $Ti_{16}L_4$ Compounds.

Table S1: Crystallographic data of the three $Ti_{16}L_4$ compounds.

Compounds	**$Ti_{16}L_4$_1**	**$Ti_{16}L_4$_2**	**$Ti_{16}L_4$_3**
CCDC	2174429	2174430	2174431
Cryst. syst.	monoclinic	monoclinic	tetragonal
Space group	C2/c	C2/c	I422
Formula	$C_{423}H_{529}N_{14}O_{73}Ti_{16}$	$C_{396}H_{484}Cl_2N_4O_{68}Ti_{16}$	$C_{432}H_{536}Cl_4N_{24}O_{64}Ti_{16}$
Fw	7743.97	7225.14	7997.02
a[Å]	39.1900(19)	53.303(2)	38.021(13)
b[Å]	32.5046(16)	19.9467(8)	38.021(13)
c[Å]	38.1646(18)	50.645(2)	19.702(4)
β[°]	115.461(2)	121.288(2)	90
V[Å^3]	43894(4)	46016(3)	28481(20)
Z	4	4	2
ρ_{calc}g/cm^3	1.079	1.067	0.933
μ/mm^{-1}	1.921	1.818	1.618
F(000)	15216.0	15623.0	8456.0
Data/restraints/parameters	38766/325/2344	40607/275/2453	10299/535/648
Goof	1.105	1.031	1.009
R_1/wR_2(I> 2σ(I))	0.0997/0.2961	0.0950/0.2783	0.0622/0.1693
R_1/wR_2(all data)	0.1166/0.3132	0.1070/0.2930	0.0888/0.1865

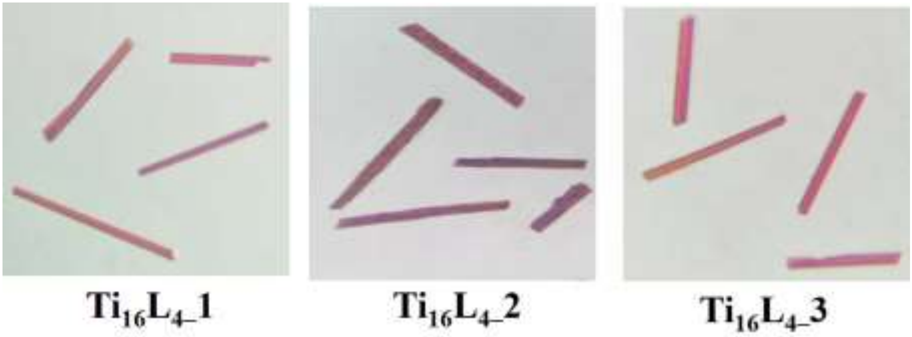

Figure S1. Pictures of fresh crystals of three $Ti_{16}L_4$ clusters separated from the solution.

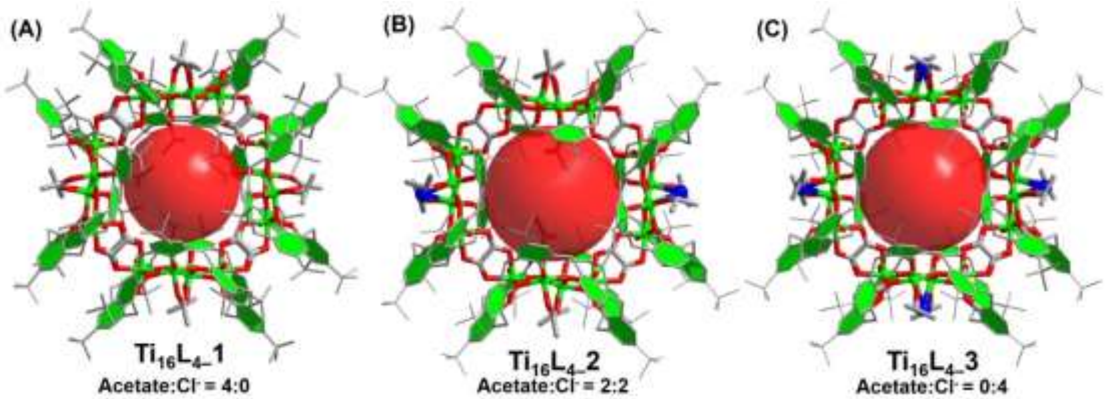

Figure S2. Crystal structures of the three $Ti_{16}L_4$ clusters.

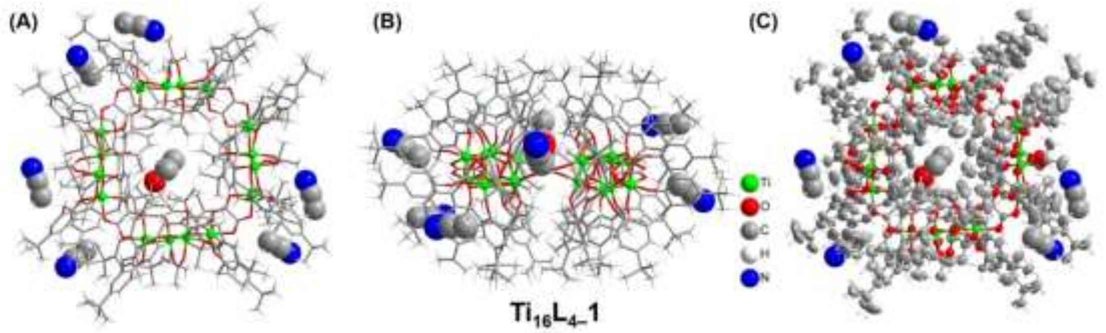

Figure S3. The molecular structure of **$Ti_{16}L_{4}$_1**. (a) and (a) The ball-and-stick view; (b) the ORTEP-style view.

Ti_6L_4_1 is isolated from the acetonitrile. The molecular formula of **$Ti_{16}L_4$_1** was determined as $[H_4Ti_{16}O_8(TBC[8])_4(C_2O_4)_4(Ac)_4(O^iPr)_8]\cdot{}^iPrOH\cdot 13CH_3CN$. Some highly disordered solvent molecules in the crystal structures were removed with the SQUEEZE program in PLATON, and its possible formula was proposed based on the SQUEEZE and TGA results. SCXRD analysis showed the position of some guest molecules. An ^{i}PrOH guest is located inside the macrocycle. Outside the macrocycle, there are $CH_3CN\cdot$ molecules located at the cup mouth of TBC[8]. Interestingly, in $Ti_{16}L_4$, two TBC[8] are adjacent to each other to form a smaller pocket in which a $CH_3CN\cdot$ molecule is anchored through hydrogen bond interactions. The average C-H…N lengths is 2.994 Å.

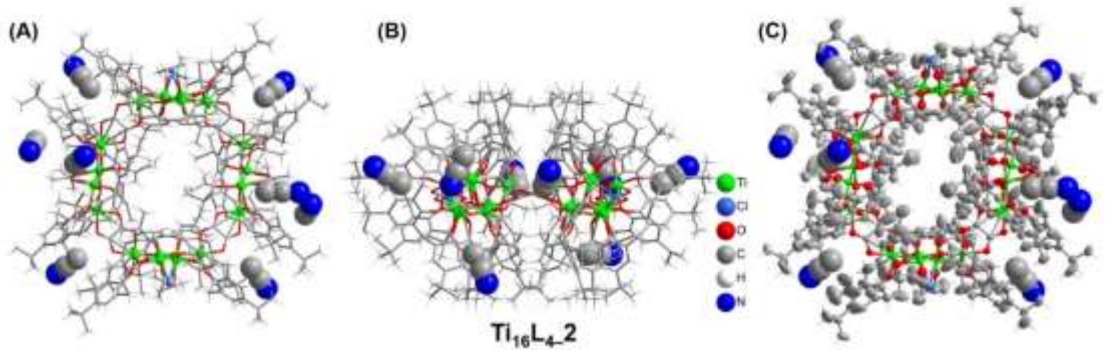

Figure S4. The molecular structure of **$Ti_{16}L_4$_2**. (a) and (a) The ball-and-stick view; (b) the ORTEP-style view.

The molecular formula of **$Ti_{16}L_4$_2** was determined as $[H_4Ti_{16}O_8(TBC[8])_4(C_2O_4)_4(Ac)_2Cl_2(O^iPr)_8]\cdot 56CH_3CN$. Some highly disordered solvent molecules in the crystal structures were removed with the SQUEEZE program in PLATON, and its possible formula was proposed based on the SQUEEZE and TGA results. SCXRD analysis showed the position of some guest molecules for **$Ti_{16}L_4$_2**. Unlike **$Ti_{16}L_4$_1**, no ^{i}PrOH molecules were found inside the ring. Outside the macrocycle, there are acetonitrile molecules located at the cup mouth of the $\{Ti_4O_2(TBC[8])(Ac)(O^iPr)_2\}$ units. No CH_3CN was found at the cup mouth of the $\{Ti_4O_2(TBC[8])(Cl)(O^iPr)_2\}$ units. This may be due to the high electronegativity of Cl^- ions, which

is not conducive to the proximity of acetonitrile molecule. In addition, like **$Ti_{16}L_{4}$_1**, CH_3CN guest is also present in the small pockets formed by the two adjacent TBC[8].

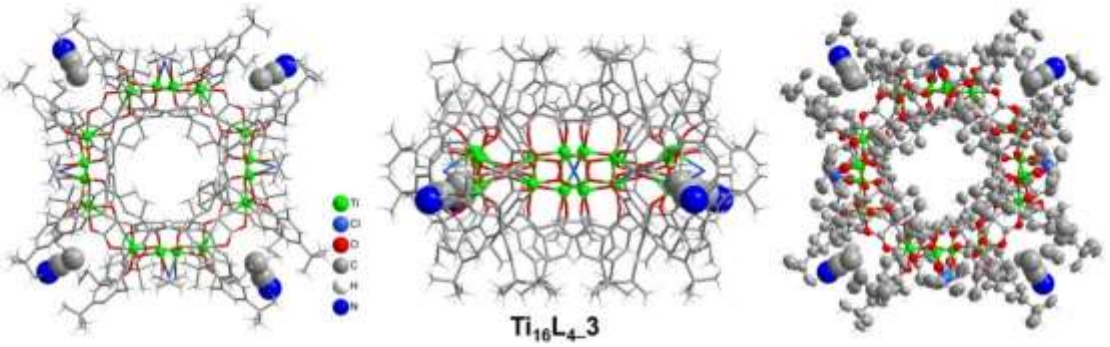

Figure S5. The molecular structure of **$Ti_{16}L_{4}$_3**. (a) and (a) The ball-and-stick view; (b) the ORTEP-style view.

The molecular formula of **$Ti_{16}L_{4}$_3** was determined as $[H_4Ti_{16}O_8(TBC[8])_4(C_2O_4)_4Cl_4(O^iPr)_8]{\cdot}40CH_3CN$. Some highly disordered solvent molecules in the crystal structures were removed with the SQUEEZE program in PLATON, and its possible formula was proposed based on the SQUEEZE and TGA results. SCXRD analysis showed the position of some guest molecules for **$Ti_{16}L_{4}$_3**. Outside the macrocycle, CH_3CN guest is present in the small pockets formed by the two adjacent TBC[8], but no guest molecules were found at the cup mouth of the four $\{Ti_4O_2(TBC[8])(Cl)(O^iPr)_2\}$ units. This is consistent with the situation in **$Ti_{16}L_{4}$_3**.

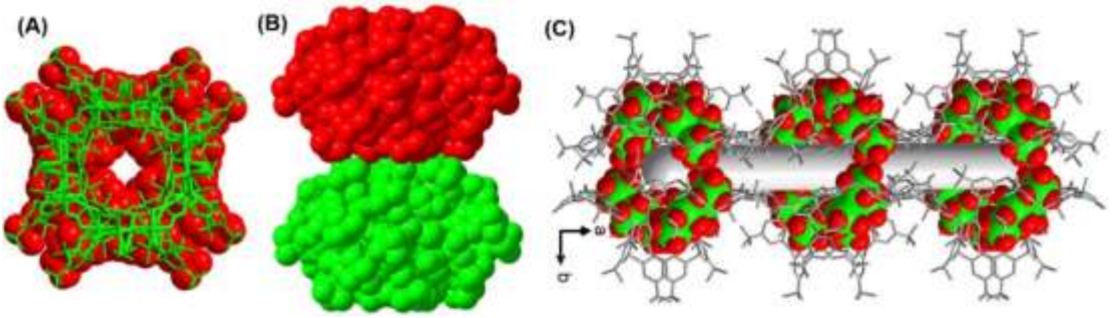

Figure S6. The as-formed nanotube in $Ti_{16}L_4$.

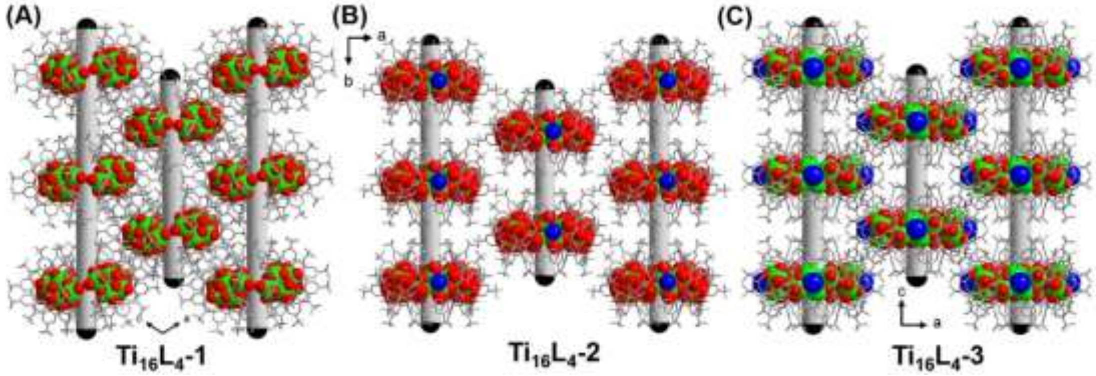

Figure S7. The adjacent nanotubes in $Ti_{16}L_4$ are interlaced with each other to avoid the spatial repulsion between the tert-butyl of the adjacent ring.

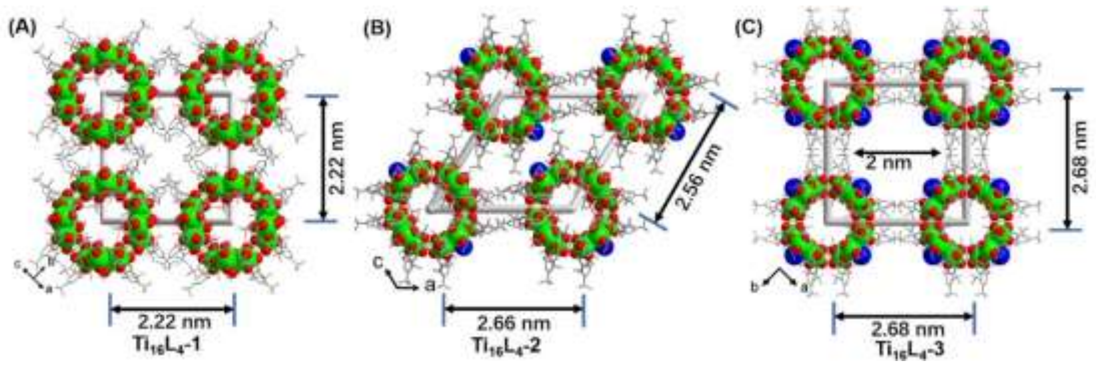

Figure S8. Three patterns of nanotubes arrangement in square array or hexagonal array.

Table S2: Crystallographic data of derived clusters of $Ti_{16}L_4$ obtained through Path I.

Conditions	Soaking the **$Ti_{16}L_4$-1** in 1M formic acid solution for 24h	Soaking the **$Ti_{16}L_4$-1** in 5M formic acid solution for 24 h	Soaking the **$Ti_{16}L_4$-2** in 5M trifluoroacetic acid solution for 24h	Soaking the **$Ti_{16}L_4$-1** in 5M chloroacetic acid solution for 24h	Soaking the **$Ti_{16}L_4$-1** in 5M bromoacetic acid solution for 24h
Compounds	**$Ti_{16}L_4$-1(a)**	**$Ti_{16}L_4$-1**/HFa(a)	**$Ti_{16}L_4$-2**/HTfa	**$Ti_{16}L_4$-1**/HCla	**$Ti_{16}L_4$-1**/HBra
CCDC	2174432	2180762	2287667	2287668	2240657
Cryst. syst.	monoclinic	monoclinic	monoclinic	monoclinic	monoclinic
Space group	C2/c	C2/c	C2/c	C2/c	C2/c
Formula	$C_{380}H_{496}O_{95}Ti_{16}$	$C_{382}H_{479}N_3O_{80}Ti_{16}$	$C_{382}H_{450}F_{12}N_4O_{72}Ti_{16}$	$C_{380}H_{552}Cl_4O_{122}Ti_{16}$	$C_{378}H_{448}Br_4O_{74}Ti_{16}$
F_w	7350.14	7159.05	7243.83	7980.38	7261.38
a[Å]	38.100(2)	37.993(4)	54.372(7)	39.051(3)	39.278(7)
b[Å]	33.157(2)	33.265(4)	20.162(2)	32.810(3)	32.838(6)
c[Å]	37.2491(19)	37.218(4)	50.494(7)	37.614(3)	37.800(6)
β[°]	113.291(2)	113.350(5)	121.984(4)	115.050(3)	115.147(5)
V[Å^3]	43222(4)	43184(9)	46950(10)	43661(6)	44135(13)
Z	4	4	4	4	4
ρ_{calc}g/cm^3	1.130	1.101	1.025	1.214	1.093
μ/mm^{-1}	1.981	1.960	1.823	2.162	2.186
F(000)	15552.0	15136.0	15224.0	16912.0	15200.0
2Θ range for data collection/°	4.394 to 102.69	4.752 to 110	4.886 to 90.208	4.718 to 84.988	3.186 to 96.354
Data/restraints/parameters	35504/446/2338	40988/559/2396	28404/2173/2516	23005/917/2481	42512/1936/2318
GooF	1.061	1.056	1.118	1.118	1.206
R1/wR_2(I> 2σ(I))	0.0750/0.2233	0.0920/0.2646	0.0902/0.2705	0.1329/0.3450	0.1387/0.3608
R1/wR_2(all data)	0.1040/0.2420	0.1283/0.2941	0.1304/0.3036	0.1794/0.3742	0.1732/0.3810

Table S3: Crystallographic data of derived clusters of $Ti_{16}L_4$ obtained through Path II.

Conditions	Soaking the **$Ti_{16}L_4$-1** in 5M NaOH solution for 30 min	Soaking the **$Ti_{16}L_4$-1**/NaOH in formic acid solution for 30 min	Soaking the **$Ti_{16}L_4$-1**/NaOH in 1M acetic acid solution for 30 min	Soaking the **$Ti_{16}L_4$-1**/NaOH in 1M aminoacetic acid	Soaking the **$Ti_{16}L_4$-1**/NaOH in 1M oxalic acid solution for 30 min	Soaking the **$Ti_{16}L_4$-1**/NaOH in 1M glycolic acid for 30 min	Soaking the **$Ti_{16}L_4$-1**/NaOH in 1M phosphoric acid

				solution for 30 min			solution for 30 min
Compounds	**$Ti_{16}L_{4}$-1**/NaOH	**$Ti_{16}L_{4}$-1**/HFa(b)	**$Ti_{16}L_{4}$-1**/HAc	**$Ti_{16}L_{4}$-1**/HAma	**$Ti_{16}L_{4}$-1**/HOa	**$Ti_{16}L_{4}$-1**/HGa	**$Ti_{16}L_{4}$-1**/H_3PO_4
CCDC	2174433	2174435	2174434	2240528	2174438	2174437	2240658
Cryst. syst.	monoclinic	monoclinic	monoclinic	monoclinic	monoclinic	monoclinic	monoclinic
Space group	C2/c	C2/c	C2/c	C2/c	C2/c	C2/c	C2/c
Formula	$C_{366}H_{451}N_3Na_8O_{85}Ti_{16}$	$C_{364}H_{488}Na_4O_{114}Ti_{16}$	$C_{396}H_{568}Na_2O_{141}Ti_{16}$	$C_{384}H_{456}Na_4O_{84}Ti_{16}$	$C_{368}H_{420}O_{76}Ti_{16}$	$C_{372}H_{442}N_2O_{84}Ti_{16}$	$C_{360}H_{424}O_{71}Ti_{16}$
F_w	7202.24	7545.87	8396.85	7273.82	6825.42	7051.65	6873.37
a[Å]	38.447(2)	37.982(7)	39.095(9)	39.187(3)	39.32(4)	38.8238(16)	38.634(3)
b[Å]	32.9077(17)	33.195(6)	33.277(15)	33.088(2)	32.41(4)	32.5568(14)	32.619(3)
c[Å]	37.172(2)	36.904(7)	37.714(16)	37.420(3)	37.78(3)	37.8922(16)	37.291(3)
β[°]	114.933(2)	113.349(6)	114.899(9)	115.189(3)	115.25(3	115.418(2)	114.837(2)
V[Å^3]	42646(4)	42718(14)	44508(30)	43905(6)	43259(3)	43536(74)	42646(6)
Z	4	4	4	4	4	4	Z
ρ_{calc}g/cm^3	1.121	1.173	1.253	1.103	1.228	1.061	1.047
μ/mm^{-1}	1.943	2.056	1.934	1.964	1.954	1.193	1.865
F(000)	15136.0	15944.0	11784.0	15336.0	17056.0	14528.0	14170.0
2Θ range for data collection/°	4.758 to 104.634	4.784 to 96.476	4.694 to 96.42	6.098 to 104.68	4.718 to 84.636	4.702 to 103.078	4.758 to 94.958
Data/restraints/parameters	36171/151/2310	30448/606/2223	31835/407/2397	37451/388/2445	22518/536/2115	35703/375/2225	28360/369/2094
GooF	1.154	1.046	1.064	1.205	1.079	1.096	0.992
R1/wR$_2$(I>2σ(I))	0.1112/0.3308	0.0929/0.2702	0.0924/0.2705	0.1148/0.3256	0.1595/0.3789	0.0810/0.2623	0.1242/0.3362
R1/wR$_2$(all data)	0.1265/0.3440	0.1313/0.3040	0.1306/0.3031	0.1470/0.3510	0.2193/0.4372	0.0929/0.2729	0.1727/0.3708

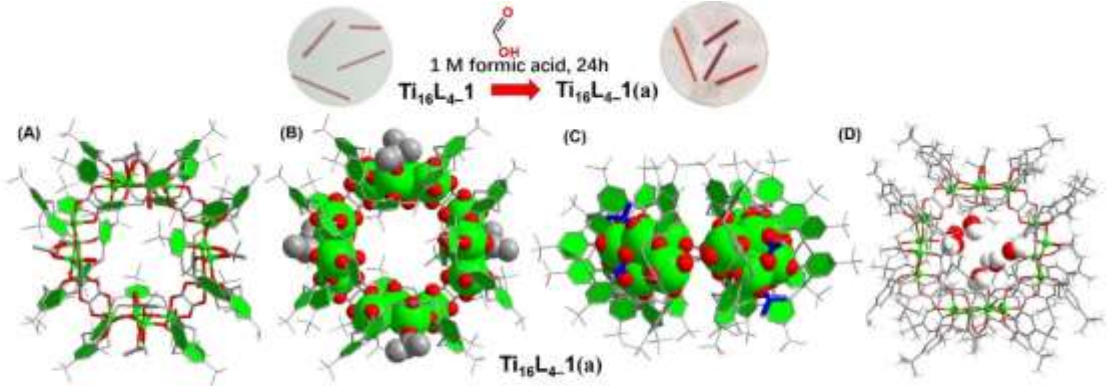

Figure S9. (A-C) Crystal structures of the **$Ti_{16}L_{4}$-1**(a), emphasizing its nano-sized dimensions (top and side views). SCXRD analysis showed that the four internal ^{i}PrO^{-} sites were replaced by H_2O molecules, but the four acetic acid sites and four internal ^{i}PrO^{-} sites remained unchanged. (D) SCXRD analysis showed the position of some guest molecules for **$Ti_{16}L_{4}$-1**(a). Because the crystal is immersed in water solution for a long time, the H_2O molecular occupies the inner space of the macrocycle.

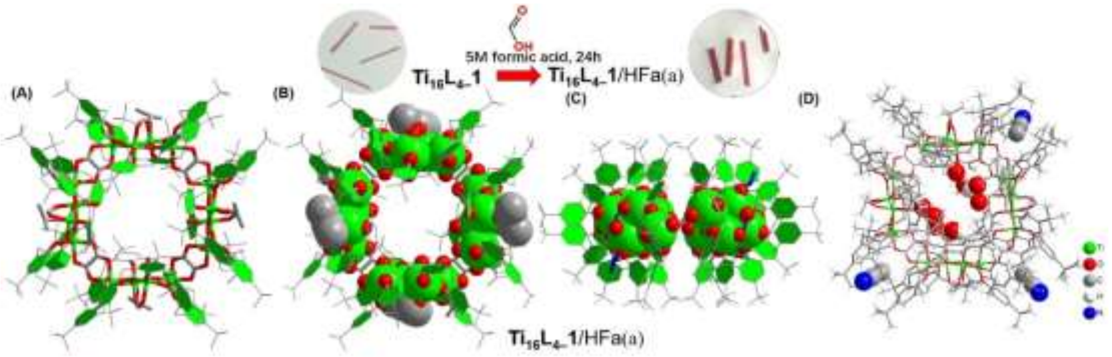

Figure S10. (A-C) Crystal structures of the **$Ti_{16}L_{4}$-1**/HFa(a), emphasizing its nano-sized dimensions (top and side views). SCXRD analysis showed that four acetate sites were replaced by formate ligands, and four internal $^{i}PrO^{-}$ sites were replaced by H_2O molecules, but the four external $^{i}PrO^{-}$ sites remained unchanged. (D) SCXRD analysis showed the position of some guest molecules for **$Ti_{16}L_{4}$-1**/HFa(a). The inner space of the macrocycle is occupied by H_2O molecules. No CH_3CN guest molecules were found at the cup mouth of the four {Ti_4O_2(TBC[8])} units. However, in the small pockets formed by the two adjacent {Ti_4O_2(TBC[8])} units, the CH_3CN guest is still existed.

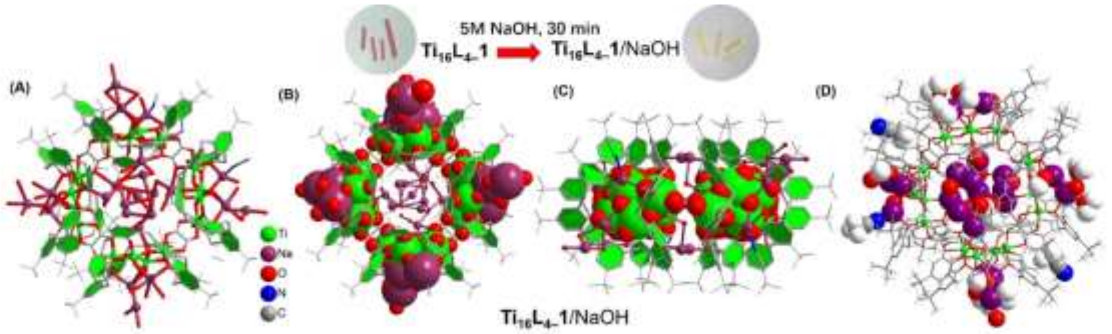

Figure S11. (A-C) Crystal structures of the **$Ti_{16}L_{4}$-1**/NaOH, emphasizing its nano-sized dimensions (top and side views). The surface acetate and $^{i}PrO^{-}$ ligands are fully exchanged by the OH^{-} ion; (D) SCXRD analysis showed the position of some guest molecules for **$Ti_{16}L_{4}$-1**/NaOH. Because the crystal is immersed in NaOH solution, and a large amount of Na^{+} ions are embedded in the ring via OH^{-} bridging. The Ti:Na ration in **$Ti_{16}L_{4}$-1**/NaOH was confirmed to be about 2.07:1 by ICP-AES analysis, which is almost consistent with EDS results. Interestingly, in the small pockets formed by the two adjacent {Ti_4O_2(TBC[8])} units, the CH_3CN guest is still existed. This position can be thought of as a molecular cage, firmly trapping CH_3CN molecules inside.

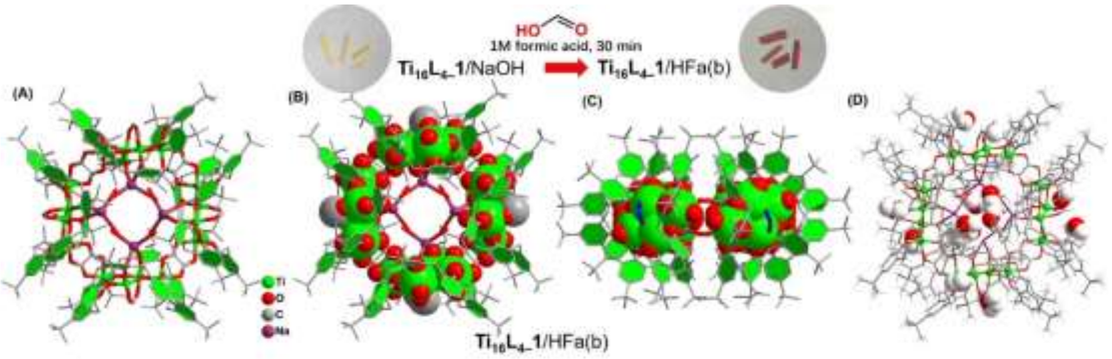

Figure S12. (A-C) Crystal structures of the **$Ti_{16}L_{4}$-1**/HFa(b), emphasizing its nano-sized dimensions (top and side views). The four μ_2-OH^{-} sites were replaced by the formate ligands in the μ_2-(O,O') form again. The remaining eight OH^{-} sites are still occupied by water molecules. (D) SCXRD analysis showed the position of some guest molecules for **$Ti_{16}L_{4}$-1**/HFa(a). Four Na^{+} ions are embedded in the cluster through Fa^{-} bridging. The remaining space inside the macrocycle is occupied by H_2O molecules.

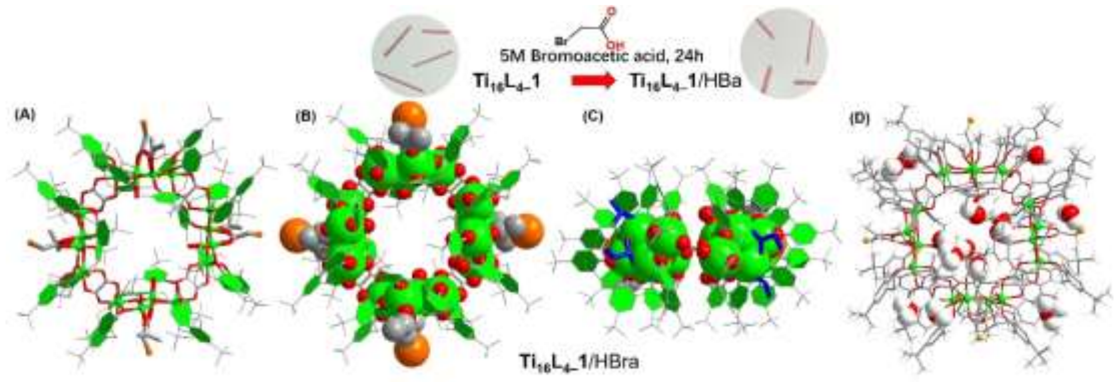

Figure S13. (A-C) Crystal structures of the **$Ti_{16}L_{4}$_1**/HBra, emphasizing its nano-sized dimensions (top and side views). SCXRD analysis showed that four acetic acid sites were replaced by bromoacetate ligands, and four internal $^{i}PrO^{-}$ sites were replaced by H_2O molecules, but the four external $^{i}PrO^{-}$ sites remained unchanged; (D) Because the crystal is immersed in water solution for a long time, the H_2O molecular occupies the inner space of the macrocycle.

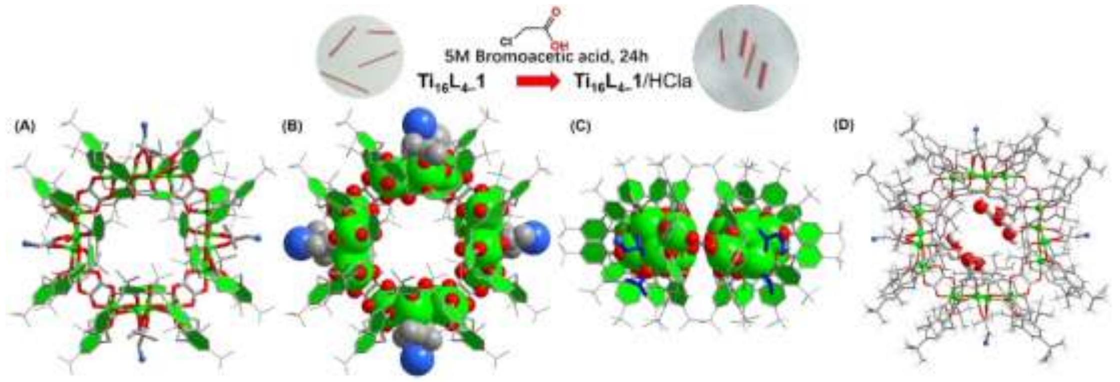

Figure S14. (A-C) Crystal structures of the **$Ti_{16}L_{4}$_1**/HCla, emphasizing its nano-sized dimensions (top and side views). SCXRD analysis showed that four acetic acid sites were replaced by chloroacetate ligands, and four internal $^{i}PrO^{-}$ sites were replaced by H_2O, but the four external $^{i}PrO^{-}$ sites remained unchanged; (D) Because the crystal is immersed in water solution for a long time, the H_2O molecular occupies the inner space of the macrocycle.

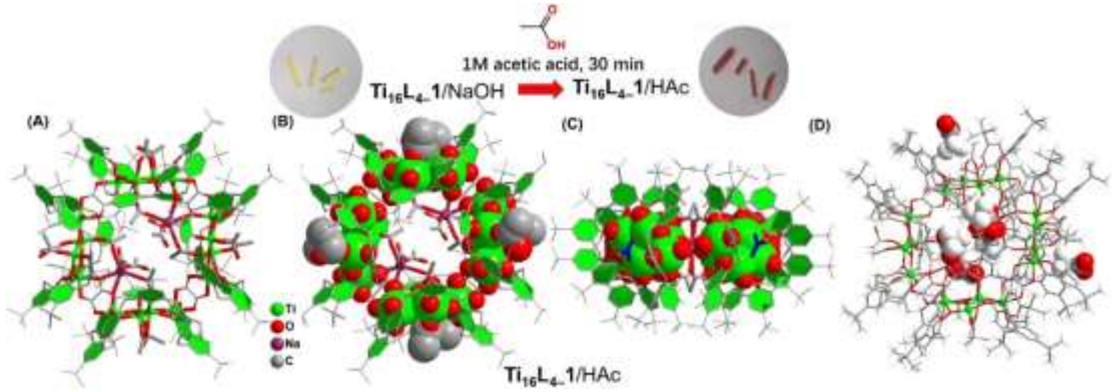

Figure S15. (A-C) Crystal structures of the **$Ti_{16}L_{4}$_1**/HAc, emphasizing its nano-sized dimensions (top and side views). All twelve OH^{-} sites were replaced by acetate ligands again. (D) SCXRD analysis showed the position of some guest molecules for **$Ti_{16}L_{4}$_1**/HAc. Two Na^{+} ions are embedded in the cluster through acetate bridging, and four additional acetic acid molecules are embedded into the ring through coordination with Na^{+} ions. No CH_3CN guest molecules are found outside the ring.

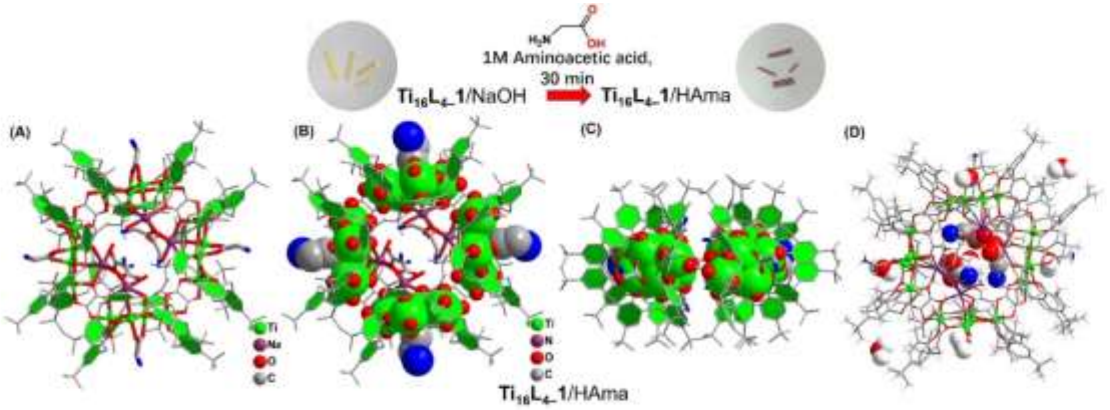

Figure S16. (A-C) Crystal structures of the **$Ti_{16}L_{4}$_1**/HAma, emphasizing its nano-sized dimensions (top and side views). The four μ_2-OH^- sites were replaced by the aminoacetate ligands in the μ_2-(O,O') form. The remaining eight OH^- sites are still occupied by water molecules; (D) SCXRD analysis showed the position of some guest molecules for **$Ti_{16}L_{4}$_1**/HAma. Four Na^+ ions are embedded in the cluster through acetate bridging, and four additional aminoacetic acid molecules are embedded into the ring through coordination with Na^+ ions. No CH_3CN guest molecules are found outside the ring.

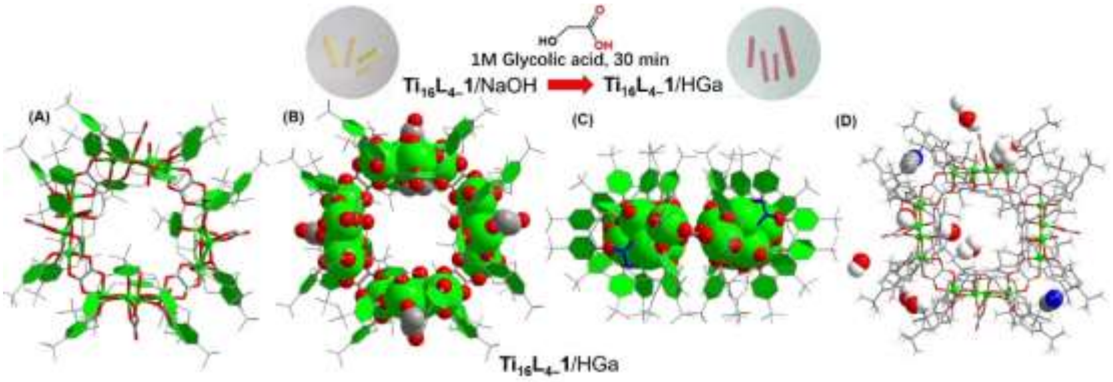

Figure S17. (A-C) Crystal structures of the **$Ti_{16}L_{4}$_1**/HGa, emphasizing its nano-sized dimensions (top and side views). Four oxalate ligands coordinate with the Ti3 site in chelate-bidentate μ_1-(O,O) mode, while eight OH^- ions are coordinated with Ti2 sites. (D) SCXRD analysis showed the position of some guest molecules for **$Ti_{16}L_{4}$_1**/HGa. No residual Na^+ ions were found in the structure. The H_2O molecular occupies the inner space of the macrocycle. In the small pockets formed by the two adjacent {Ti_4O_2(TBC[8])} units, the CH_3CN guest is still existed.

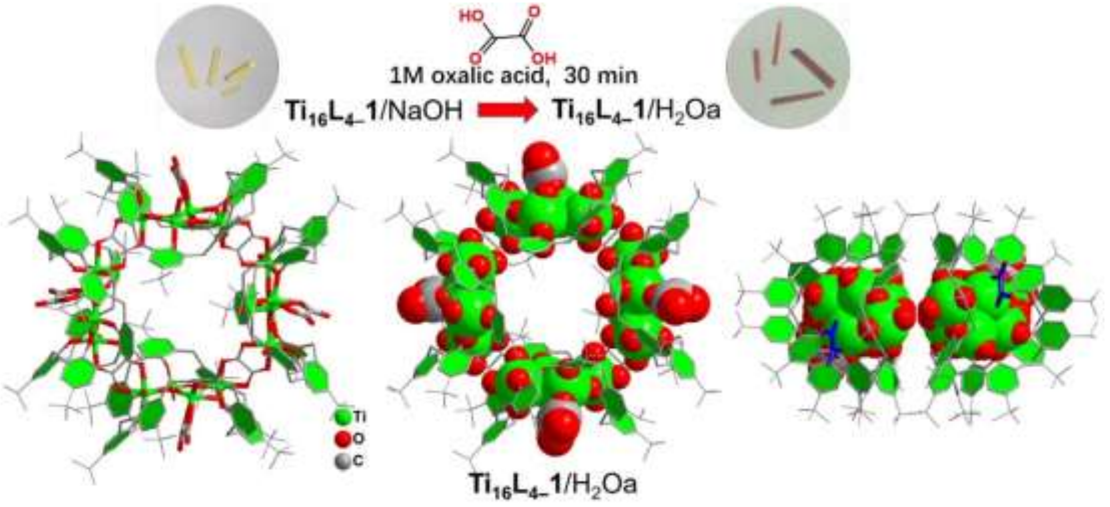

Figure S18. Crystal structures of the **$Ti_{16}L_{4}$_1**/H_2Oa, emphasizing its nano-sized dimensions (top and side views). Four glycolate ligands coordinate with the Ti3 site in chelate-bidentate μ_1-(O,O) mode, while eight OH^- ions are coordinated with Ti2 sites. Due to the weak diffraction data of the crystal, we have not been able to determine the position of the guest molecules.

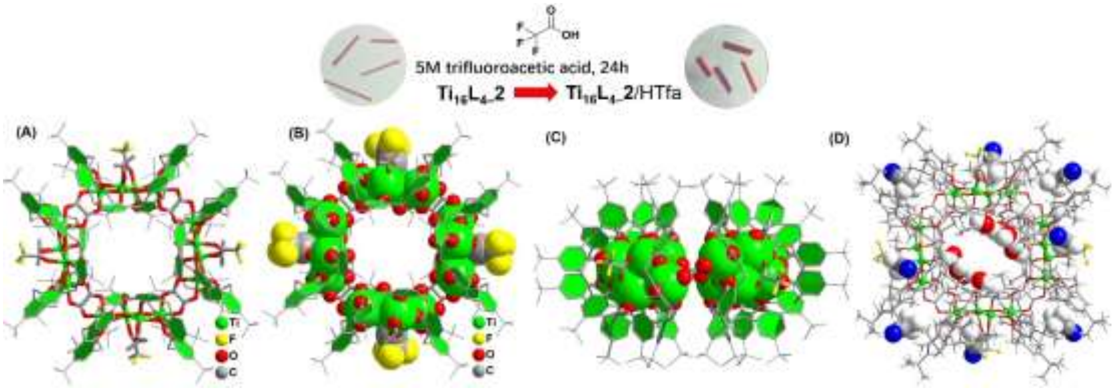

Figure S19. (A-C) Crystal structures of the **$Ti_{16}L_{4}$_2**/HTfa, emphasizing its nano-sized dimensions (top and side views). SCXRD analysis showed that four acetic acid sites were replaced by trifluoroacetate, and four internal $^{i}PrO^-$ sites were replaced by H_2O, but the four external $^{i}PrO^-$ sites remained unchanged; (D) Because the crystal is immersed in water solution for a long time, the H_2O molecular occupies the inner space of the macrocycle.

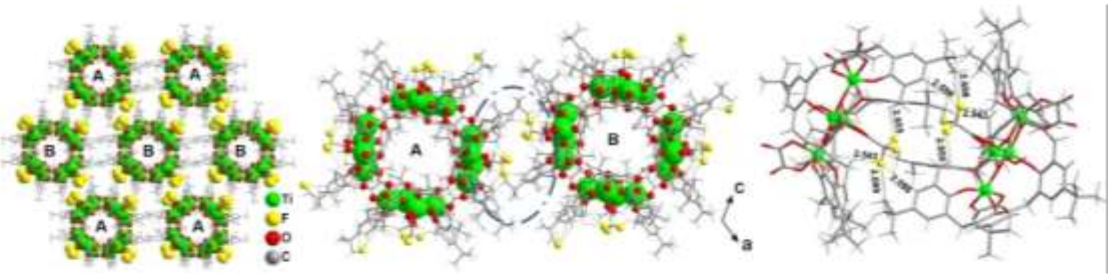

Figure S20. The hexagonal array of the nanotube in **$Ti_{16}L_{4}$_2**/HTfa. Illustration of the H bonds between two interlocked clusters of **$Ti_{16}L_{4}$_2**/HTfa.

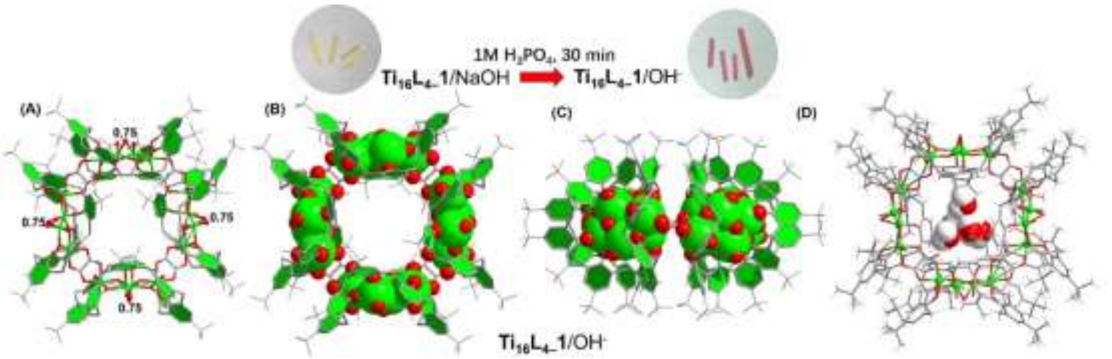

Figure S21. (A-C) Crystal structures of the **$Ti_{16}L_{4}$_1**/OH^-, emphasizing its nano-sized dimensions (top and side views). SXRD showed that 12 unstable sites were still occupied by OH^- ions and H_2O molecules, and all Na^+ ions were detached from the structure; (D) SCXRD analysis showed the position of some guest molecules for **$Ti_{16}L_{4}$_1**/OH^-. The H_2O molecular occupies the inner space of the macrocycle. No CH_3CN guest molecule was found after two exchanges in the acid-base.

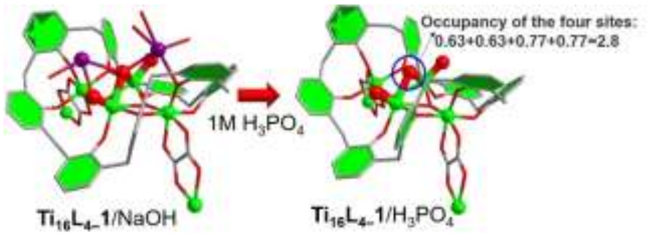

Figure 22. The crystals obtained by soaking **$Ti_{16}L_{4}$-1**/NaOH in H_3PO_4. Only the {Ti_4-TBC[8]} subunit is given here. Notably, structural refinements indicate that the proportion of each μ_2-O site is only 0.75, and these four μ_2-bridging fashion sites have a total of three OH ions. This means that after two ligand exchanges, one of the four μ_2-bridging sites on the cluster is eventually deleted.

3. Powder X-ray diffraction

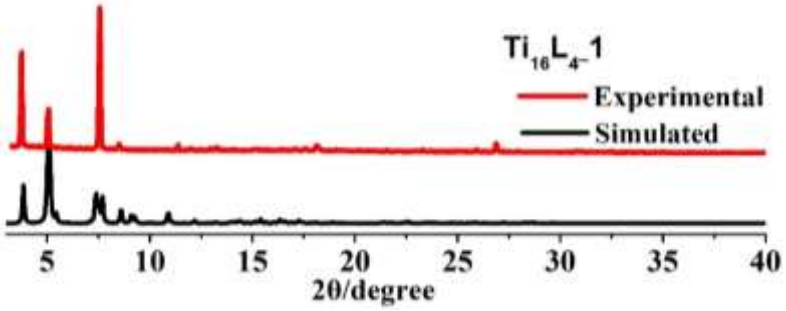

Figure S23. The XRD pattern of **$Ti_{16}L_{4}$-1**.

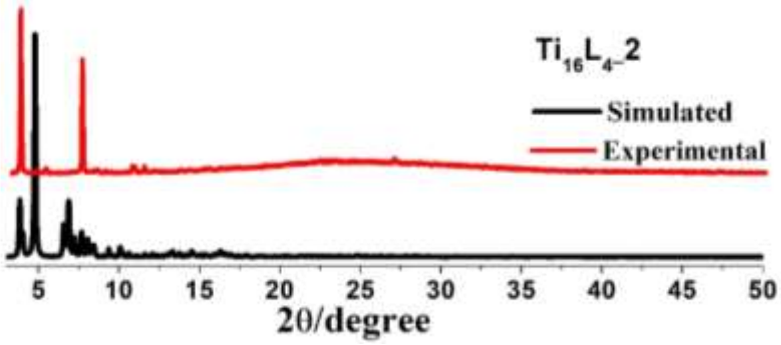

Figure S24. The XRD pattern of **$Ti_{16}L_{4}$-2**.

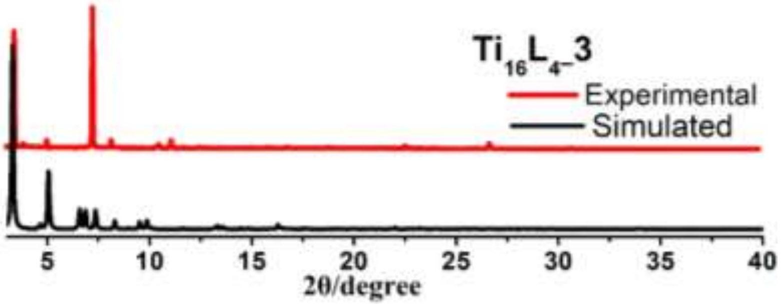

Figure S25. The XRD pattern of **$Ti_{16}L_{4}$-3**.

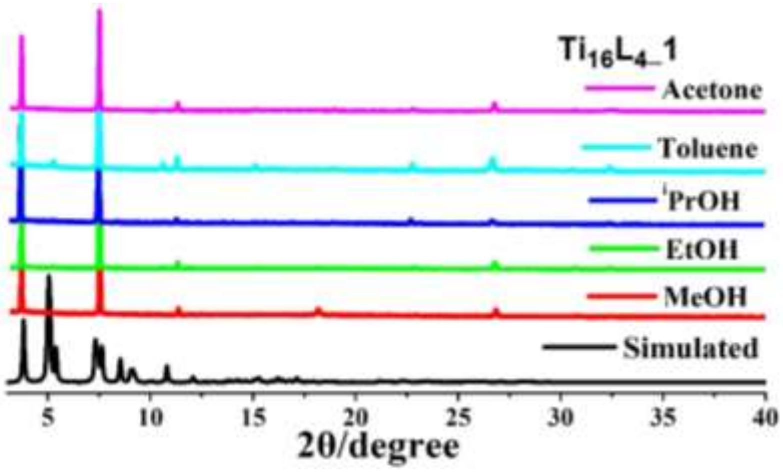

Figure S26. XRD patterns of **$Ti_{16}L_{4}$_1** treated in different organic solvents at room temperature for one week.

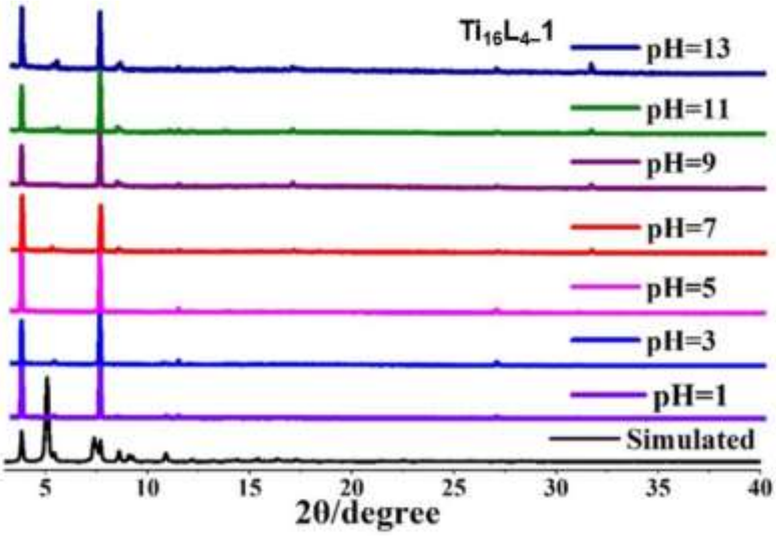

Figure S27. XRD patterns of **$Ti_{16}L_{4}$_1** treated in different pH solutions at room temperature for 24 hours.

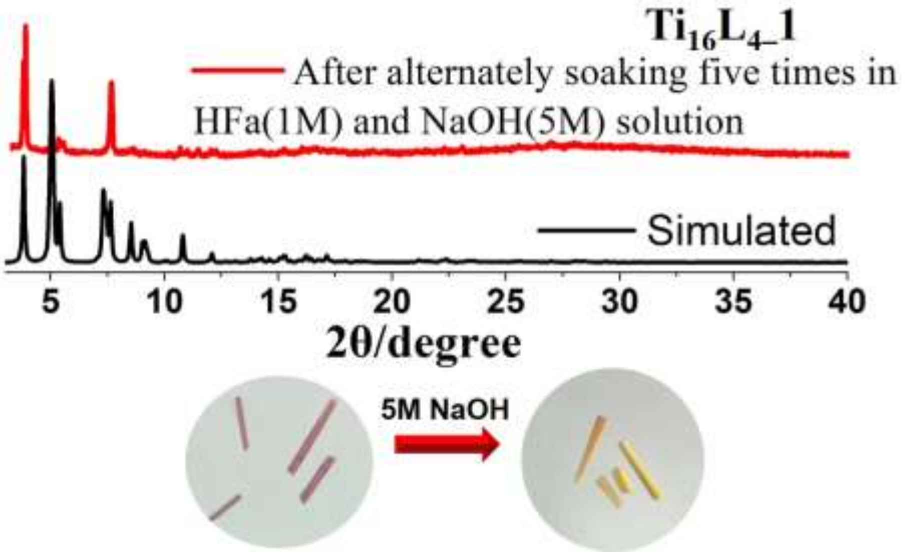

Figure S28. XRD patterns of **$Ti_{16}L_{4}$_1** after alternating soaking in 5M NaOH and 1M HFa solution for five times. After five times of alternating soaking, the crystal is still firm and the color can obviously change between yellow and red.

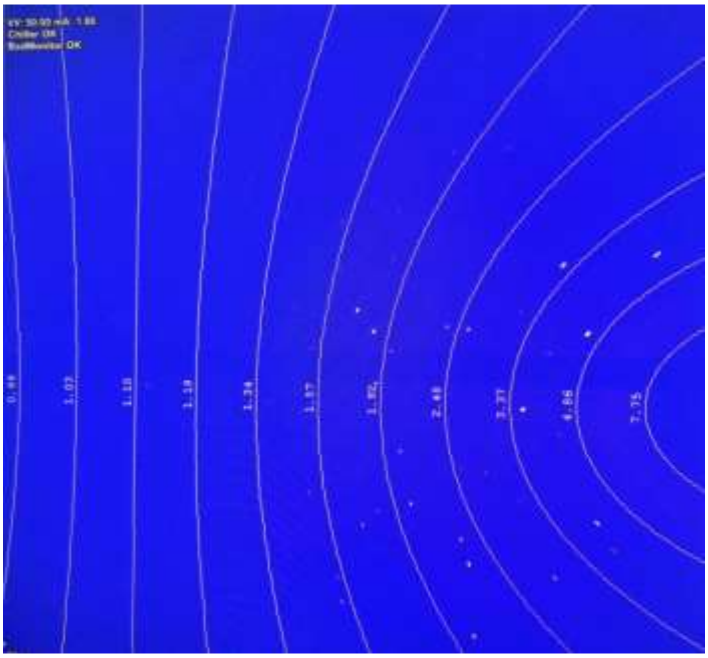

Figure S29. The diffraction images of the crystal of **$Ti_{16}L_{4}$-1** after alternating soaking in 5M NaOH and 1M HFa solution for five times.

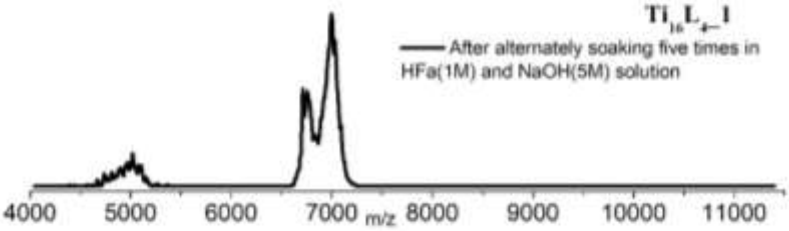

Figure S30. The diffraction images of the crystal of **$Ti_{16}L_{4}$-1** after alternating soaking in 5M NaOH and 1M HFa solution for five times.

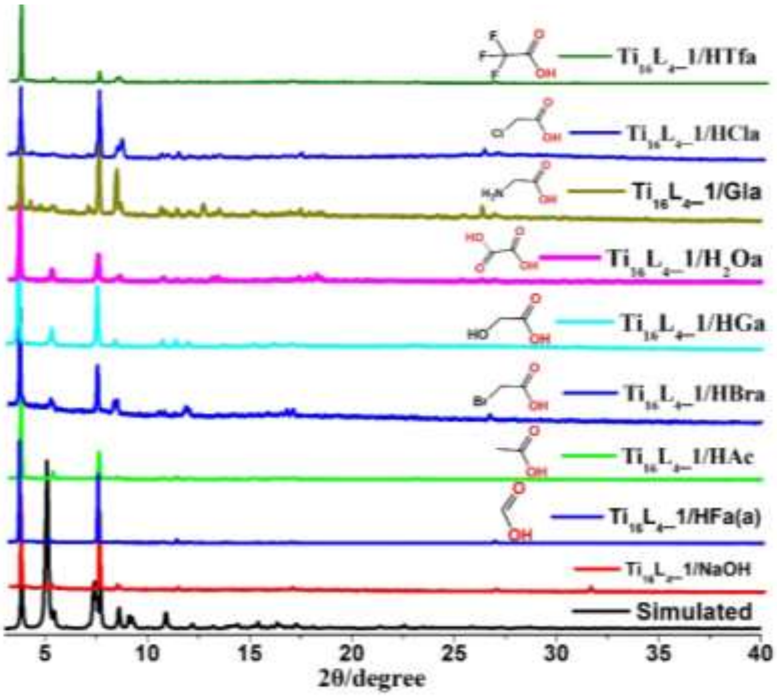

Figure S31. XRD patterns of the derived clusters of **$Ti_{16}L_{4}$-1** through post substitution methods.

4. XPS Measurement

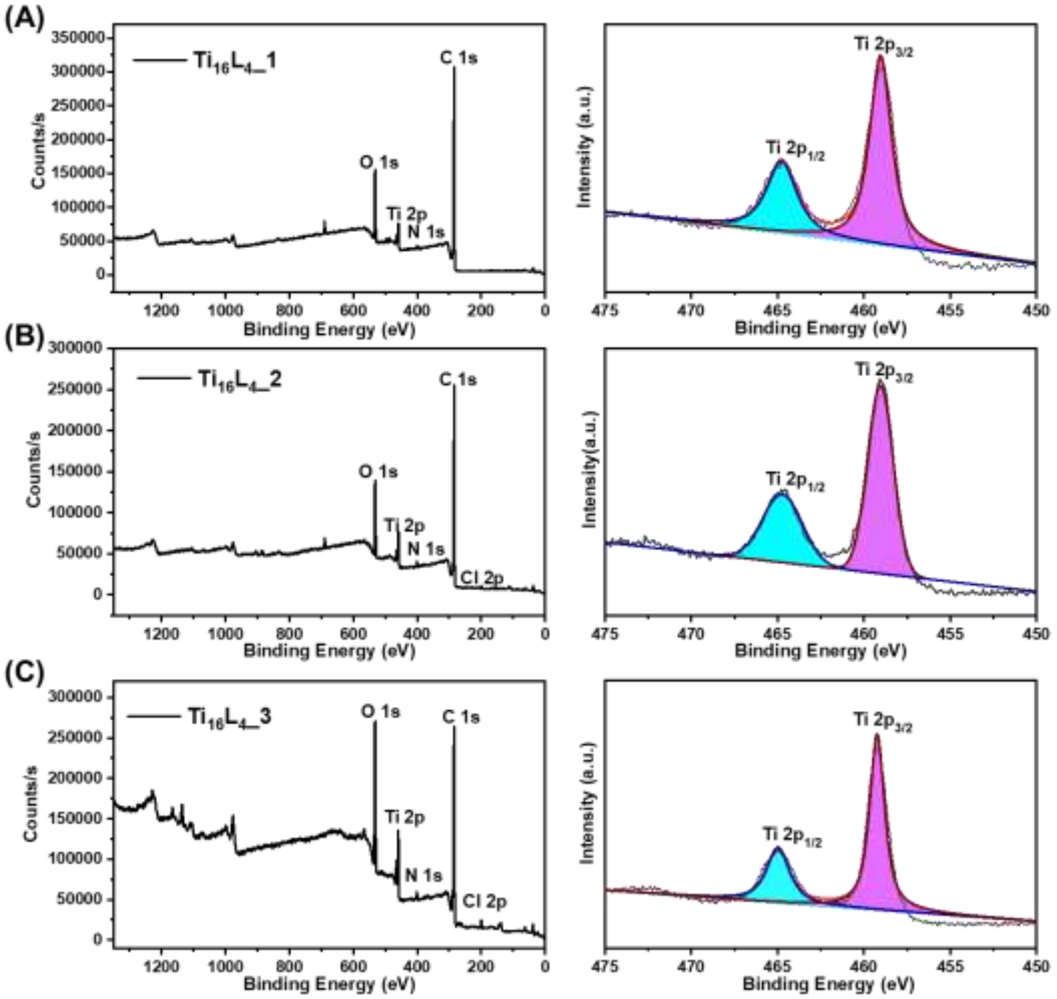

Figure S32. XPS Measurements for $Ti_{16}L_4$. The presence of Ti^{4+} has been verified by the XPS analysis. In the case of **$Ti_{16}L_4$_1**, the Ti 2p spectrum exhibits distinct Ti $2p^{1/2}$ (ca. 464.9 eV) and Ti $2p^{3/2}$ (ca. 459.0 eV) regions, showing a binding energy difference of 5.9 eV. This discrepancy indicates the presence of Ti^{4+}. In the XPS spectra of **$Ti_{16}L_4$_2** and **$Ti_{16}L_4$_3**, shifts in the binding energy peak of Ti are observed, attributed to ligand modifications.

5. TG Measurement

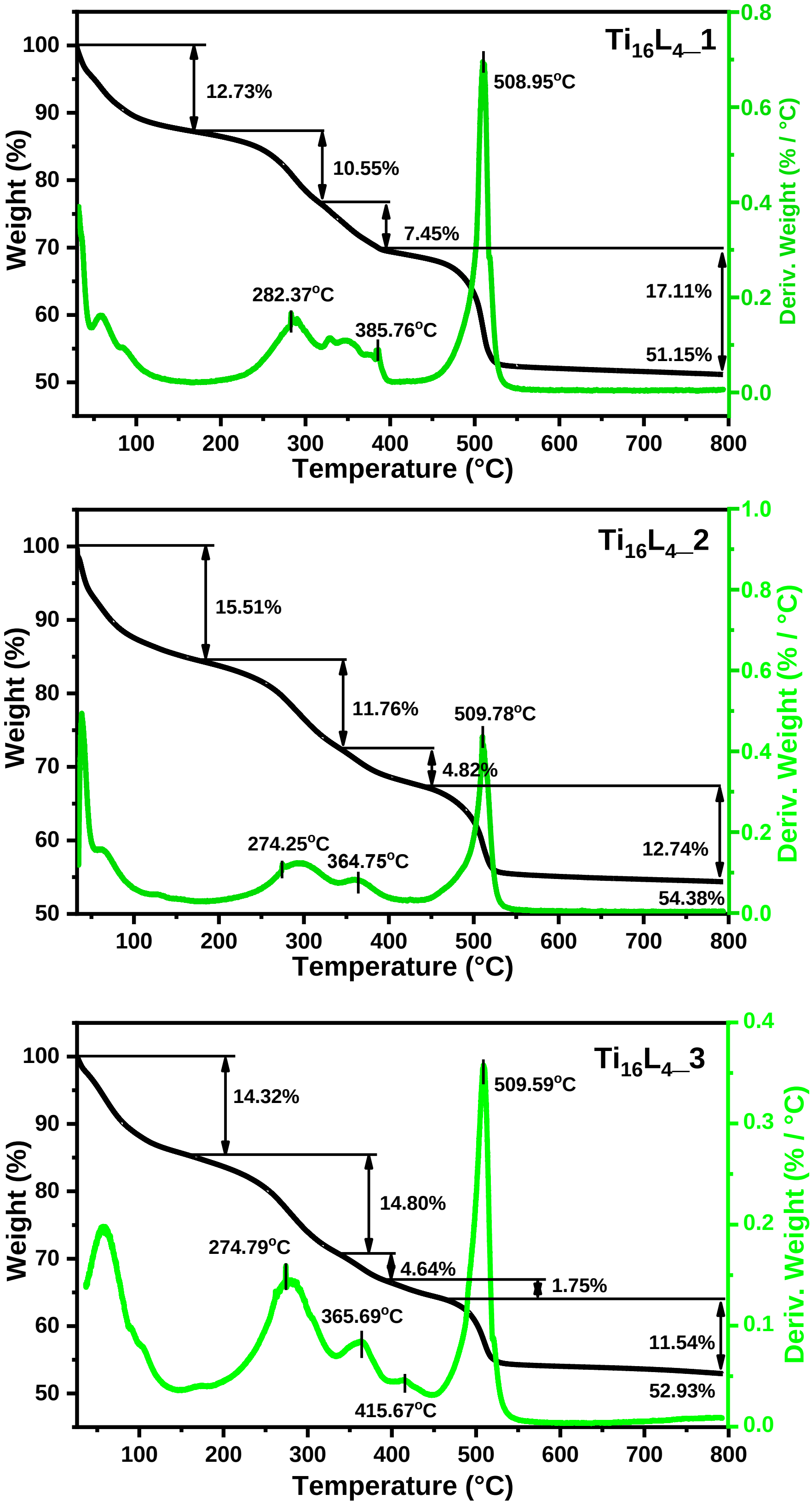

Figure S33. TGA curve of **$Ti_{16}L_{4}$_1**, **$Ti_{16}L_{4}$_2** and **$Ti_{16}L_{4}$_3**.

6. IR Spectra

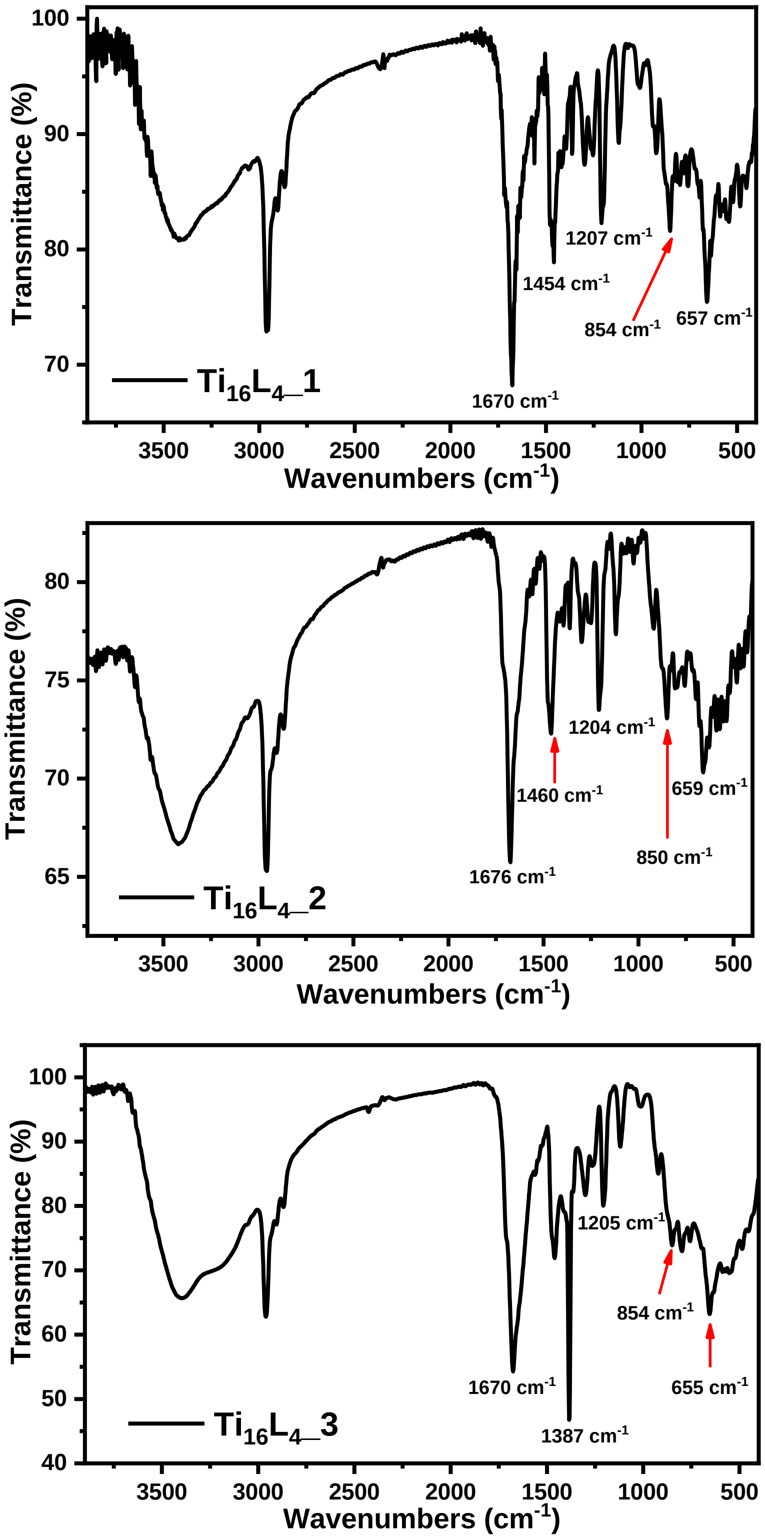

Figure S34. IR spectra of crystal samples of **$Ti_{16}L_4$_1**, **$Ti_{16}L_4$_2** and **$Ti_{16}L_4$_3**. In the high wavenumber region, the aboard absorption bands from 3700 to 3200 cm^{-1} stem from the ν(O-H) stretching mode of -OH groups or water molecules. The weak absorption bands at 3000−2800 cm^{-1} can be ascribed to the stretching vibration modes of C–H bonds in aromatic rings and tert butyl group. The characteristic stretching vibrations ν(CO_2^-) of in carboxylic groups and ν(C=C) in benzene rings are overlapped from 1600 cm^{-1} to 1400 cm^{-1}. The characteristic bands of Ti-O-C and Ti-O-Ti appears in the ranges of 1000–1200 and 700–800 cm^{-1}, respectively.[1-2]

Reference

1. Zhang, G., Hou, J., Li, M., Tung, C., Wang, Y., Counteranion-Stabilized Titanium(IV) Isopolyoxocationic Clusters Isolated from Water. *Inorg. Chem.* 2016, **55**, 4704-4709.

2. Zhang, G., Liu, C., Long, D., Cronin, L., Tung, C., Wang, Y., Water-Soluble Pentagonal-Prismatic Titanium-Oxo Clusters. *J. Am. Chem. Soc.* 2016, **138**, 11097–11100.

7. Energy Dispersive X-ray (EDX) Spectroscopic Analysis

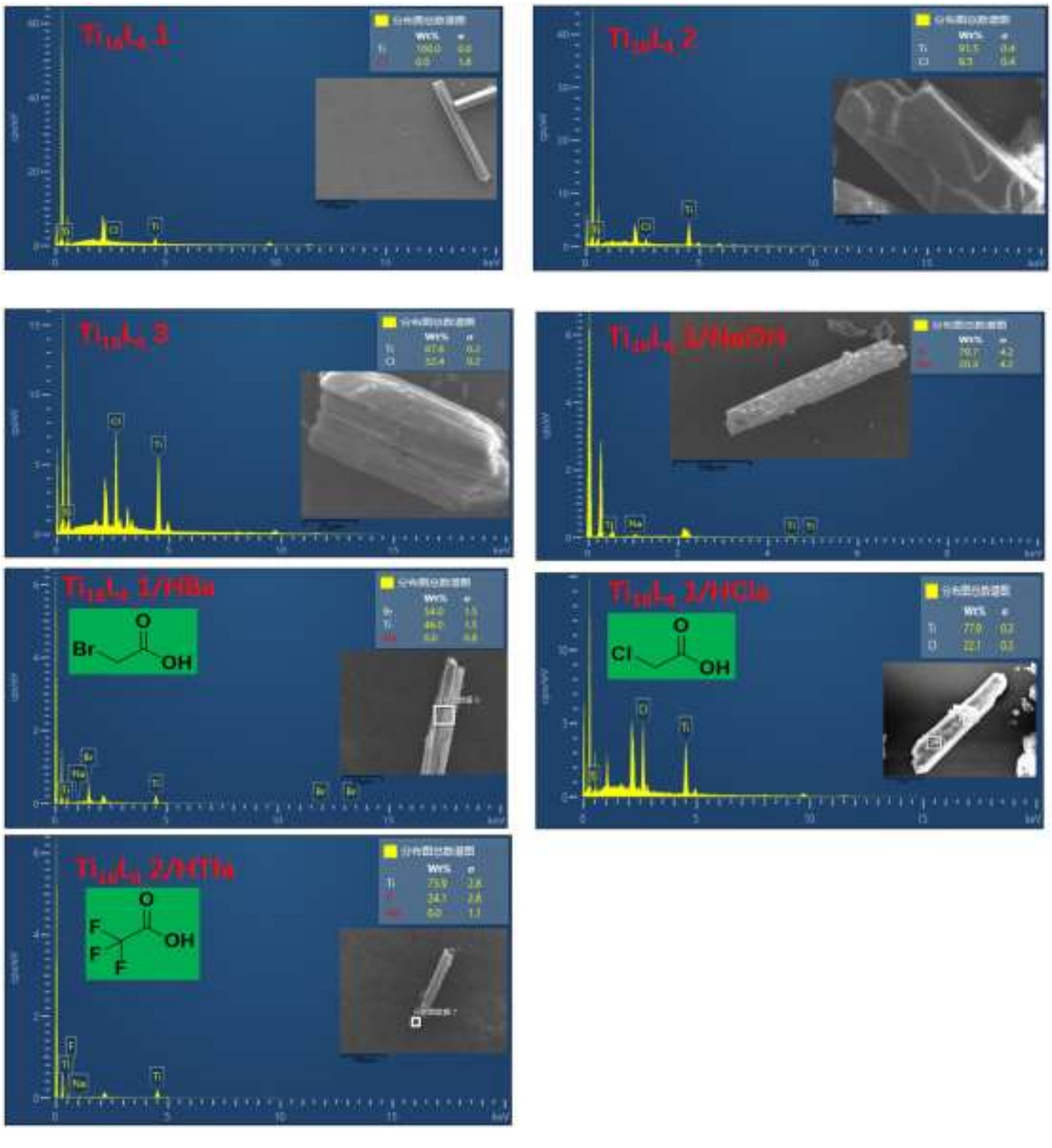

Figure S35. The EDS patterns of **$Ti_{16}L_4$-1**, **$Ti_{16}L_4$-2**, **$Ti_{16}L_4$-3**, **$Ti_{16}L_4$-1**/NaOH, **$Ti_{16}L_4$-1**/HBa, **$Ti_{16}L_4$-1**/HCla and **$Ti_{16}L_4$-2**/HTfa. The presence of Cl^- ions in **$Ti_{16}L_4$-2** and **$Ti_{16}L_4$-3**, Na ions in **$Ti_{16}L_4$-1**/NaOH, Br in **$Ti_{16}L_4$-1**/HBa, Cl in **$Ti_{16}L_4$-1**/HCla, and F in **$Ti_{16}L_4$-2**/HTfa can be demonstrated by EDS.

8. CO_2 Adsorption

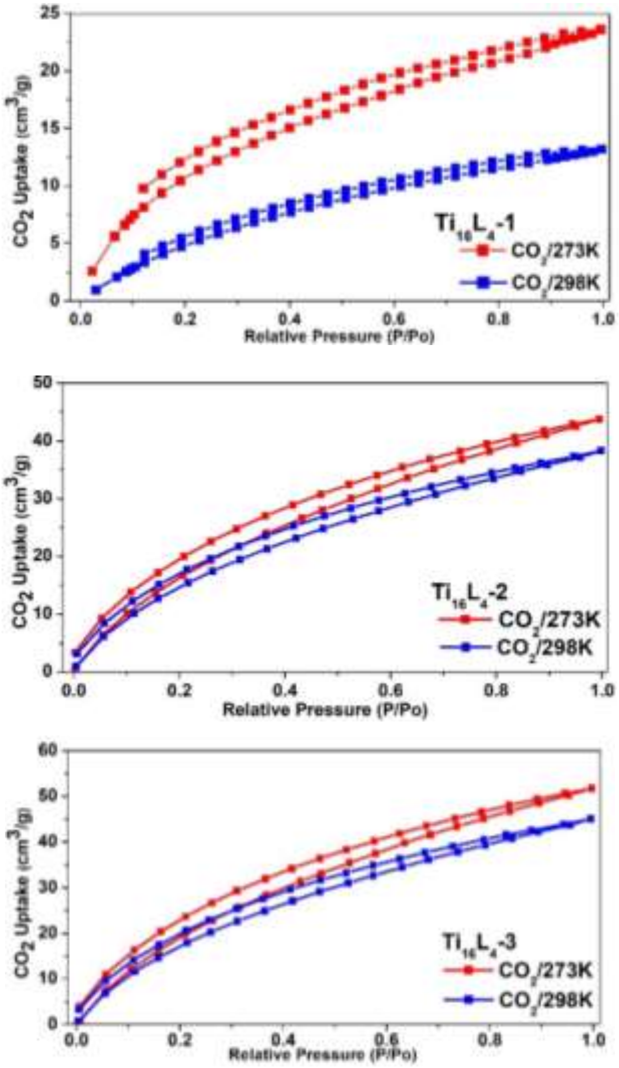

Figure S36. CO_2 adsorption and desorption isotherms of **$Ti_{16}L_{4}$-1, $Ti_{16}L_{4}$-2** and **$Ti_{16}L_{4}$-3** at 273 K (red) and 298 K (blue).

9. Dynamic Light Scattering Measurement

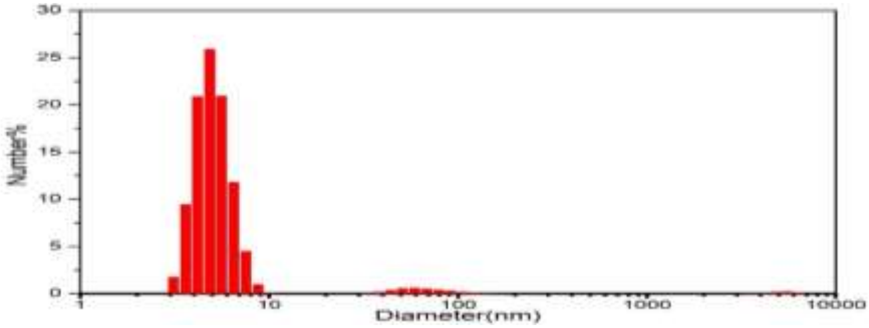

Dynamic light scattering (DLS) measurement is performed on a Malvern Zetasizer 1000HSA. The sample of **$Ti_{16}L_{4}$-1** was dissolved in chloroform solution by ultrasonic dispersion. The solution was centrifuged. The clear solution was sucked out and placed in a quartz cuvette for measurement. In the range of 1-1000 nm, only one size distribution is observed with an average diameter of 4.2 nm, which matches the size of the cage (~ 3.0 nm) and verifies the existence of the cluster molecules in solution.

Figure S37. DLS of **$Ti_{16}L_{4}$-1** in chloroform solution.

10. Wettability of $Ti_{16}L_4$ Rings

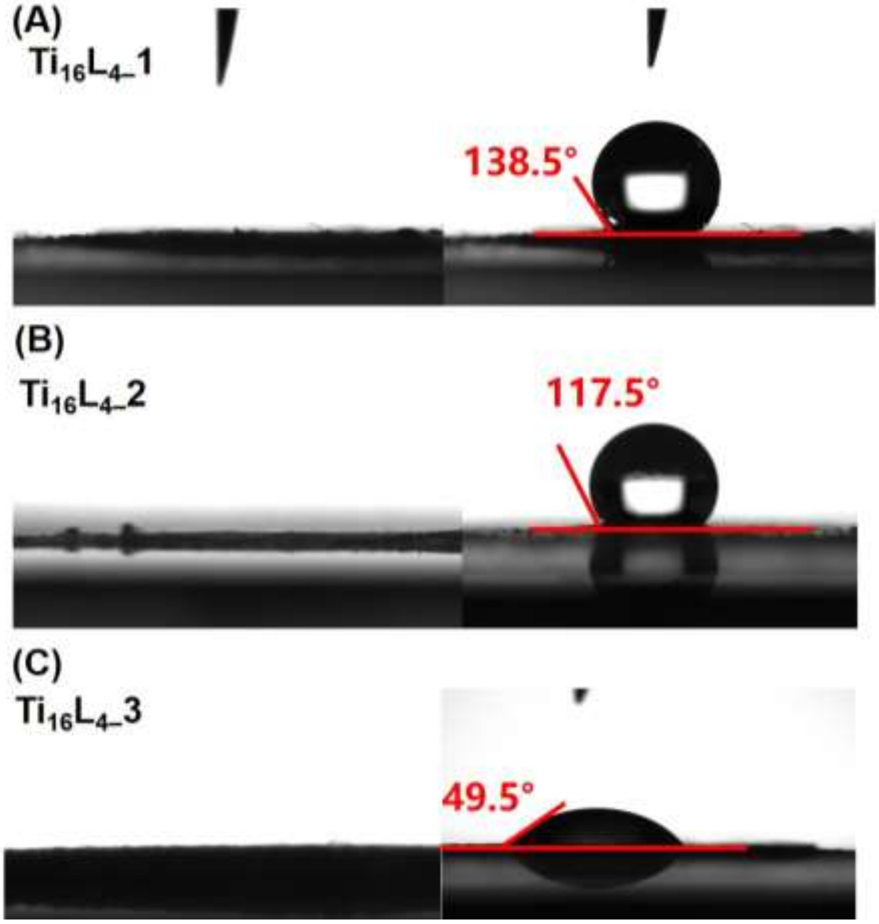

Figure S38. Contact angle patterns for **$Ti_{16}L_4$_1/2/3**. The contact angles measured on as-synthesized powder samples of **$Ti_{16}L_4$_1/2/3** were 138.5°, 117.5° and 49.5°, respectively.

11. Photocatalysis

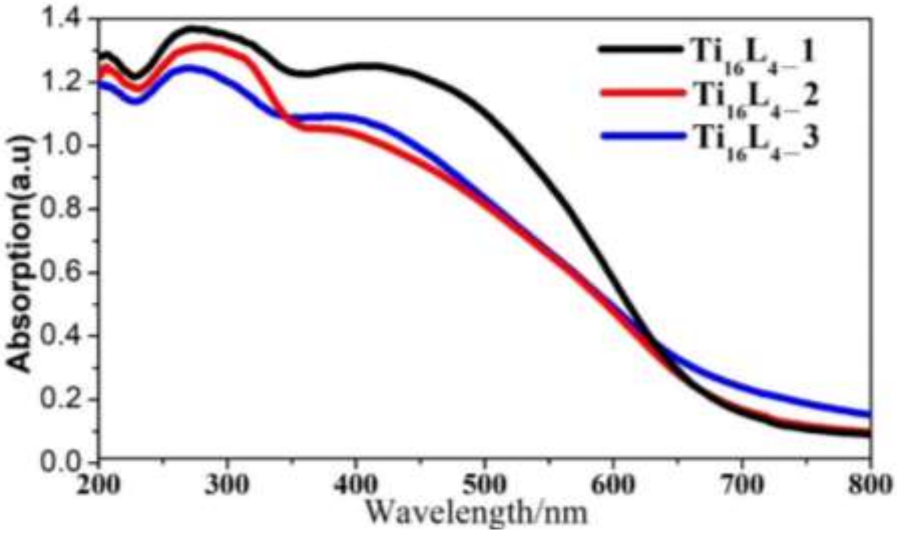

Figure S39. Solid state UV-Vis spectra of **$Ti_{16}L_4$_1/2/3.**

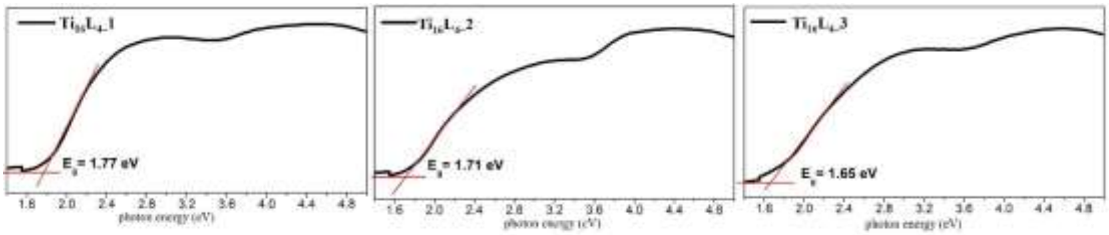

Figure S40. Tauc plot of **$Ti_{16}L_4$_1/2/3** derived from UV-vis spectra.

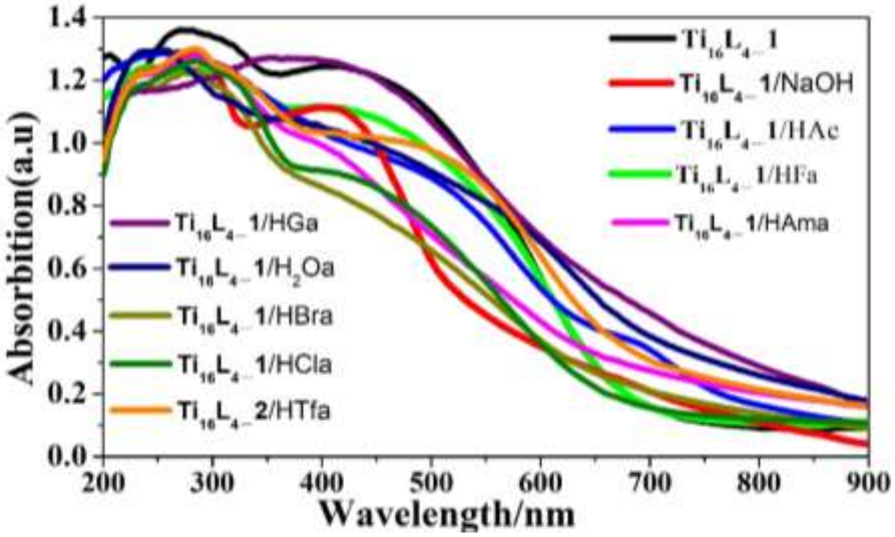

Figure S41. Solid state UV-Vis spectra of the derived $Ti_{16}L_4$.

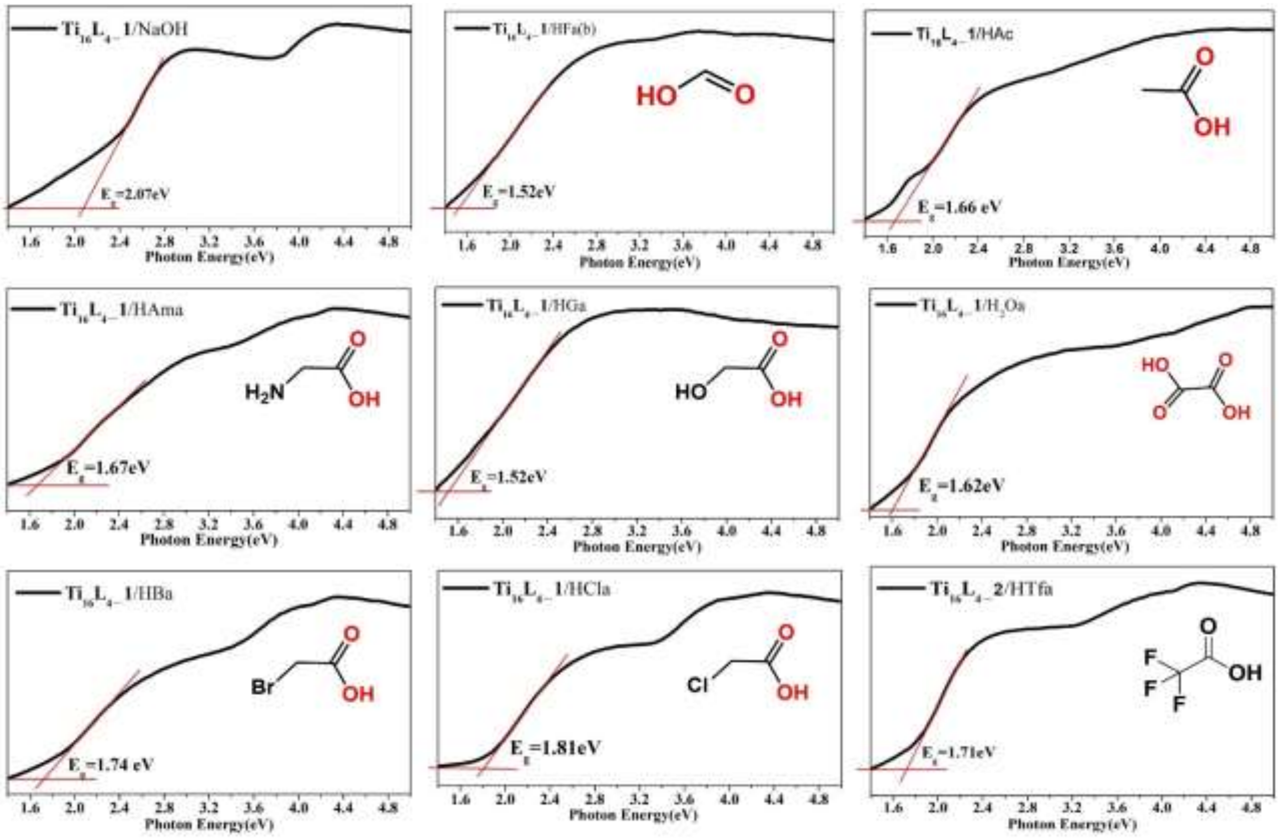

Figure S42. Tauc plot of the derived cluster of **$Ti_{16}L_4$_1** derived from UV-vis spectrum.

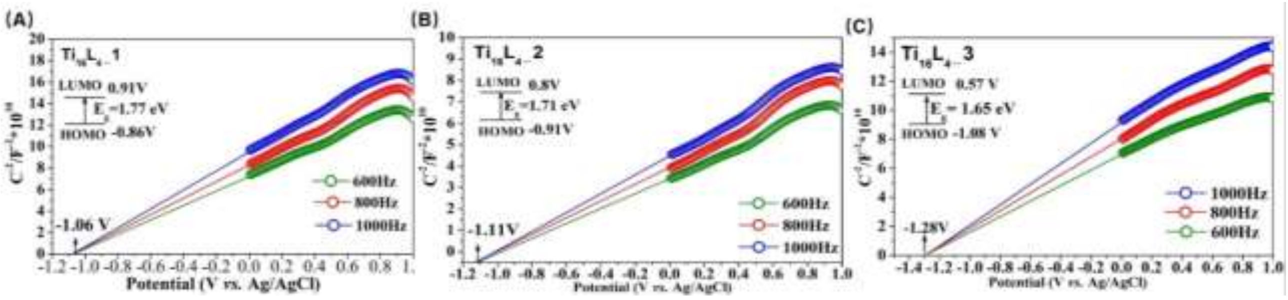

Figure S43. Mott−Schottky plots for **$Ti_{16}L_4$-1/2/3** in 0.2 M Na_2SO_4 aqueous solution. Insets are the energy diagrams of the HOMO and LUMO levels.

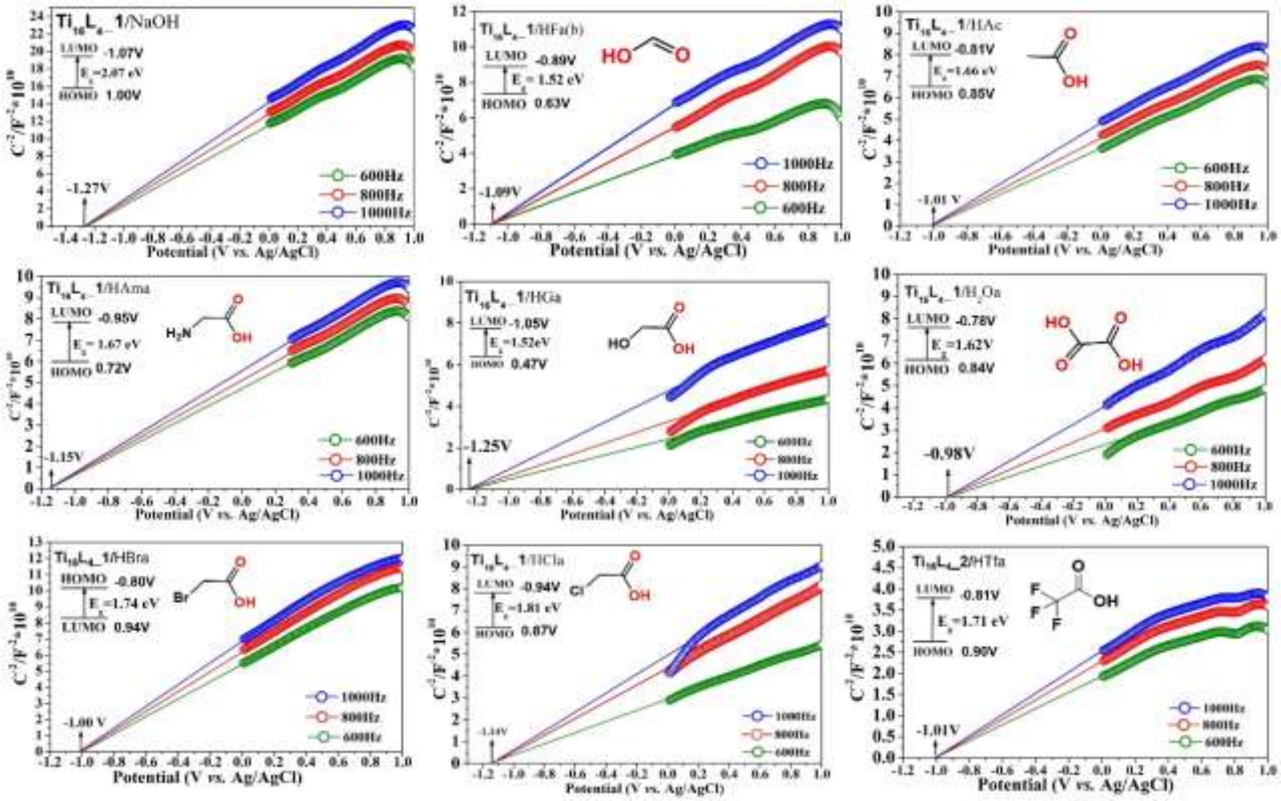

Figure S44. Mott−Schottky plots for the derived cluster of **$Ti_{16}L_4$-1** in 0.2 M Na_2SO_4 aqueous solution. Insets are the energy diagrams of the HOMO and LUMO levels.

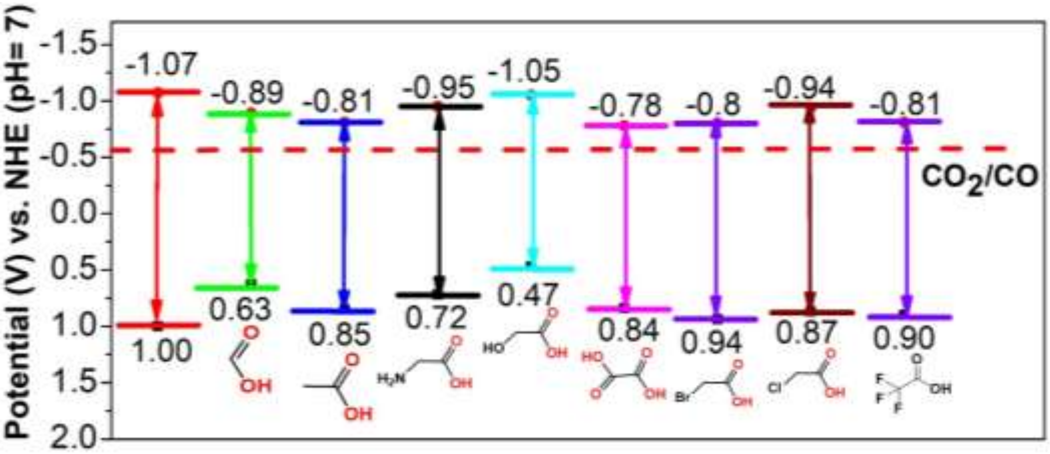

Figure S45. Band structure diagram for the derived cluster of $Ti_{16}L_4$.

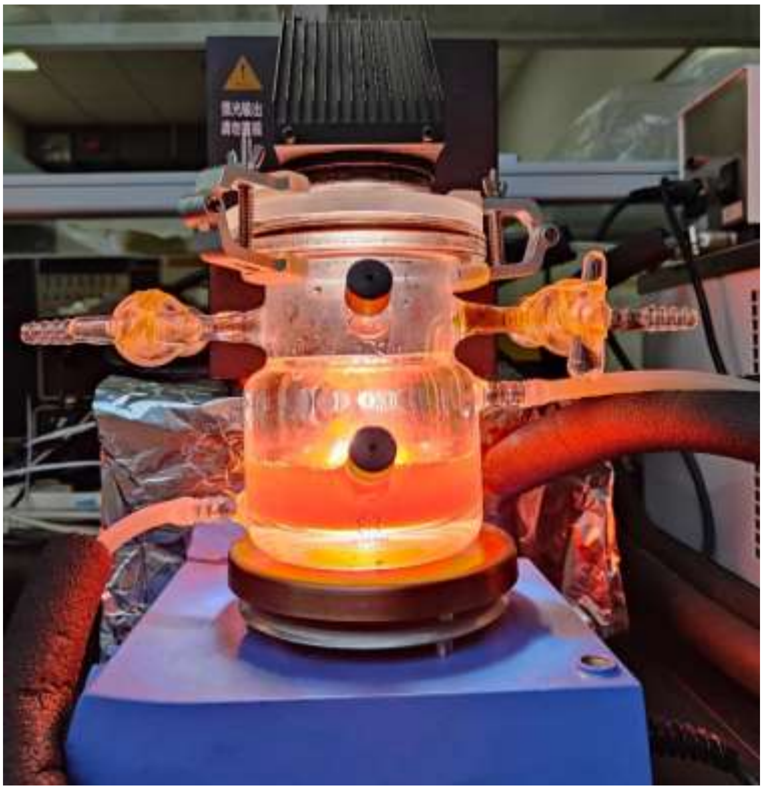

Figure S46. The picture of photocatalytic reactor.

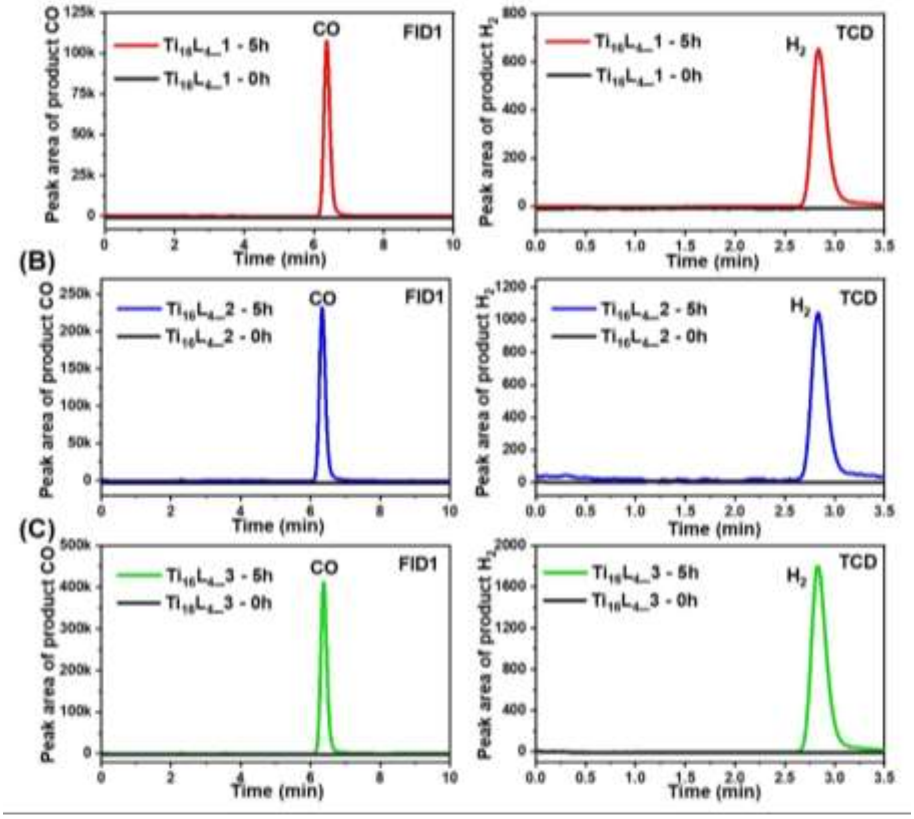

Figure S47. GC analysis of the gaseous reaction products for **$Ti_{16}L_{4}$_1/2/3** by using the FID and TCD.

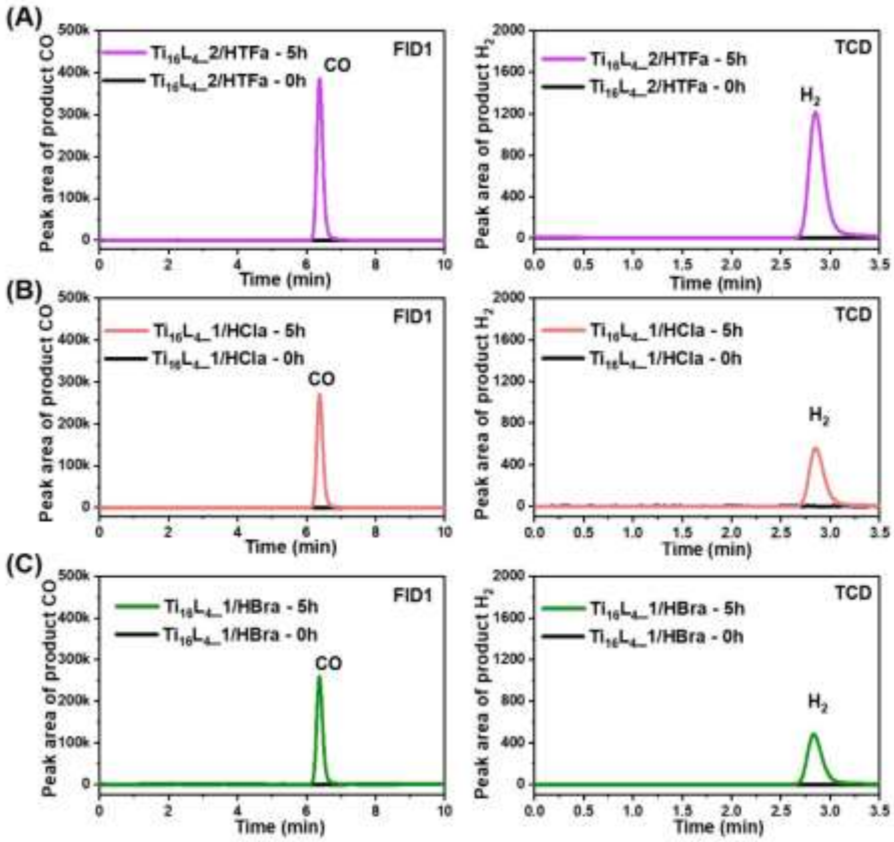

Figure S48. GC analysis of the gaseous reaction products for **$Ti_{16}L_4$_2/HTFa**、**$Ti_{16}L_4$_1/HCla** and **$Ti_{16}L_4$_2/HBra** by using the FID and TCD.

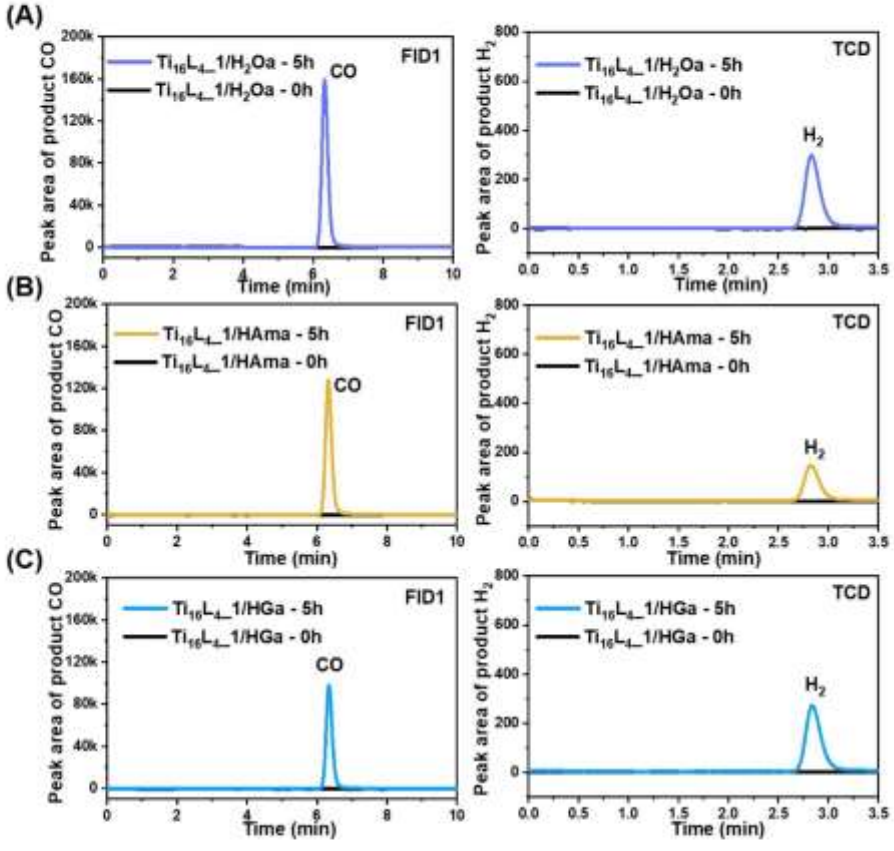

Figure S49. GC analysis of the gaseous reaction products for **$Ti_{16}L_4$_1/H_2Oa**、**$Ti_{16}L_4$_1/HAma** and **$Ti_{16}L_4$_1/HGa** by using the FID and TCD.

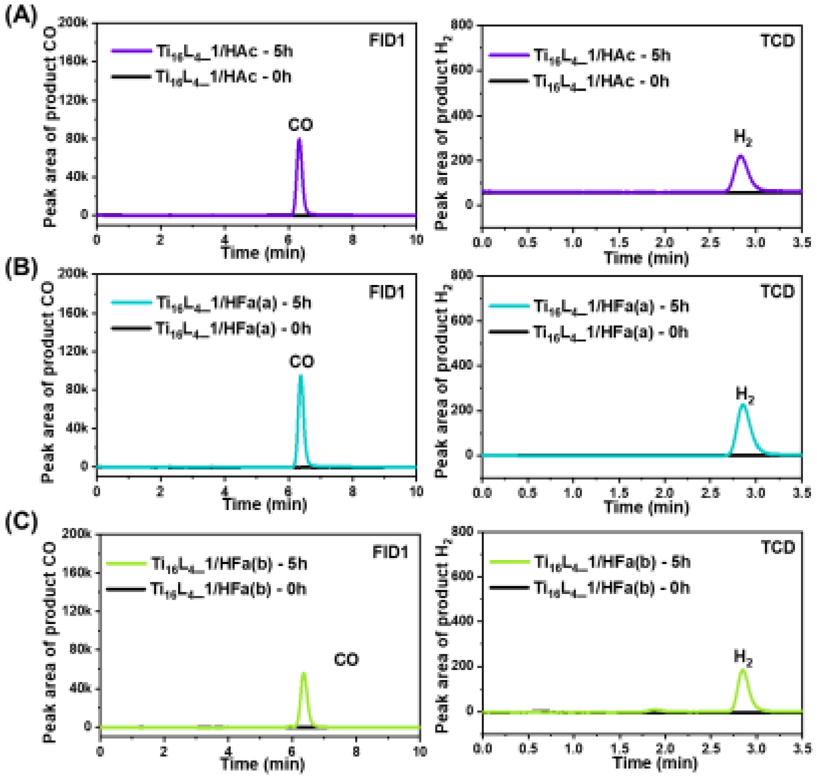

Figure S50. GC analysis of the gaseous reaction products for **$Ti_{16}L_4$_1/HAc**、**$Ti_{16}L_4$_1/HFa(a)** and **$Ti_{16}L_4$_1/HFa(b)** by using the FID and TCD.

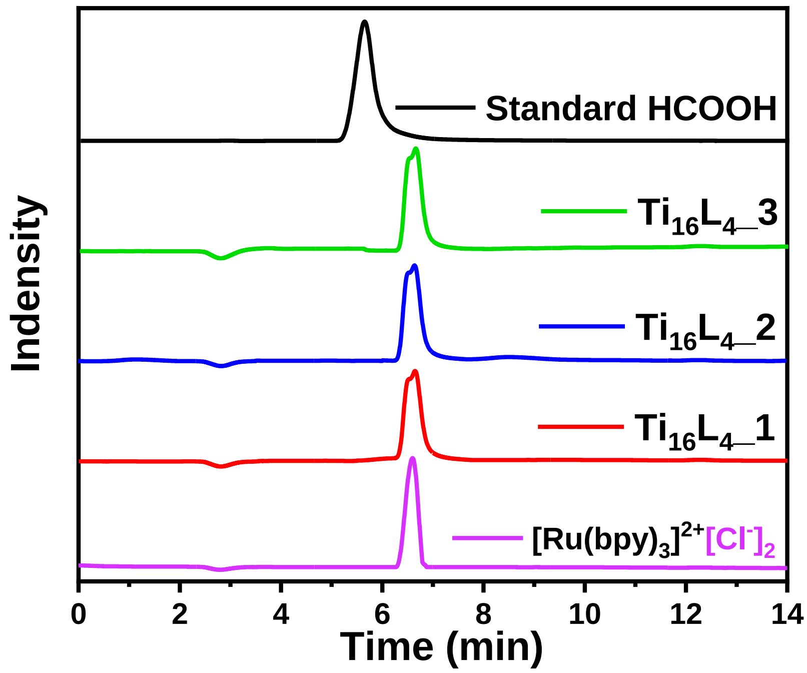

Figure S51. Analysis of the liquid reaction products generated in photocatalytic system by ion chromatography.

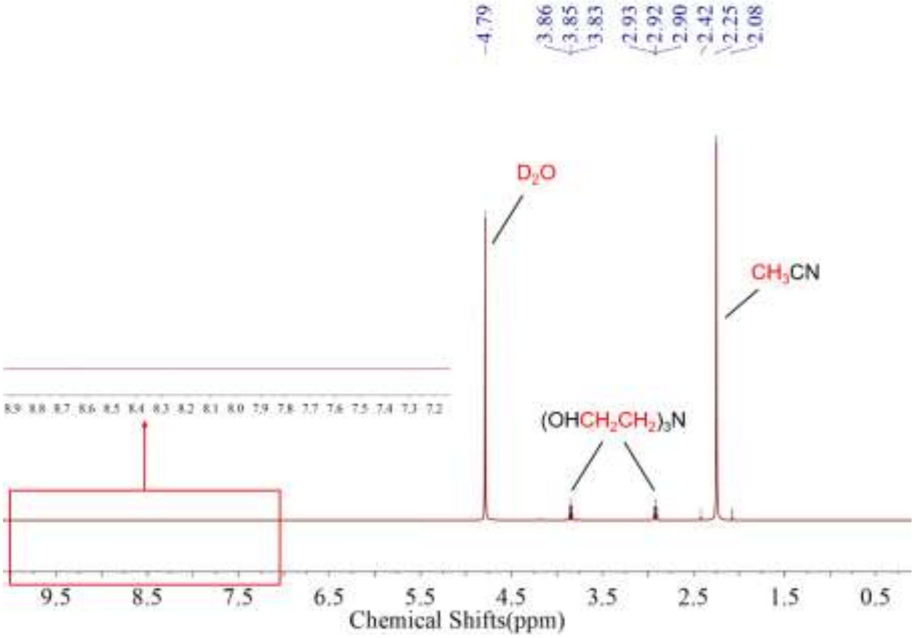

Figure S52. ^{1}H NMR spectrum of the liquid phase from the reaction system after visible-light irradiation.

Table S4. The photocatalysis results of the controlled experiments under different reaction conditions.

Entry	Cat.	Light	Photosensitizers	Sacrificial Agent	solvents	atmosphere	CO/μmol	H_2/μmol
1	**Ti$_{16}$L$_{4}$-1**	Xenon lamp	[Ru(bpy)$_3$]Cl$_2$·6H$_2$O	TEOA	CH_3CN/H_2O (4:1)	CO_2	12.02	0.07
2	**Ti$_{16}$L$_{4}$-2**	Xenon lamp	[Ru(bpy)$_3$]Cl$_2$·6H$_2$O	TEOA	CH_3CN/H_2O (4:1)	CO_2	29.99	0.44
3	**Ti$_{16}$L$_{4}$-3**	Xenon lamp	[Ru(bpy)$_3$]Cl$_2$·6H$_2$O	TEOA	CH_3CN/H_2O (4:1)	CO_2	60.57	2.34
4	×	Xenon lamp	[Ru(bpy)$_3$]Cl$_2$·6H$_2$O	TEOA	CH_3CN/H_2O (4:1)	CO_2	0.325	0
5	**Ti$_{16}$L$_{4}$-1**	Dark	[Ru(bpy)$_3$]Cl$_2$·6H$_2$O	TEOA	CH_3CN/H_2O (4:1)	CO_2	0	0
6	**Ti$_{16}$L$_{4}$-1**	Xenon lamp	[Ru(bpy)$_3$]Cl$_2$·6H$_2$O	×	CH_3CN/H_2O (4:1)	CO_2	0.467	0.125
7	**Ti$_{16}$L$_{4}$-1**	Xenon lamp	[Ru(bpy)$_3$]Cl$_2$·6H$_2$O	TEOA	CH_3CN/H_2O (4:1)	N_2	0	0.803
8	**Ti$_{16}$L$_{4}$-1**	Xenon lamp	Tris(2-phenylpyridinato)iridium	TEOA	CH_3CN/H_2O (4:1)	CO_2	0.239	0.968
9	**Ti$_{16}$L$_{4}$-1**	Xenon lamp	[Ru(bpy)$_3$]Cl$_2$·6H$_2$O	TEOA	H_2O	CO_2	0.139	0.868

[a] Reaction conditions: catalyst (3mg), photosensitizer (10 mg), sacrificial agent (TEOA, 5 mL), solvent (10 mL), reaction time(5 h);

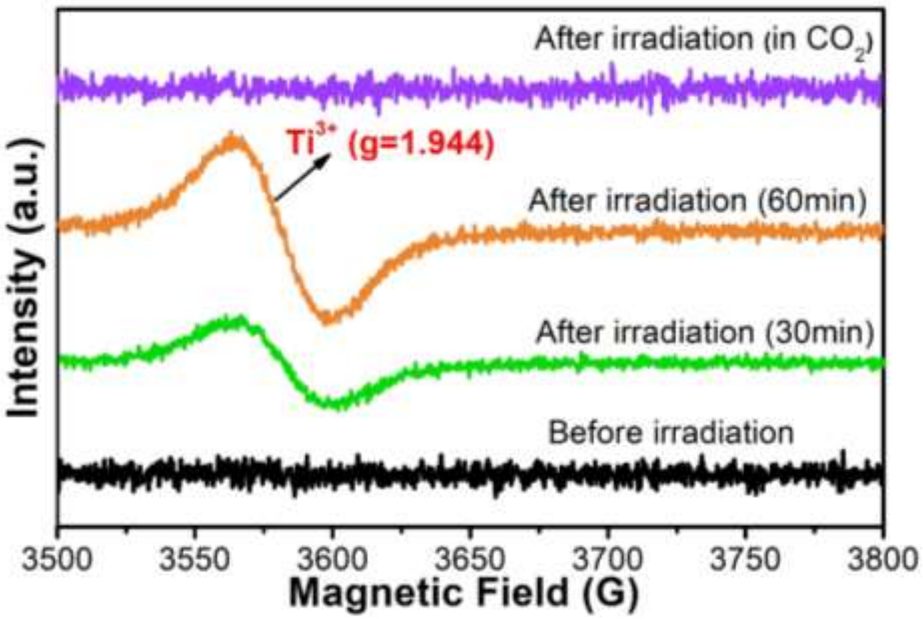

Figure S53. ESR spectrum of **Ti$_{16}$L$_4$_1** cluster before and after light irradiation. ESR spectra were obtained on a Bruker A300 spectrophotometer at 298 K. EPR spectra were obtained using 1−20 mW microwave power and 100 kHz field modulation with the amplitude set to 1 G. The g-values for each EPR spectrum were extracted from simulations performed using EasySpin (v5.2.23).

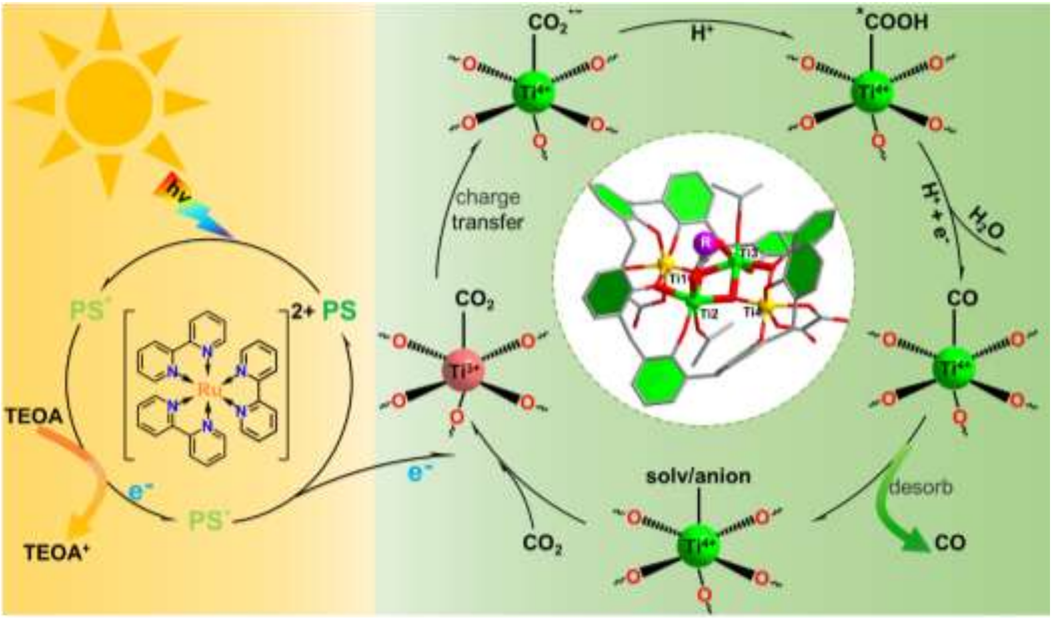

Figure S54. Reaction mechanism diagram.

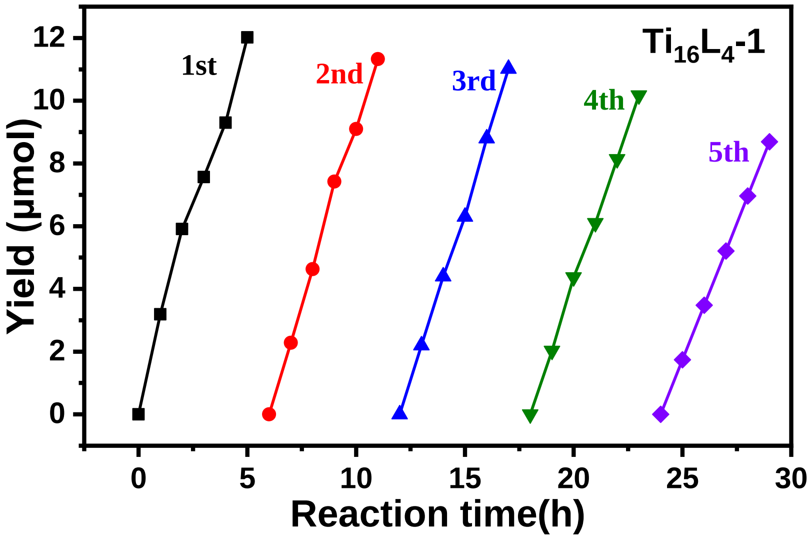

Figure S55. The photocatalysis results of the controlled experiments under different reaction conditions.

Table S5. The catalytic activity comparison of potential active sites in **$Ti_{16}L_4$_1/2/3.**

Entry	CO yield	TON	TON_{Ti}
$Ti_{16}L_4$_3	50.67 μmol	120.69	15.09
$Ti_{16}L_4$_2	29.99 μmol	70.96	8.87
$Ti_{16}L_4$_1	12.02 μmol	28.25	3.53

Turnover number (TON) = mol (CO yield) / mol (catalyst)

Turnover number (TON_{Ti}) = mol (CO yield) / mol (Ti^{4+} active sites)

We have demonstrated through experiments that $Ti_{16}L_4$ has 8 flexible coordination sites, namely active sites.

Table S6. Performance comparison of cluster-based photocatalysts for photocatalytic CO_2 reduction.

Photocatalyst	Photosensitizers	Sacrificial Agent	Reaction solvent	CO/μmol	H_2/μmol	Reference
Co-MOF-74	$[Ru(bpy)_3]Cl_2·6H_2O$	TEOA	Acetonitrile/H_2O	11.7/0.5 h	7.3/0.5 h	1
Mn-MOF-74	$[Ru(bpy)_3]Cl_2·6H_2O$	TEOA	Acetonitrile/H_2O	1.5/0.5 h	2.9/0.5 h	1
Zn-ZIF-8	$[Ru(bpy)_3]Cl_2·6H_2O$	TEOA	Acetonitrile/H_2O	2.1/0.5 h	2.4/0.5 h	1
Co_6-MOF	$[Ru(bpy)_3]Cl_2·6H_2O$	TEOA	Acetonitrile/H_2O	39.36/2 h	28.13/2 h	1
Co-ZIF-9	$[Ru(bpy)_3]Cl_2·6H_2O$	TEOA	Acetonitrile/H_2O	41.8/0.5 h	29.9/0.5 h	2
CuNi-L^2	$[Ru(bpy)_3]Cl_2·6H_2O$	TEOA	Acetonitrile/H_2O	4.6/8 h	0.32/8 h	3
TCOF-$MnMo_6$	without	without	H_2O	7.97/24 h	—	4
RO-4	without	without	H_2O	0.4/4 h	—	5
$Co(P_4Mo_6)$	$[Ru(bpy)_3]Cl_2·6H_2O$	TEOA	H_2O	0.32/10 h	0.012/10 h	6
Co_{16}-V_4	$[Ru(phen)_3](PF_6)_2$	TEOA	H_2O	3.41/10 h	—	7
Au@NENU-10	without	without	H_2O	1.28/5 h	0.26/5 h	8
BIF-29	$[Ru(bpy)_3]Cl_2·6H_2O$	TEOA	H_2O	166.7/5 h	35.0/5 h	9
$Ti_{16}L_4$-3	$[Ru(bpy)_3]Cl_2·6H_2O$	TEOA	Acetonitrile/H_2O	60.57/5 h	2.34/5 h	This work
Ti_{16}-C4A	without	TEOA	H_2O	HCOOH 136.0/12 h	—	10
PTi_{16}	without	TEOA	H_2O	HCOOH 24.13/18 h	0.1/18 h	11
Ti_6-Fcdc	without	TIPA	H_2O	HCOOH 35.0/10 h	—	12
Ti_4-C8A	without	TEOA	H_2O	HCOOH 41.51/17 h	1.07/17 h	13

Reference

1. Zhao, J., Wang, Q., Sun, C.Y., Zheng, T. T., Yan, L.K., Li, M. T., Shao, K. Z., Wang, X. L. & Su, Z. M. A hexanuclear cobalt metal–organic framework for efficient CO_2 reduction under visible light. *J. Mater. Chem. A*. 2017, **5**, 12498.
2. Wang, S. B., Yao, W. S., Lin, J. L., Ding Z. X. & Wang, X. C. Cobalt Imidazolate Metal–Organic Frameworks Photosplit CO_2 under Mild Reaction Conditions. *Angew. Chem. Int. Ed*. 2014, **53**, 1034-1038.
3. Yuan, L., Zhang, L., Li, X.-X., Liu, J., Liu, J.-J., Dong, L.-Z., Li, D.-S., Li, S.-L. & Lan, Y.-Q. Uncovering the synergistic photocatalytic behavior of bimetallic molecular catalysts. *Chinese Chem. Lett.* 2023, **34** 107146.
4. Li, Q., Chang, J.-N., Wang, Z., Lu, M., Guo, C., Zhang, M., Yu, T.-Y., Chen, Y., Li, S.-L., & Lan, Y.-Q. Modulated Connection Modes of Redox Units in Molecular Junction Covalent Organic Frameworks for Artificial Photosynthetic Overall Reaction. *J. Am. Chem. Soc.*, 2022, **144**, 1861−1871.
5. Li, X.-X., Zhang, L., Yuan, L., Wang, T., Dong, L.Z., Huang, K., Liu, J.& Lan, Y.-Q. Constructing crystalline redox catalyst to achieve efficient CO_2 photoreduction reaction in water vapor. *Chem. Eng. J.* 2022, **442**, 136157.
6. J. Du, Y. Ma, X. Xin, H. Na, Y. Zhao, H. Tan, Z. Han, Y. Li, Z. Kang, Reduced polyoxometalates and bipyridine ruthenium complex forming a tunable photocatalytic system for high efficient CO_2 reduction, *Chem. Eng. J.* 2020, **398** 125518.
7. Qiao, L., Song, M., Geng, A.& Yao, S. Polyoxometalate-based high-nuclear cobaltevanadiumeoxo cluster as efficient catalyst for visible light-driven CO_2 reduction, *Chin. Chem. Lett.* 2019, **30**, 1273e1276.
8. S.-M. Liu, Z. Zhang, X. Li, H. Jia, M. Ren, S. Liu, Ti-substituted keggin-type polyoxotungstate as proton and electron reservoir encaged into metaleorganic framework for carbon dioxide photoreduction, *Adv. Mater. Interfaces* 2018, **5**, 1801062.
9. Zhang, H.-X., Hong, Q.-L., Li, J., Wang, F., Huang, X., Chen, S., Tu, W., Yu, D., Xu, R., Zhou, T.& Zhang, J. Isolated Square-Planar Copper Center in Boron Imidazolate Nanocages for Photocatalytic Reduction of CO_2 to CO, *Angew. Chem. Int. Ed.* 2019, **58**, 11752–11756.
10. Li, N., Lin, J.-M., Li, R.-H., Shi, J.-W., Dong, L.-Z., Liu, J., He, J., & Lan, Y.-Q. Calix[4]arene-Functionalized Titanium-Oxo Compounds for Perceiving Differences in Catalytic Reactivity Between Mono- and Multimetallic Sites. *J. Am. Chem. Soc.* 2023, **145**, 16098–16108.
11. Li, N., Liu, J., Liu, J.-J., Dong, L.-Z., Li, S.-L., Dong, B.-X., Kan, Y.-H. & Y.Q. Lan, Self-As sembly of a phosphate-centered polyoxo-titanium cluster: discovery of the HeteroatomKeggin family, *Angew. Chem. Int. Ed.* 2019, **58**, 17260e17264.
12. Liu, J.-J., Li, N., Sun, J.-W., Liu, J., Dong, L.-Z., Yao, S.-J., Zhang, L., Xin, Z.-F., Shi, J.-W., Wang, J.-X., Li, S.-L., & Lan, Y.-Q. Ferrocene-Functionalized Polyoxo-Titanium Cluster for CO_2 Photoreduction. *ACS Catal.* 2021, **11**, 4510−4519.
13. Li, N., Liu, J.-J., Sun, J.-W., Dong, B.-X., Dong, L.-Z., Yao, S.-J., Xin, Z., Li, S.-L., & Lan, Y.-Q. Calix[8]arene-constructed stable polyoxo-titanium clusters for efficient CO_2 photoreduction. *Green Chem.* 2020, **22**, 5325-5332.

Table S7 Overview of photocatalytic CO production using $Ti_{16}L_4$ derivatives as catalysts.

Sample	$Ti_{16}L_4$-1	$Ti_{16}L_4$-2	$Ti_{16}L_4$-3	$Ti_{16}L_4$-1/NaOH	$Ti_{16}L_4$-1/HFa(a)	$Ti_{16}L_4$-1/HFa(b)	$Ti_{16}L_4$-1/HAc
CO production (μmol g^{-1} h^{-1})	798.37± 44.97	1998.10± 158.15	4046.67± 243.33	158.24 ± 85.28	911.71±115.65	636.23±19.36	773.87±43.91

Sample	$Ti_{16}L_4$-1/H_2Oa	$Ti_{16}L_4$-1/HGa	$Ti_{16}L_4$-1/HBra	$Ti_{16}L_4$-1/HCla	$Ti_{16}L_4$-1/HAma	$Ti_{16}L_4$-2/HTfa
CO production (μmol g^{-1} h^{-1})	1535.04±101.19	961.19±89.20	2404.16±172.73	2632.14±210.71	1228.07±140.51	3879.07±207.31

Table S8: Crystallographic data of **$Ti_{16}L_4$-1** after photocatalytic reaction (after five cycle).

Conditions	as-synthesized	After photocatalytic reaction
CCDC	2174429	2287594
Cryst. syst.	monoclinic	monoclinic
Space group	C2/c	C2/c
A[Å]	39.1900(19)	39.0491(14)
b[Å]	32.5046(16)	32.3507(12)

c[Å]	38.1646(18)	37.9914(14)
β/°	115.461(2)	115.3290(10)
V[Å^3]	43894(4)	43379(3)
Goof	43894(4)	1.054
R1/wR$_2$(I> 2σ(I))	0.0997/0.2961	0.0966/0.2770
R1/wR$_2$(all data)	0.1166/0.3132	0.1124/0.2955

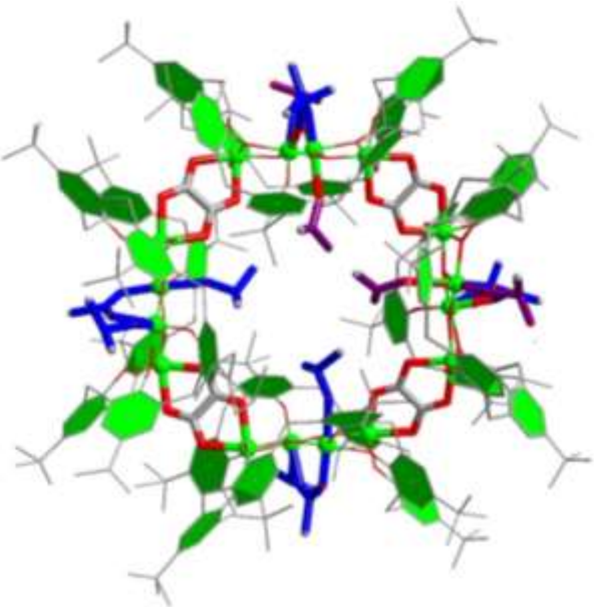

Figure S56. Crystallographic data of **Ti$_{16}$L$_4$-1** after photocatalytic reaction (after five cycle).

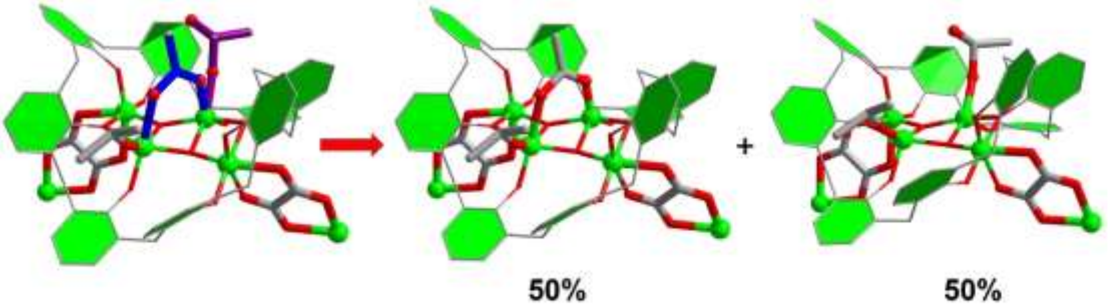

Figure S57. Crystallographic data of **Ti$_{16}$L$_4$-1** after photocatalytic reaction (after five cycle).

Discussion: After the photocatalytic reaction, the macrocyclic structure of Ti$_{16}$L$_4$-1 remained stable, but the surface ligand changed. The ligands in the two {Ti$_4$-TBC[8]} subunits remain unchanged and are still occupied by one μ$_2$-bridging acetate and two ^{i}PrO$^-$ groups. However, in the other two {Ti$_4$-TBC[8]} subunits, the coordination mode of half of the acetate groups changed.

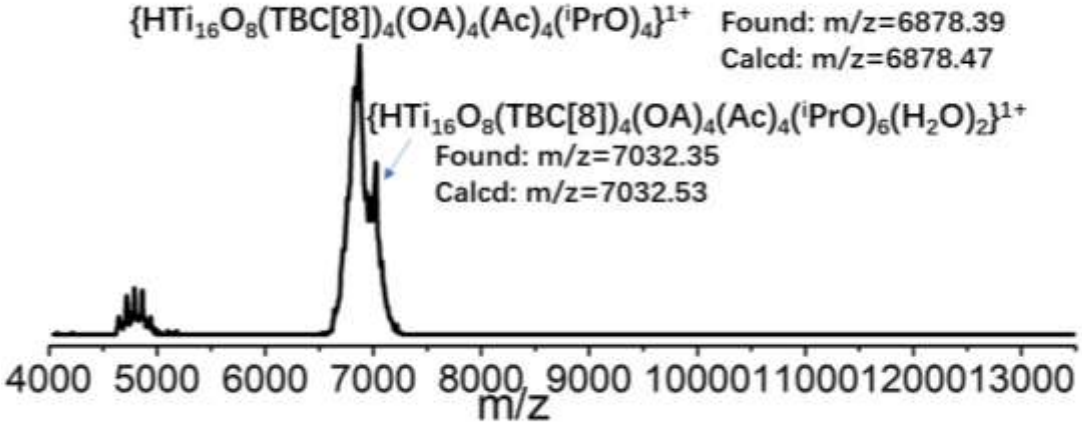

Figure S58. Positive-mode MALDI-TOF-MS spectrum of the crystalline sample of **$Ti_{16}L_4$_1** after five catalytic cycle.

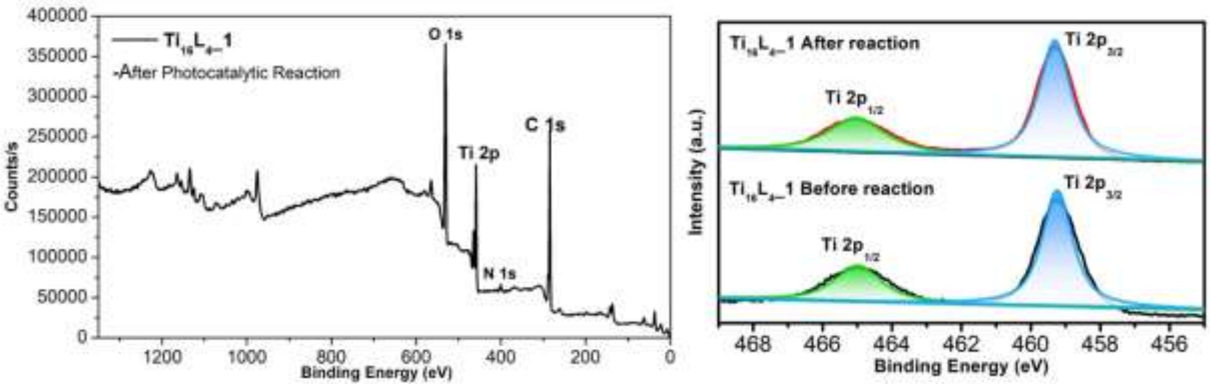

Figure S59. XPS of **$Ti_{16}L_4$_1** after catalysis tests.

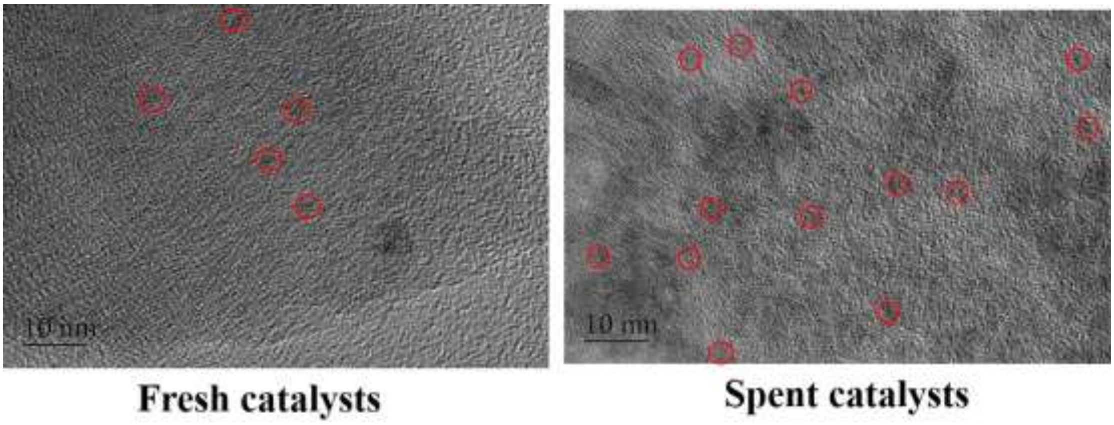

Figure S60. TEM image of **$Ti_{16}L_4$_1** before and after reaction.
